# Square-Planar Nickel Bis(phosphinopyridyl) Complexes for Long-Lived Photocatalytic Hydrogen Evolution

**DOI:** 10.1021/jacsau.4c00714

**Published:** 2024-09-26

**Authors:** Chien-Ting Wu, Hung-Ruei Pan, Chi-Tien Hsieh, Yu-Syuan Tsai, Pei-Juan Liao, Shuo-Huan Chiang, Che-Min Chu, Wei-Kai Shao, Yi-Rong Lien, Yu-Wei Chen, Tsung-Lun Kan, Vincent C.-C. Wang, Mu-Jeng Cheng, Hua-Fen Hsu

**Affiliations:** † Department of Chemistry, National Cheng Kung University, Tainan 701, Taiwan; ‡ Department of Chemistry, National Sun Yat-Sen University, Kaohsiung 804, Taiwan; § Instrument Center of National Cheng Kung University, Tainan 701, Taiwan

**Keywords:** nickel complex, hydrogen evolution, photolytic catalysis, phosphinopyridyl ligand, density functional theory

## Abstract

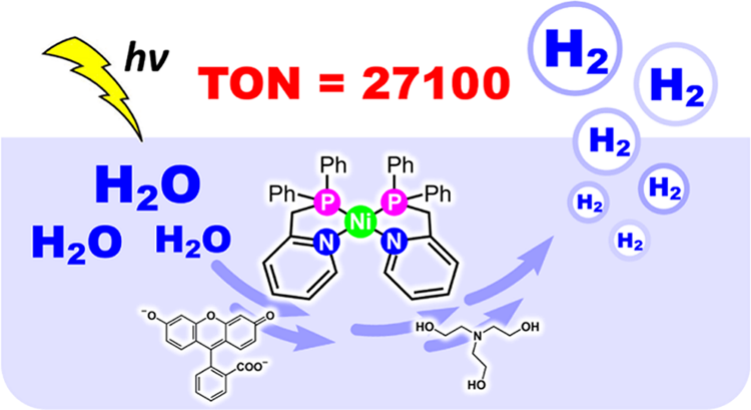

Phosphinopyridyl ligands are used to synthesize a class of Ni(II) bis(chelate) complexes, which have been comprehensively characterized in both solid and solution phases. The structures display a square–planar configuration within the primary coordination sphere, with axially positioned labile binding sites. Their electrochemical data reveal two redox couples during the reduction process, suggesting the possibility of accessing two–electron reduction states. Significantly, these complexes serve as robust catalysts for homogeneous photocatalytic H_2_ evolution. In a system utilizing an organic photosensitizer and a sacrificial electron donor, an optimal turnover number of 27,100 is achieved in an alcohol–containing aqueous solution. A series of photophysical and electrochemical measurements were conducted to elucidate the reaction mechanism of photocatalytic hydrogen generation. Density function theory calculations propose a catalytic pathway involving two successive one–electron reduction steps, followed by two proton discharges. The sustained photocatalytic activity of these complexes stems from their distinct ligand system, which includes phosphine and pyridine donors that aid in stabilizing the low oxidation states of the Ni center.

Artificial photosynthesis (AP) offers a sustainable avenue to convert solar energy into fuels.^1^ For example, producing hydrogen directly from benign water through an AP system is highly desirable.^2,3^ An artificial photosynthetic system for hydrogen production comprises two essential components: a photosensitizer (PS) and a catalyst.^4^ Developing these components using earth-abundant elements is crucial for achieving sustainability in an AP system.^5^ As a result, significant efforts have been made to use first-row transition metal complexes, such as Fe, Co, and Ni, as hydrogen–evolving catalysts (HECs) and metal-free photosensitizers in AP systems.^5−12^ However, most of these systems suffer from catalytic instability and only reach a turnover number (TON) of hundreds to thousands. Among reported examples of HECs with commonly used organic and inorganic synthetic dyes, a cobalt complex with a softer polypyridyl ligand, [Co(Py_3_Me-Bpy)(OH_2_)](PF_6_)_2_, has a high TON (15,000) by using Ru(bpy)_3_^2+^ as a PS.^13^ Chalcogenide quantum dots have significantly enhanced catalytic performance when replacing organic and inorganic dyes as PSs;^14−16^ however, they introduce disadvantages such as elemental scarcity, high cost, and toxicity.

Nickel catalysts are of significant interest for hydrogen evolution due to their relevance to [NiFe]—hydrogenases,^17^ the abundance of nickel on earth, and their lower cost in comparison to noble metals. Additionally, Ni compounds are accessible at diverse oxidation states, thereby demonstrating versatile reactivity and efficient catalytic performance in a variety of organic syntheses.^18,19^ Eisenberg, Holland, and their co–workers first investigated Ni(II) bis(diphosphine) complexes that were initially reported as electrocatalysts for H_2_ generation by DuBois and co–workers, yielding a TON of 2700 (Scheme 1a).^20−22^ Later, the same group further reported other Ni(II) bis(chelate) catalysts with various bidentate ligands, including benzenedithiolate, 2–aminobenzenethiolate, 2–mercaptophenolate, and mercaptopyridyl–*N*–oxide (Scheme 1b–d).^16^ These systems exhibited a TON of 5600–6190 when an organic dye, fluorescein (Fl) was used as the PS and a TON of over 280,000 when CdSe quantum dots were substituted for the dye. Ni(II) tris(pyridinethiolate) and bis(pyridinethiolate)(bipyridine) complexes were also evaluated for their activity by the same group, with a TON of 7300 (Scheme 1e–f).^23,24^ Additionally, Verani and co–workers reported a Ni catalyst with a polypyridyl ligand, giving a TON of 3500 (Scheme 1g).^25^

**Scheme 1:**
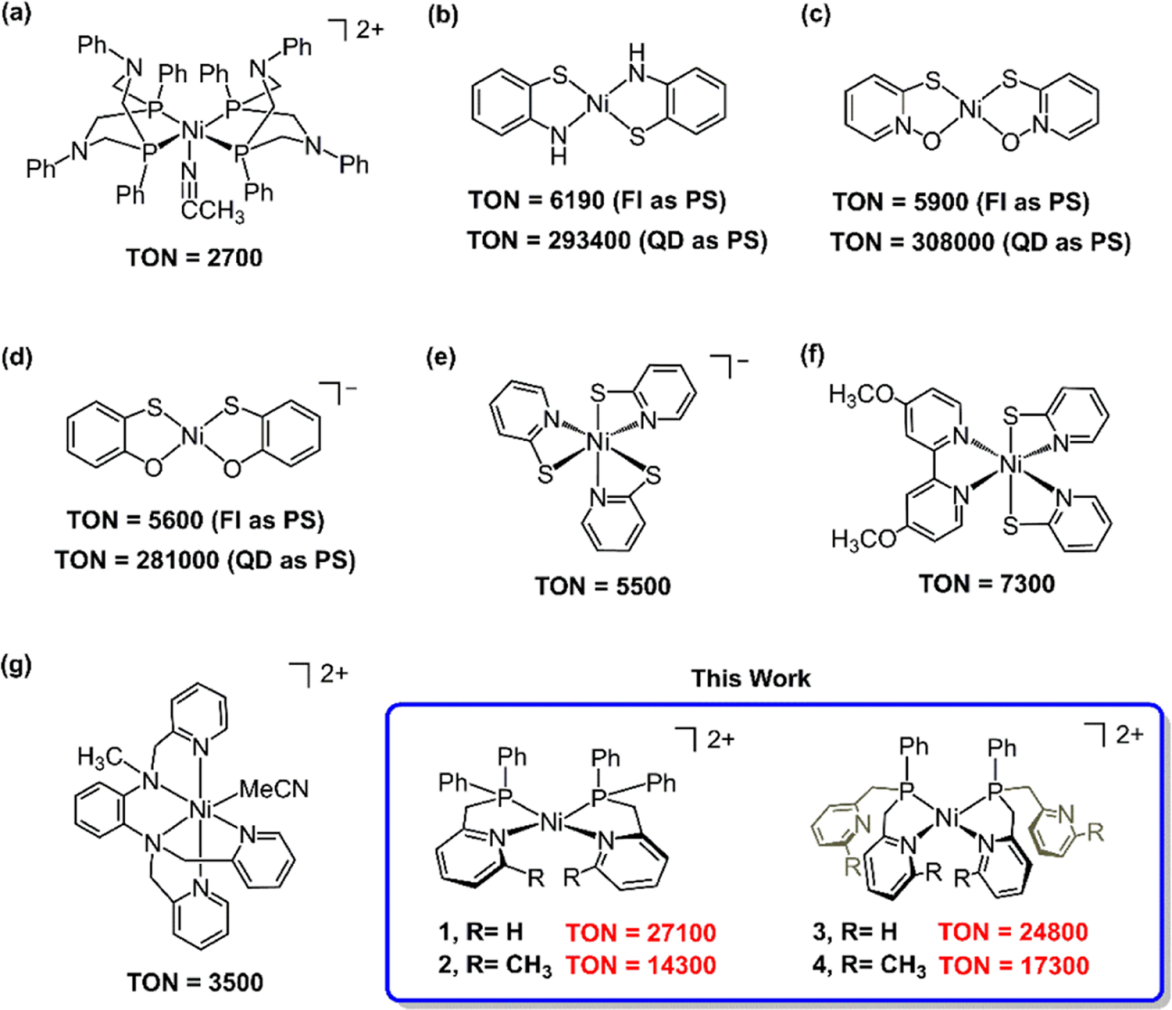
Ni Complexes for Photocatalytic H_2_ Evolution Reported in the Literature and This Work

The search for a robust nickel catalyst that can produce hydrogen economically from inexpensive organic dyes is what inspired our study. Optimizing the properties of a supporting ligand is critical for maintaining the stability of metal catalysts throughout the catalytic cycle. For this aim, we specifically synthesized and characterized a class of Ni(II) bis(chelate) complexes that feature phosphinopyridyl ligand derivatives, PN1, PN1^Me^, PN2, and PN2^Me^ (Scheme 1). The underlying hypothesis is that both phosphine and pyridine donors, with their unique π–back–donating characteristics, can enhance the accessibility of low–valent oxidation states at the Ni center. The addition of a 6–methyl substituent to pyridine donor ligands (PN1^Me^ and PN2^Me^) allows for the introduction of steric effects and likely further tuning of the electronic properties in nickel complexes, as inspired by iron chemistry involving tris(pyridyl)amine (TPA) derivatives.^26^ While previous research has explored a similar ligand motif, 6–((diphenylphosphino)–methyl)pyridin–2–amine of Ni complexes, for electrocatalytic hydrogen production,^27,28^ their photocatalytic activity for hydrogen evolution reaction (HER) was not investigated yet. To the best of our knowledge, our system outperforms other first-row transition–metal complexes in terms of durability in hydrogen evolution while utilizing organic dyes.^8^

## Results and Discussion

### Syntheses and Characterization

**Figure 1:**
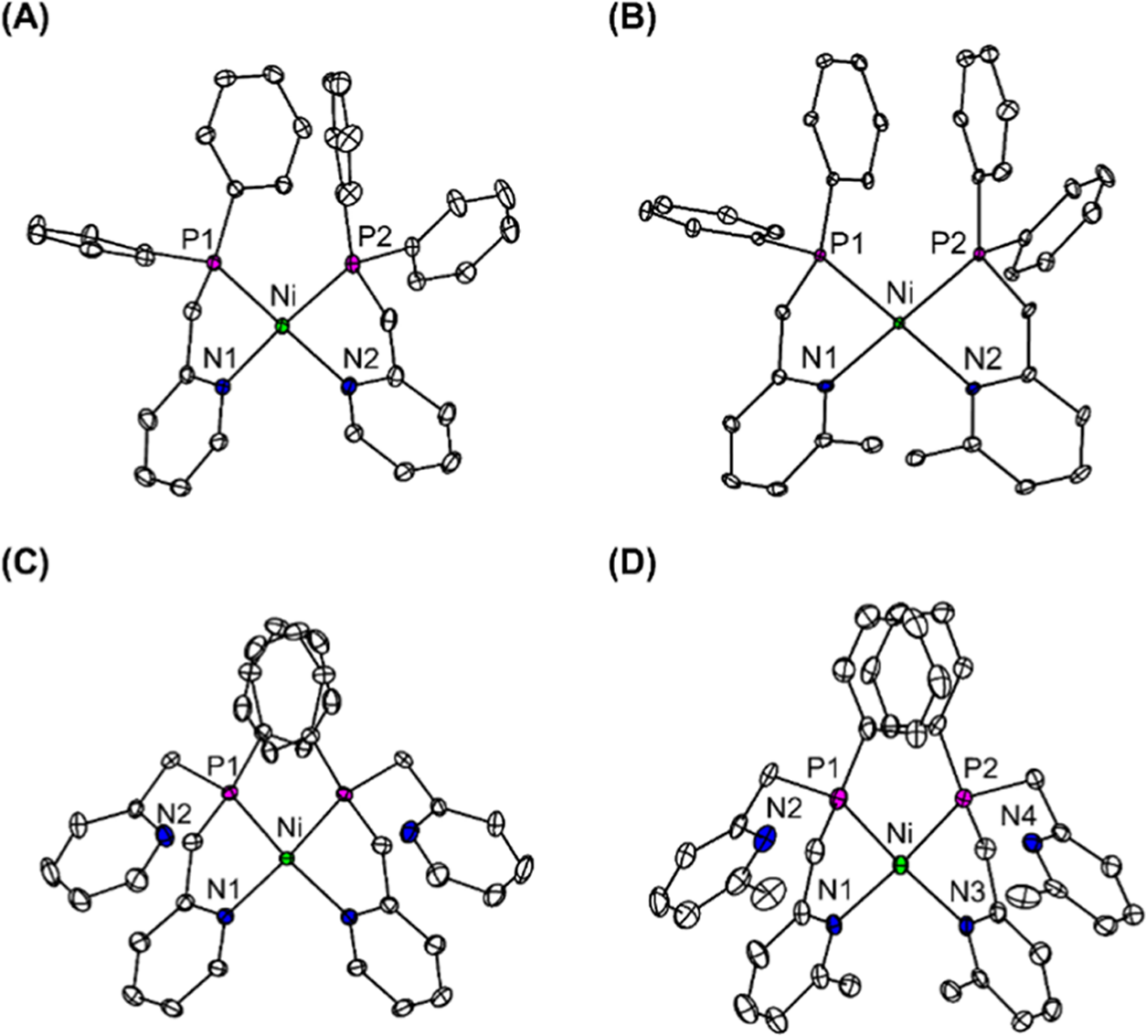
ORTEP diagrams of (A) [**1**][(ClO_4_)_2_], (B) [**2**][(ClO_4_)_2_], (C) [**3**][(ClO_4_)_2_] and (D) [**4**][(ClO_4_)_2_]·2.5CH_3_CN with a 35% probability. The anions ClO_4_^–^, H atoms and solvated molecules are omitted for clarity. The nickel ion is located in a crystallographic inversion center in [**3**][(ClO_4_)_2_]. Two Ni complexes are contained in an asymmetry unit with similar structural parameters in [**4**][(ClO_4_)_2_]·2.5CH_3_CN. Only one is shown, while the other is omitted.

The phosphinopyridyl ligand derivatives (PN1, PN1^Me^, PN2 and PN2^Me^) were synthesized in accordance with the procedures described in the literature.^30−35^ The reactions of Ni(ClO_4_)_2_ in CH_3_CN with the ligand derivatives resulted in nickel(II) bis(chelate) complexes. These complexes were designated as [Ni(PN1)_2_]^2+^ (**1**), [Ni(PN1^Me^)_2_]^2+^ (**2**), [Ni(PN2)_2_]^2+^ (**3**), and [Ni(PN2^Me^)_2_]^2+^ (**4**), respectively. When ether was layered into the reaction, brown crystalline solids of **1**–**4** with two ClO_4_^–^ anions were produced in high yields. The X–ray crystallographic structures have a slightly distorted square–planar geometry, with the Ni(II) centers binding to two phosphino and two pyridyl groups in a *cis* configuration (Figures 1 and S1–S4).^36^ As shown in Tables S1–S8, all complexes have relatively comparable bond distances and angles. The incorporation of a 6–methyl substituent into pyridine donor ligands (PN1 vs PN1^Me^ and PN2 vs PN2^Me^) does not induce structural differences within the first coordination sphere. Our density function theory (DFT) calculations indicate that even in the solvent the *cis* forms of complexes **1** and **2** are more stable than the corresponding *trans* forms by 0.13 eV in both cases, which is consistent with the observations of X-ray crystal structures. The pendant pyridyl groups of the ligands weakly interact with the Ni center in axial positions in complex **3**, as indicated by a Ni–N2 distance of 3.32 Å (Figure S3). In solution, this interaction becomes more pronounced, resulting in the formation of a six–coordinate Ni complex, as supported by spectroscopic studies (vide infra). In contrast, the pendant pyridines in complex **4** remain uncoordinated, possibly as depicted in the space–filling diagrams (Figure S5), due to the steric hindrance caused by the 6–methyl substituent, which prevents axial interaction.

The electronic spectra of **1**, **2**, and **4** are similar, with weak absorption bands at 424 nm (ε = 4.6 × 10^2^ M^–1^ cm^–1^), 428 nm (ε = 4.0 × 10^2^ M^–1^ cm^–1^) and 443 nm (ε = 3.2 × 10^2^ M^–1^ cm^–1^), respectively (Figure S6). The features with small molar extinction coefficients are assigned as d–d transitions, ^1^A_1g_ → ^1^E_g_.^37^ Complex **3**, on the other hand, has a distinct spectrum that includes a band at 475 nm (ε = 3.2 × 10^2^ M^–1^ cm^–1^) and a broad shoulder at 700–800 nm. These two absorptions are most likely attributed to d–d transitions in an octahedral geometry, ^3^A_2g_ → ^3^T_1g_ and ^3^A_2g_ → ^3^T_2g_, indicating that two weakly binding pyridyl groups shown in the crystal structure also have interaction with Ni(II) ion in the solution state, resulting in a six–coordinate Ni(II) center.^38^

**Figure 2:**
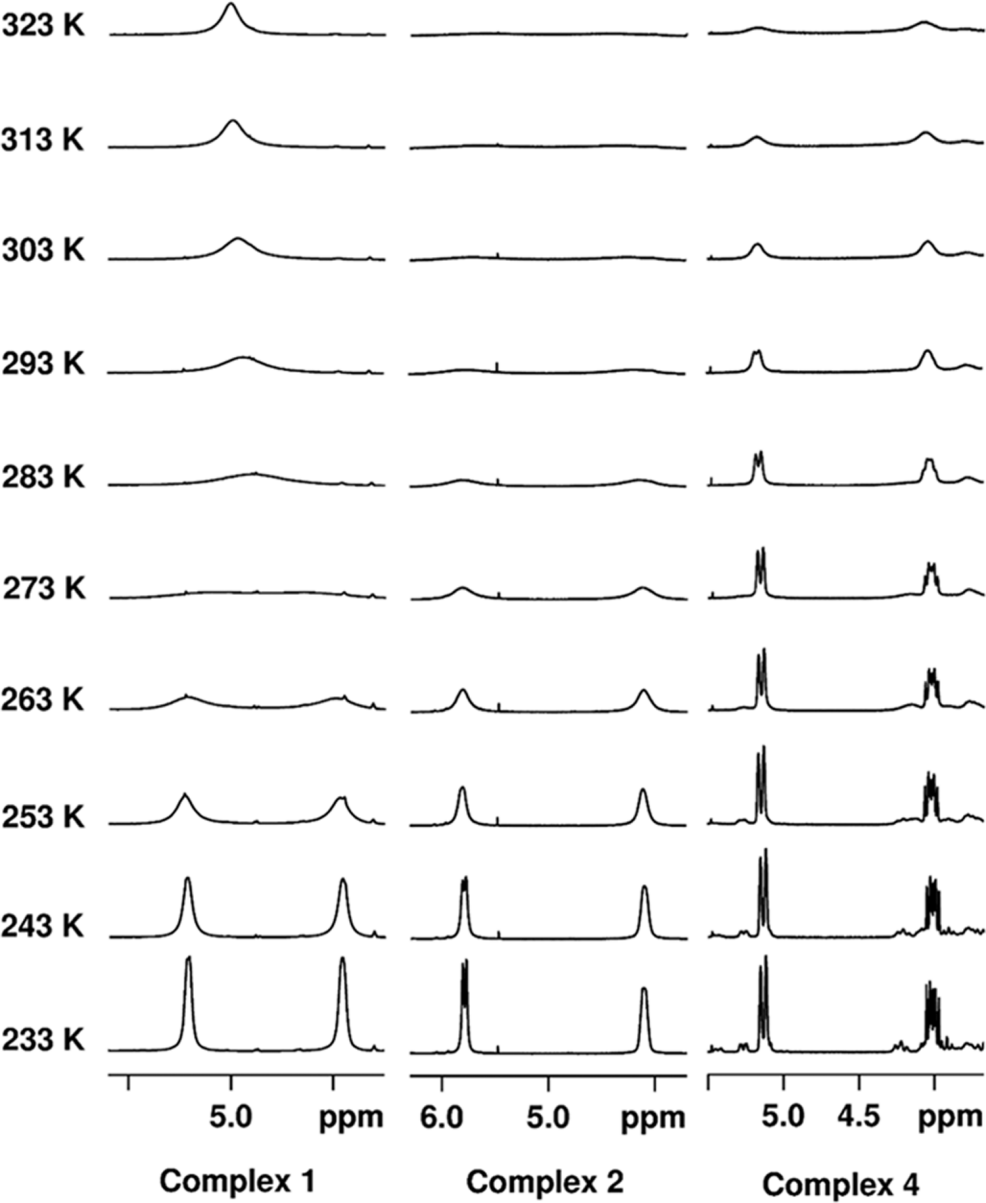
Variable–temperature ^1^H NMR spectra of complexes **1**, **2** and **4** at 3.5 to 6.3 ppm.

The variable–temperature (233 to 323 K) NMR spectroscopic investigations of **1**–**4** in CD_3_CN provided valuable insights into their dynamic and magnetic behavior in solution. At 233 K, the ^1^H NMR spectra of **1**, **2** and **4** revealed well–resolved signals within the diamagnetic range (0–10 ppm), consistent with a low–spin square–planar d^8^ configuration (Figures 2 and S7–S9). Further confirmation of peak assignments was made through two-dimensional (2D) correlation experiments, as detailed in the Supporting Information and Figures S10–S18. In addition, the ^31^P NMR spectra of these three complexes exhibited peaks at 44.4, 54.6, and 50.4 ppm at 233 K, respectively (Figure S19). However, upon reaching room temperature, the signals gradually weakened, indicating the possible interconversion of different conformers induced by chelate ring dynamics. In contrast, the ^1^H NMR spectrum of **3** at 233 K displayed clear paramagnetic features in the range of 65 to −51 ppm, consistent with the identification of high–spin Ni(II) species in the electronic spectrum (Figure S20). Furthermore, the paramagnetic nature of **3** was evident in the significantly broader ^31^P NMR peak at 40.5 ppm at 233 K compared to those of **1**, **2**, and **4** (Figure S21). Utilizing the Evans method, the magnetic susceptibility of **3** in the solution state was determined to be 2.6 μB, implying a ground state with *S* = 1.

The variable–temperature NMR spectra further revealed the fluxional nature of **1**, **2**, and **4** in solution. At low temperatures, all displayed an AB pattern for the diastereotopic methylene protons of the ligands (Figure 2). However, as the temperature increased, the interconversion of different conformers, facilitated by chelate rings, became fast on the NMR time scale. The coalescence temperature for **1** was identified at 283 K, with a free energy of activation (Δ*G*^‡^) of 47.9 kJ/mol.^39−41^ Complexes **2** and **4** exhibited higher coalescence temperatures than 323 K, due to the methyl substituents on the pyridyl groups, which elevates Δ*G*^‡^ of the interconversion process. Similarly, the phenyl protons of the phosphine donors demonstrated comparable dynamic behavior.

### Electrochemical Behavior

**Figure 3:**
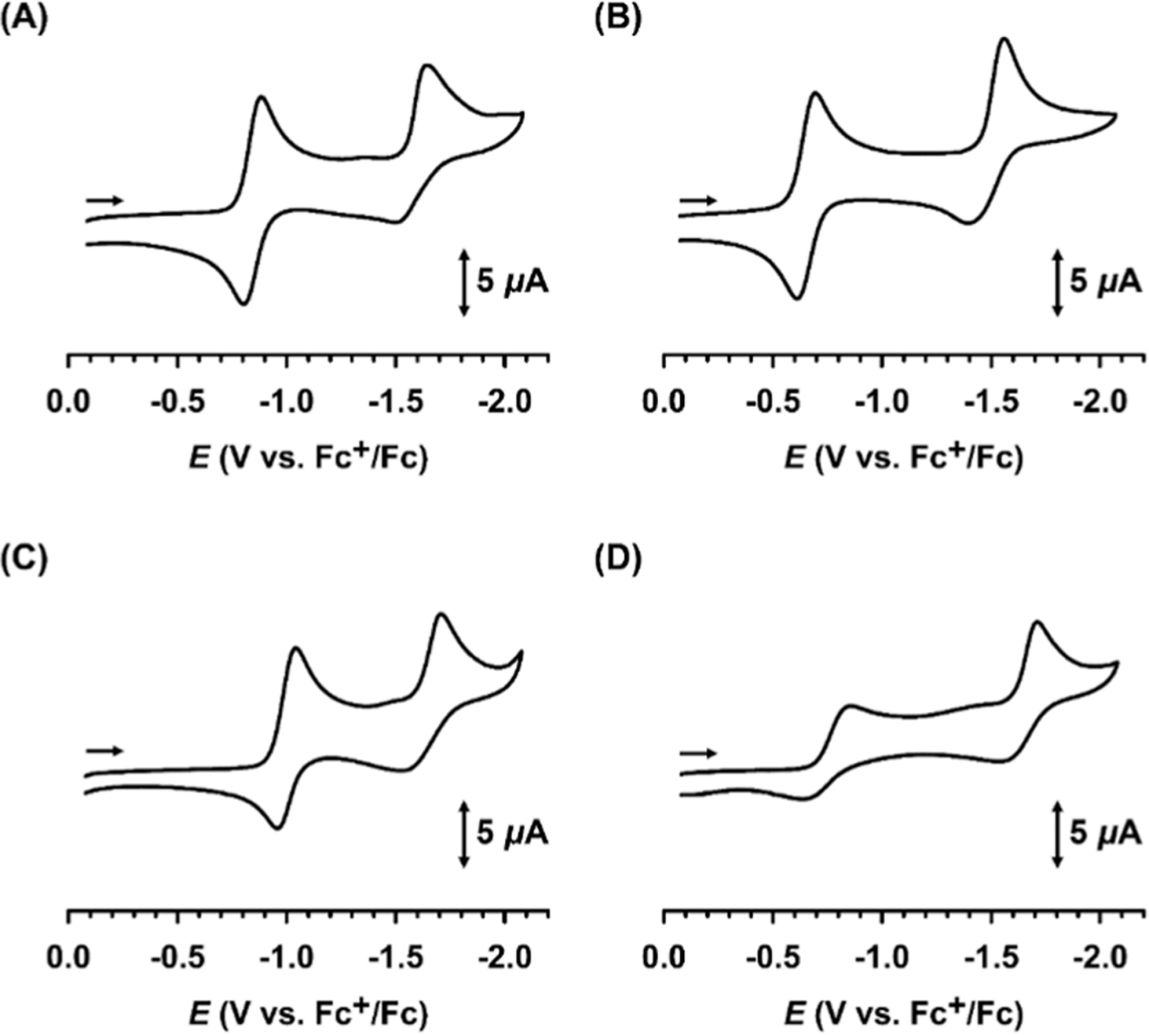
Cyclic voltammograms of **1**–**4** (A–D) in CH_3_CN. Conditions: NBu_4_PF_6_, Pt, and Ag/AgNO_3_ as the supporting electrolyte, working electrode, and reference electrode, respectively. Scan rate: 100 mV/s.

The electrochemical properties of **1**–**4** were investigated by cyclic voltammetry (CV) (Figure 3 and Table 1). During the reduction process, the voltammograms revealed the presence of two redox couples with *E*_1/2_ values ranging from −0.66 to −0.96 V and −1.48 to −1.63 V (vs Fc^+^/Fc). These results were further corroborated by differential pulse voltammetry measurements (Figure S22). These redox peaks are probably associated with the metal–centered redox couples, Ni(II/I) and Ni(I/0), as suggested by the absence of notable waves in this specific region in the CV data of the PN1 ligand (Figure S23). The former waves involving Ni(II/I) for **1** and **3** are quasi–reversible, whereas those for **2** and **4** are irreversible with larger Δ*E* values. The waves corresponding to the Ni(I/0) redox couple for all four complexes are irreversible, exhibiting significant peak-to-peak separations. The linear correlation between the square root of the scan rate and the reduction current (*i*_p_) suggests that the electrochemical process is governed by diffusion-controlled behavior (Figures S24–S27).

**Table 1:** Cyclic Voltammetry Data for **1**–**4** in CH_3_CN (V vs Fc^+^/Fc)^a^

complexes	*E*_1/2_^first^	Δ*E*^first^	*E*_1/2_^second^	Δ*E*^second^
**1**	–0.844	0.078	–1.573	0.149
**2**	–0.666	0.128	–1.486	0.216
**3**	–0.995	0.086	–1.628	0.155
**4**	–0.750	0.222	–1.629	0.157

a Conditions: NBu_4_PF_6_, Pt, and Ag/AgNO_3_ as the supporting electrolyte, working electrode, and reference electrode, respectively. Scan rate: 100 mV/s.

The incorporation of a 6–methyl substituent into pyridine donor ligands is most likely responsible for the irreversibility of Ni(II/I) in **4**. Density function theory (DFT) calculations suggest that this substitution destabilizes the Ni(I) state, potentially leading to ligand dissociation (vide infra). In contrast, the significant peak separation observed for Ni(I/0) couples in all complexes is most likely due to the complex reorganizing from a square planar to a tetrahedral geometry during reduction, as indicated by DFT calculations (vide infra). Moreover, introducing methyl substituents to the ligands leads to a more positive redox potential (**1** vs **2** and **3** vs **4**), contrary to the expected electronic donation effect. Iron and dimanganese complexes with TPA ligand derivatives show similar behaviors in redox potential upon the addition of a 6–methyl substituent.^26,42^ Pyridine–containing ligands with 6–methyl substituents have a preference for a lower oxidation–state metal center with a larger ionic radius, resulting in a more positive redox potential. Consequently, steric hindrance from the methyl substituent likely plays a crucial role in facilitating the attainment of lower Ni oxidation states. Moreover, the higher coordination number observed in **3**, as supported by electronic spectroscopic data, leads to a more negative potential in comparison to **1**. The redox potentials of square–planar Ni(II) complexes reported in the literature vary depending on the donor sets.^16,28,43^ Complexes **1**–**4** share similarities with [NiL_2_]^2+^ (where L = 6–((diphenylphosphino)–methyl)pyridin–2–amine) described by Masuda et al. in terms of their redox properties.^28^

### Photocatalytic Hydrogen Evolution Reaction

**Figure 4:**
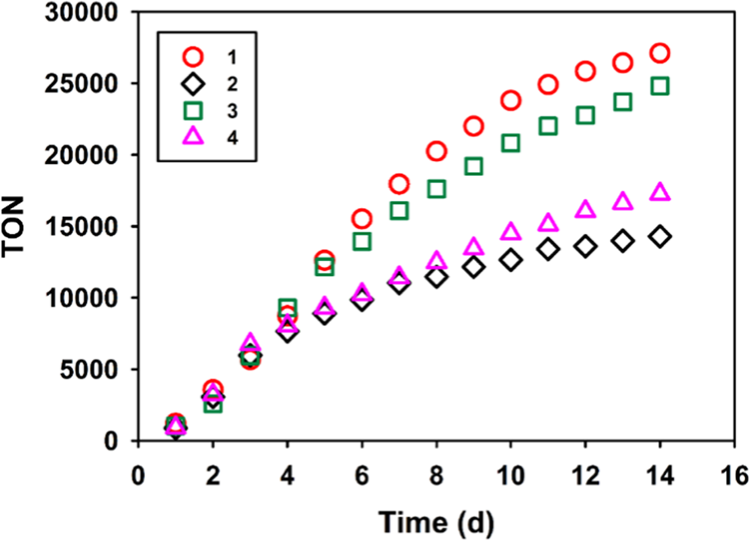
Photocatalytic hydrogen production from systems containing **1**–**4** (4.4 μM) as catalysts, Fl (18.6 mM) and TEOA (0.42 M). Reactions were carried out in MeOH/H_2_O (1:1) (for **1** and **2**) and EtOH/H_2_O (1:1) (for **3** and **4**) at pH 10.45 at room temperature upon irradiation (λ = 415–420 nm, 300 mW, LED).

Photocatalytic activity experiments of **1**–**4** for HER were conducted to determine optimal conditions by varying PS (Table S9), sacrificial electron donor (SED) (Table S10), solvent system (Table S11), pH value (Table S12) and catalyst concentration (Table S13). The quantification of hydrogen production was carried out using gas chromatography with thermal conductivity detector (GC–TCD). The presence of all three components (catalyst, PS, and SED) was necessary for the system to produce hydrogen. The catalytic reactions performed in an alcohol–mixed aqueous solution at pH = 10.45 with Fl as the PS and triethanolamine (TEOA) as the SED yielded the best TON (Figure 4 and Table S14). The production of H_2_ increased linearly for all complexes over the initial 4 days, with an average turnover frequency (TOF) ranging from 2000 to 2500 d^–1^. However, **2** and **4** exhibited a gradual decline in activity after 4 days, resulting in respective TONs of 14,300 and 17,300. In contrast, **1** and **3** showed a steady increase in H_2_ production until the eighth or ninth day, after which they began to decrease, resulting in TONs of 27,100 and 24,800, respectively. The apparent quantum yield is approximately 0.56%. Adding an additional equivalent of catalyst to the 14–day reaction catalyzed by **1** partially restored its activity, leading to a three–day average TOF of 1400 d^–1^ (Figure S28). This observation suggests that the catalyst undergoes decomposition during the catalytic cycle, possibly due to irradiation or the instability of Ni complexes at lower oxidation states (+1 or 0). The HER catalyzed by **1** was subjected to a mercury poisoning test. The results showed no significant difference between reactions with and without the addition of Hg (Figure S29), indicating that catalytic activity of **1** functions as a molecular catalyst rather than the formation of nanoparticles under photocatalytic conditions.^44^

Despite the fact that **2** and **4** have more positive reduction potentials than **1** and **3** by introducing 6–methyl substituents to the ligand, indicating easier access to the lower oxidation state of the Ni center, the initial catalytic performances of all complexes were comparable. However, **1** and **3** exhibited greater stability than **2** and **4**, resulting in greater TONs for the former. The differences in robustness observed among the catalysts may stem from various factors, including the stability and solubility of catalytic intermediates. According to electrochemical data, the reduction of Ni(II) to Ni(I) is less reversible in **2** and **4** compared to **1** and **3**; this suggests that the latter have more stable reduced states. Furthermore, the inclusion of 6–methyl substituents may lead to steric effects that result in instability in Ni(I) states, thereby diminishing the overall catalytic performance. DFT calculations also revealed the less stable Ni(I) state caused by the 6–methyl substituent (vide infra). Overall, **1** and **3** are the most robust molecular catalysts among non–noble metal complexes for photolytic HERs containing organic or inorganic dyes as PS.

### Mechanistic Studies and DFT Analysis

**Figure 5:**
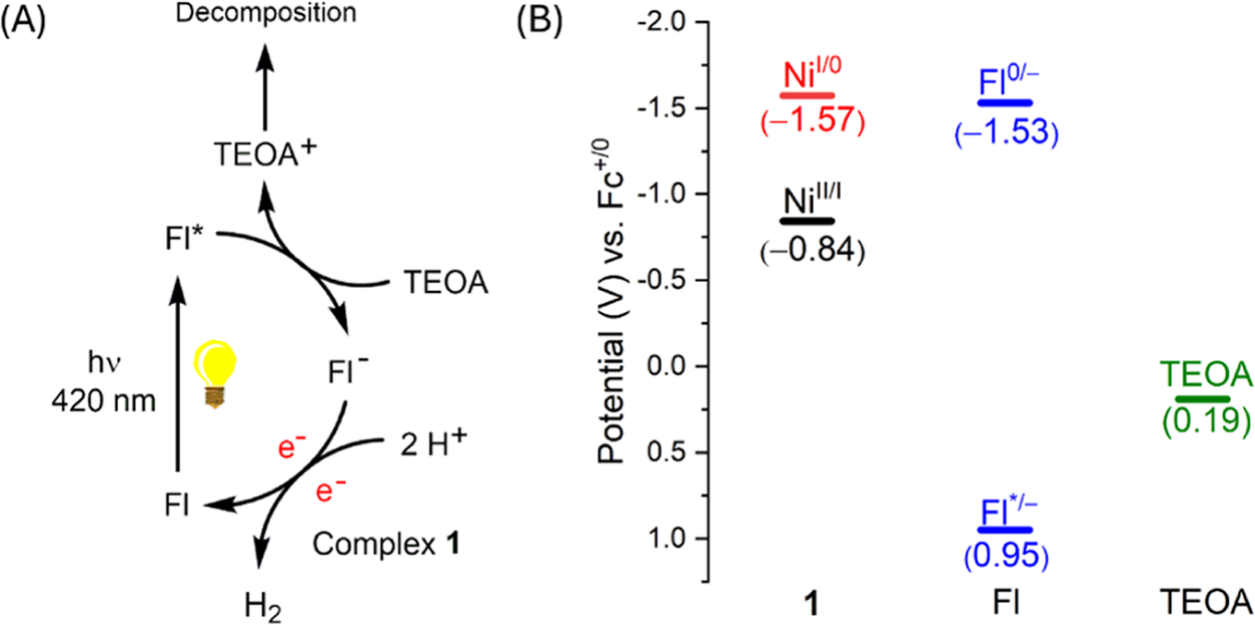
(A) Proposed photocatalytic mechanism for hydrogen evolution and (B) energy levels of relevant species in the photocatalytic reaction.

A series of photophysical and electrochemical measurements were conducted to elucidate the reaction mechanism of photocatalytic hydrogen generation. Initially, the quenching mechanism between Fl and complex **1**, as well as between Fl and TEOA, was investigated using Stern–Volmer quenching experiments. As shown in Figure S30, the fluorescence intensity of Fl diminished with increasing TEOA concentration in the solution. The bimolecular quenching constant *k*_q(TEOA)_ for TEOA is 1.16 × 10^9^ M^–1^ s^–1^. In contrast, the quenching constant in the presence of **1** was significantly higher at 4.18 × 10^13^ M^–1^ s^–1^, exceeding the diffusion–controlled limit (Figure S31). This implies that the interaction between Fl and complex **1** occurs in the ground state. Further titration experiments monitored by ultraviolet–visible (UV–vis) spectroscopy revealed that a complex formed between **1** and Fl as the concentration of **1** in the solution increased (Figure S32). No change in UV–vis spectra in the presence of TEOA was observed (Figure S33). In addition, the high concentration of **1** in the photocatalytic system drastically reduced the TOF value (Table S13). These observations indicate that Fl undergoes a dynamic quenching process with TEOA, whereas the interaction with **1** results in static non–fluorescence quenching. Moreover, in our photocatalytic system, the TEOA concentration is at least 4 orders of magnitude higher than that of complex **1**. These results suggest that the reductive quenching pathway is dominant (Figure 5). First, the photosensitizer Fl undergoes reductive quenching, where its excited state is quenched by TEOA to form the reduced Fl^–^ species. This Fl^–^ species subsequently reduces the Ni complex, facilitating the hydrogen evolution reaction. The reduction potential of Fl/Fl^–^ species was reported to be −1.53 V,^45^ which is more negative than the Ni(II/I) redox potentials of **1**, but comparable to that of the Ni(I/0) couple in **1**.

To determine whether the HER mechanism mediated by complex **1** follows an ECEC or EECC pathway, electrochemical experiments were performed. Electrocatalytic voltammograms of **1** in the presence of acetic acid revealed the onset of electrocatalytic current around −1.5 V (Figure S34) compared to the redox couple of Ni(II)/Ni(I) at −0.84 V and Ni(I)/Ni(0) at −1.53 V. Combined with the observation of 91% Faraday efficiency in bulk electrolysis experiments at −2.1 V (vs Fc/Fc^+^), it indicates HER reaction catalyzed by **1** proceeds via an EECC mechanism. The low driving force between Fl/Fl^–^ and the Ni(I/0) redox couple from **1** could account for the low turnover frequency observed in the photocatalytic system. Based on these mechanistic studies, the overall photocatalytic reaction mechanism is summarized in Figure 5.

To evaluate the stability of the Ni(II) catalysts in an alcohol-containing aqueous solution, the ultraviolet–visible–near infrared (UV–vis–NIR) spectrum of complex **1** was recorded in a mixed solvent of CH_3_CN/CH_3_OH/H_2_O in a 1:2:2 ratio (with CH_3_CN added to ensure solubility). The spectral pattern remained unchanged compared to that recorded in pure CH_3_CN (Figure S35). To elucidate the catalytic intermediates in Ni(I) and Ni(0) states, the chemical reduction of complex **1** was employed. The reaction with one equivalent of decamethylcobaltocene (CoCp*_2_) caused a clear shift in the UV–vis–NIR spectrum, which showed that the Ni(I) state was accessible (Figure S36). However, the spectral change in the presence of two equivalents was less pronounced. In situ UV–vis–NIR spectroelectrochemical measurements further facilitated the identification of the Ni(I) state. At a potential of −1.05 V (vs Fc/Fc^+^), the feature at 400–500 nm increased within 10 s, indicating a Ni center oxidation state change (Figure S37). The spectral change showed minimal deviation from the setup at −1.05 V when the potential was set at −1.77 V (vs Fc/Fc^+^), consistent with observations using chemical reduction. The X-band electron paramagnetic resonance (EPR) spectrum of complex **1**, after applying a potential of −1.3 V (vs Fc/Fc^+^) for 0.5 h, exhibits a *g* ≈ 2 signal, consistent with the formation of a Ni(I) species. In contrast, the solution subjected to a potential of −1.9 V (vs Fc/Fc^+^) is EPR-silent, likely indicating the generation of a Ni(0) state (Figure S38). To further investigate a Ni(0) state within this ligand system, bis(1,5–cyclooctadiene)nickel(0) (Ni(COD)_2_) was reacted with two equivalents of the PN1 ligand in CD_3_CN. The resulting brown solution was EPR silent (Figure S39) and exhibited a relatively sharp ^31^P NMR signal, suggesting the formation of a diamagnetic species (Figure S40). Also, the resonance at 27.4 ppm, which is less shielded than the free ligand’s resonance at −9.6 ppm,^31^ shows that the ligand binds to the Ni center. The ^31^P NMR peak is more shielded than that of **1** (44.4 ppm), consistent with a lower oxidation state than Ni(II) in **1**. Collectively, these results suggest that both Ni(I) and Ni(0) states are likely accessible within this ligand system.

DFT calculations (BP86/def2–TZVP//BP86/def2–SVP, see the SI) were conducted to examine the *d*-orbital occupancies of [Ni(II)]^2+^, [Ni(I)]^+^, and [Ni(0)]^0^. It is evident that during both reduction processes, electrons are sequentially added to the vacant d_*x*^2^–*y*^2^_ orbital, which is the lowest unoccupied molecular orbital (LUMO) of [Ni(II)]^2+^ (Figure S41). When [Ni(II)]^2+^ is reduced to [Ni(I)]^+^ through a single electron transfer, the Ni center retains its square planar structure. However, an additional electron transfers to [Ni(0)]^0^ leads to a double occupancy of the d_*x*^2^–*y*^2^_ orbital, inducing a shift from the square–planar to a tetrahedral configuration.

DFT calculations were also performed to provide further insights into the mechanism of HERs catalyzed by **1** and **2**. Due to its paramagnetism, discussions of DFT calculations for **3** are included (Scheme S1). Based on our experiments, the initial step involves the one–electron reduction of the Ni complexes from Ni(II) to Ni(I). Our DFT calculations predicted a reduction potential of −0.89 V vs Fc^+^/Fc for **1** in acetonitrile, which aligns with our experimental measurement of −0.84 V vs Fc^+^/Fc.

**Scheme 2:**
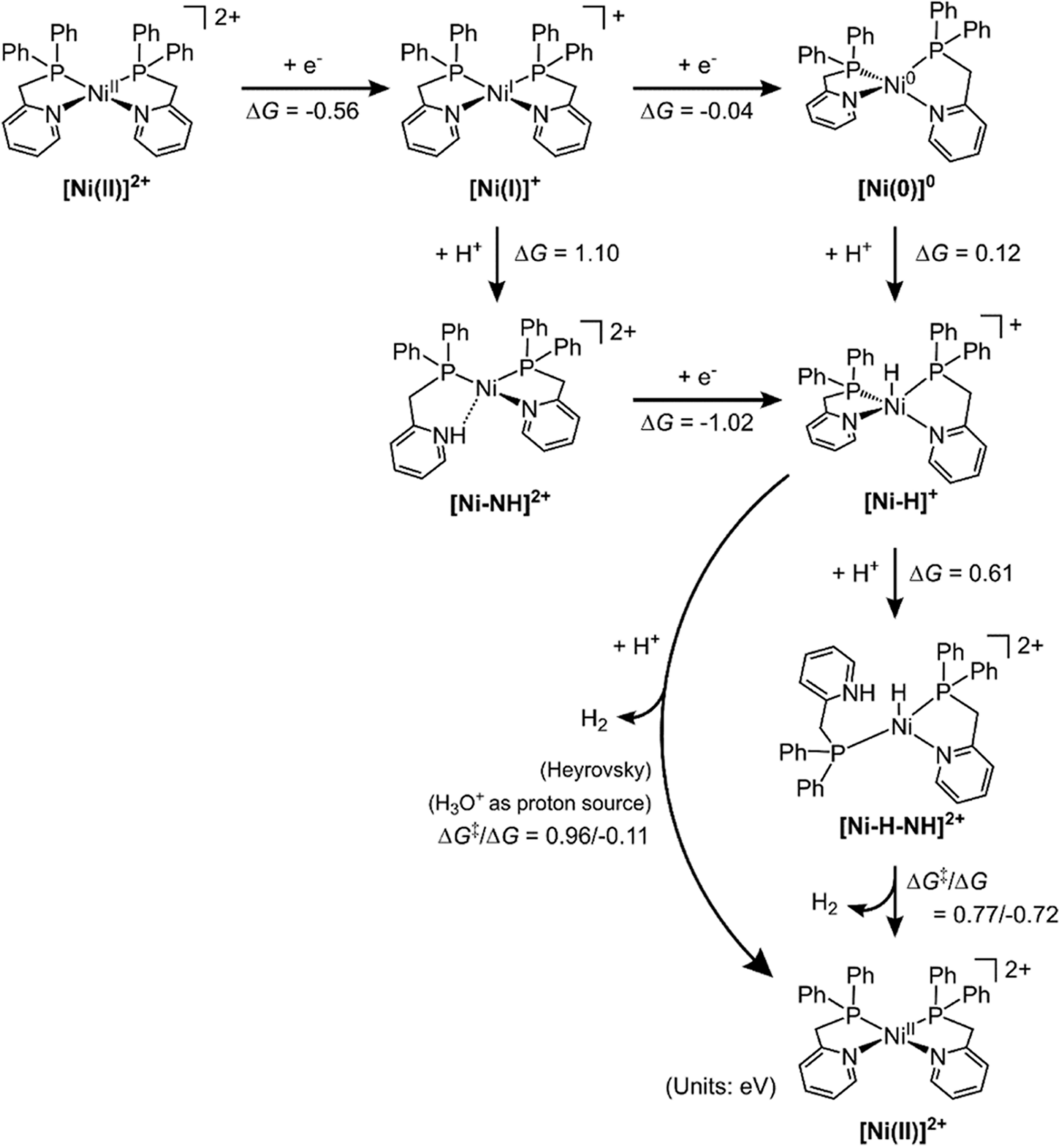
Free Energy Profile for the HER of 1 in an Aqueous Medium, Calculated at *U* = −1.53 V vs Fc^+^/Fc and pH 10.45

To align with the experimental setup for H_2_ production in the aqueous phase, we computed Gibbs free energy surfaces under the condition of the applied potential (*U*) of −1.53 V vs Fc^+^/Fc, owing to the incorporation of Fl as the PS,^45^ and a pH of 10.45. Initially, [Ni(II)]^2+^ is reduced by one electron to form [Ni(I)]^+^, exhibiting a Δ*G* value of −0.56 eV (Scheme 2). Following this, [Ni(I)]^+^ has two potential pathways: further reduction to [Ni(0)]^0^ or protonation to form [Ni–NH]^2+^. Our DFT analysis indicates a thermodynamic preference for the reduction route (Δ*G* = −0.04 eV) over the protonation (Δ*G* = 1.10 eV). This reduction route is followed by protonation on Ni to form [Ni–H]^+^ with a Δ*G* of 0.12 eV.

Beginning with the [Ni–H]^+^ complex, H_2_ can be produced through two distinct pathways. The first involves binding the second proton to the nitrogen of the dissociated pyridine moiety, with the subsequent coupling of this proton and a hydrogen on Ni to form H_2_. However, our DFT calculations show for **1** this pathway is less likely, as adding a proton to the nitrogen of the dissociated pyridine moiety incurs a free energy cost of 0.61 eV, and the free energy barrier for the H–H coupling is 0.77 eV, leading to a total free energy barrier of 1.38 eV.

An alternative route for H_2_ formation involves the coupling of a Ni–bound hydrogen with a proton from the solvent. Utilizing H_3_O^+^ as the proton source in our calculations, we determined the free energy barrier for H_2_ formation via this mechanism is 0.96 eV. This value is notably 0.42 eV lower than the barrier of the first pathway, thereby indicating this route as the predominant pathway for H_2_ formation. However, it is important to note that this barrier, being 0.21 eV higher than the 0.75 eV kinetic threshold necessary for rapid electrochemical reactions,^46^ implies that H_2_ formation is a slow process. This result aligns with what we have observed experimentally.

Complex **2** follows the same reaction pathway and has a kinetic barrier (0.95 eV, Scheme S2) for H–H coupling similar to that of complex **1**. This result is also consistent with our experimental observation that complexes **1** and **2** exhibit similar reactivity toward H_2_ formation in the early stage of the catalysis. We also computed the reaction mechanism for complex **3** and found that it follows the same pathway as complexes **1** and **2**, even though it has a pendant pyridine to host a proton (Scheme S3). Our DFT calculations reveal that the reaction free energies (Δ*G*) for the one–electron reductions of Ni(I) to Ni(0) in complex **1** and complex **3** using Fl^–^ are quite similar, with values of −0.04 and −0.07 eV, respectively (Schemes 2 and S2), differing by only 0.03 eV. This similarity aligns with the comparable HER performance observed for both complexes. Additionally, our experiments demonstrate that the reduction of Ni(I) to Ni(0) in **1** and **3** occurs at similar applied voltages. In summary, our DFT results support an EECC reaction pathway, encompassing two-electron reduction followed by two chemical processes. The mechanism is different from ECEC or CECE mechanisms; the former is proposed for Ni bis(diphosphine) systems (Scheme 1a)^47^ and Ni(II) bis(chelate) catalysts (Scheme 1b–d).^16^ The latter is suggested for Ni pyridylthiolate catalysts (Scheme 1e,f).^24^

Finally, we attempt to use DFT calculations to rationalize how the substituents on the phosphinopyridine ligand influence the TON of hydrogen production. The stability of photosensitizers and metal catalysts is crucial for the durability of a hydrogen evolution photocatalytic system. Photosensitizers (e.g., fluorescein) may degrade during reductive quenching, and photocatalysts can lose activity due to ligand dissociation, exchange, or hydrogenation.^24,49,50^ Since the same photosensitizer is used, the difference in the TON is likely to relate to the stability of the homogeneous catalysts, which could be deactivated due to the dissociation of the supporting ligands.

We thus compared the ligand dissociation energies (Δ*G*_diss_) for **1**, **2**, and **3** in their [Ni(II)]^2+^, [Ni(I)]^+^, and [Ni(0)]^0^ states. It was observed that Δ*G*_diss_ is notably smaller at the [Ni(I)]^+^ state compared to the other two states, indicating a higher likelihood of phosphinopyridine ligand dissociation in this state (Figure S42). Importantly, at the [Ni(I)]^+^ state, **1** shows a larger Δ*G*_diss_ than **2** (1.02 vs 0.82 eV), suggesting greater stability of **1** against ligand dissociation. This aligns with the higher TON observed for **1** compared to **2**. Additionally, when comparing Δ*G*_diss_ between **1** and **3** at the [Ni(I)]^+^ state, **1** also exhibits a slightly higher Δ*G*_diss_ than **3** (1.02 vs 1.01 eV). This indicates that **1** is marginally more stable against ligand dissociation than **3**, which correlates with the slightly higher TON for **1** as compared to **3**.

## Conclusions

A series of Ni(II) complexes featuring phosphinopyridyl ligands has been successfully synthesized and thoroughly characterized in both solid and solution states. While complexes **1**, **2**, and **4** exhibit square–planar Ni(II) centers in both states, complex **3**, displaying paramagnetic behavior, reveals a six–coordinate Ni(II) center by interacting with pendant pyridyl groups axially. Electrochemical analyses reveal two distinct redox couples during the reduction process, indicating accessibility to Ni(I) and Ni(0) states. Incorporating 6–methyl substituents into the pyridine donors of the ligands is hypothesized to facilitate access to lower metal oxidation states, as evidenced by a significant increase in the redox potential of complexes **2** and **4** compared to complexes **1** and **3**, respectively. However, this modification may compromise the stability of Ni(I) states, as indicated by DFT calculations, potentially affecting catalytic durability. Nonetheless, these complexes demonstrate robust performance in catalytic systems, yielding high TONs surpassing those reported for first–row transition–metal molecular catalysts utilizing organic or inorganic dyes as PSs. Photophysical investigations suggest that the reductive quenching pathway is dominant. Electrochemical studies, combined with DFT calculations, propose an EECC mechanism involving two electrons added to the *d*_*x*^2^–*y*^2^_ orbital in the initial reaction stage, followed by two protonation processes. The exceptional catalytic stability of these complexes can be attributed to their unique ligand system, which comprises both phosphine and pyridine donors with π–back–donating properties. This characteristic likely stabilizes Ni centers in lower oxidation states (+1 and 0) and catalytic intermediates, thereby enhancing catalyst durability. In addition, a square–planar nickel center within the primary coordination sphere, with axially located labile binding sites, is essential for substrate accessibility. Furthermore, when the oxidation state changes from Ni(II) to Ni(0), bis(chelate) coordination allows for a geometric transformation from square-planar to tetrahedron. This study offers valuable insights for the development of robust catalysts for homogeneous hydrogen evolution.

## Methods

### Materials and General Methods

All experimental procedures were conducted under a nitrogen atmosphere using standard Schlenk techniques or within a glovebox under dinitrogen. Solvents were dried and distilled according to established protocols. All starting materials were obtained from commercial suppliers and used without further purification. The ligands PN1, PN1^Me^ and PN2 were synthesized following previously reported methods.^30−35^

### Spectroscopic, Electrochemical, and Structural Characterization of Complexes

Electronic absorption spectra were recorded using a Hewlett-Packard 8453 spectrophotometer. NMR spectra were acquired on Bruker AVNEO 500 spectrometers. The ^1^H NMR spectra were referenced to the residual proton signals of the solvents, and the ^31^P NMR spectra were referenced to the external standard O=P(OPh)_3_ at −17.7 ppm. The magnetic susceptibility of [**3**][(ClO_4_)_2_] was measured by Evans method using ^*t*^BuOH as an internal standard.^51^ Elemental analyses were performed on an Elementar Vario EL III instrument. Electrochemical measurements were conducted using a CHI Model 421 electroanalyzer, with complexes dissolved in an CH_3_CN solution (0.1 mM) containing NBu_4_PF_6_ (0.1 M) as the supporting electrolyte. A platinum working electrode and Ag/AgNO_3_ reference electrode were employed, and potentials were referenced to the ferrocenium/ferrocene (Fc^+^/Fc) couple using ferrocene (Fc) as an external standard. Differential pulse voltammetry measurements were obtained using a Metrohm Autolab PGSTAT IMP system. X-ray crystallographic data were collected on a Bruker D8 Venture 3.0 diffractometer with Mo Kα radiation and a CCD detector. Least-squares refinement of positional and anisotropic thermal parameters was performed for all non-hydrogen atoms, while hydrogen atoms were placed in fixed positions. The refinements were based on F^2^ data, and an SADABS absorption correction was applied.^52^ The SHELXTL program was used for structure refinement, with anisotropic displacement factors refined for all non-hydrogen atoms, and hydrogen atoms treated using the riding model.^53^

### Photocatalytic Hydrogen Evolution

Photocatalytic H_2_ evolution experiments were conducted in a sealed quartz cell under visible-light LED illumination (415–420 nm, 300 mW). The reaction mixture, comprising the nickel complex catalyst, photosensitizer, and sacrificial electron donor, was prepared in a hybrid solvent system (ethanol/water or methanol/water). Before irradiation, the mixture was purged with N_2_ for 5 min and degassed with an Ar/CH_4_ (9:1 v/v) mixture, using CH_4_ as an internal standard for quantitative analysis. After irradiation, the headspace gases were collected with a gastight syringe and analyzed for H_2_ content using a GC-TCD (Agilent Technologies 6890N). The amount of H_2_ generated was quantified daily, with the reaction degassed and reilluminated between measurements. Daily H_2_ production was added to the cumulative total. Turnover numbers (TONs) were calculated as the ratio of moles of H_2_ produced to moles of nickel catalyst used, over the course of the reaction.

### In Situ Spectroelectrochemical Measurement

Electrochemical measurements were recorded using an Autolab PGSTAT302N potentiostat. Spectroelectrochemical experiments were performed in a thin-layer quartz cuvette (optical path length: 1 mm) equipped with a platinum gauze as the working electrode, a platinum wire as the counter electrode, and an Ag/Ag^+^ reference electrode. UV–vis spectra were acquired using an SR2 UV–vis spectrometer (Ocean Insight).

### Calculation of Quantum Yield for Photocatalytic Hydrogen Evolution

Quantum yield calculations were performed using systems containing 4.4 μM [**1**][(ClO_4_)_2_] (22 nmol), 18.6 mM Fl (93 μmol), and 0.42 M TEOA (2.1 mmol). The light power (*P*) was measured with a PM100D power meter (Thorlabs) equipped with an S314C detector (Thorlabs). The quantum yield (Φ) was calculated after 1 day of irradiation according to reported methods.^54^

### Fluorescence Quenching Measurement

Fluorescence detection was performed under monochromatic light at 420 nm, generated using a 0.3 W blue LED light source and measured with a spectrophotometer (Hitachi F7000 or Hong-Ming Technology Co., Ltd. QME, PS060). Stern–Volmer quenching studies were conducted by measuring the emission intensity of Fl using a fluorescence spectrophotometer. Samples were prepared in sealed cuvettes and degassed with N_2_ prior to measurements. The quenching ratio  and the Stern–Volmer constant (*K*_SV_) were calculated using the equation , where *I*_f_^0^ represents the emission intensity of the photosensitizer in the absence of the quencher, and [Q] denotes the concentration of the quencher. The quenching rate constant (*k*_q_) was determined using the formula , where τ_0_ is the lifetime of the photosensitizer.

### Faradaic Efficiency Measurement

Electrochemical measurements were recorded using an Autolab PGSTAT IMP potentiostat. Bulk electrolysis was conducted in an H-cell divided into two compartments by a glass frit, each containing 10 mL of acetonitrile with 0.1 M NBu_4_PF_6_. [**1**][(ClO_4_)_2_] (1 mM) and acetic acid (100 mM) were added to both compartments. Two glassy carbon plates (1.5 × 1.0 cm^2^ submerged area) served as the working and counter electrodes. The reference electrode was a silver wire immersed in acetonitrile containing 0.01 M silver nitrate and 0.1 M NBu_4_PF_6_. Electrolysis was performed at −2.1 V (vs Fc^+^/Fc) for 5 min, with continuous stirring. After electrolysis, a 300 μL sample of the headspace gas was collected using a gastight syringe. The H_2_ concentration was analyzed by a Shimadzu Nexis GC-2010 gas chromatograph, equipped with a Micropacked ST column (2.0 m × 1.0 mm ID) and using helium (99.9999% purity) as the carrier gas at a flow rate of 300 kPa. A barrier discharge ionization detector was used for product analysis

### EPR Measurement Following Bulk Electrolysis

Bulk electrolysis was conducted in an H-cell divided into two compartments by a glass frit. Each compartment contained 10 mL of acetonitrile with 0.1 M NBu_4_PF_6_, and [**1**][(ClO_4_)_2_] (1.5 mM) was added to both compartments. Two glassy carbon plates (1.5 × 1.0 cm^2^ submerged area) were used as the working and counter electrodes. The reference electrode was a silver wire immersed in acetonitrile containing 0.01 M silver nitrate and 0.1 M NBu_4_PF_6_. Electrolysis was performed at −1.3 and −1.9 V (vs Fc^+^/Fc) for 30 min, with continuous stirring. Postelectrolysis, the solutions were collected for EPR measurements using the BEMXnano BENCH-TOP system

### Synthesis

#### Bis[(6–methyl–2–pyridyl)methyl]phenylphosphine (PN2^Me^)

2,6-Lutidine (8.7 mL, 75 mmol) was dissolved in 100 mL of THF and cooled to −78 °C. *n*BuLi (75 mmol, 1.6 M in hexane) was added dropwise over the course of 1 h with continuous stirring. Trimethylsilyl chloride (8.2 g, 75 mmol) was then added dropwise to the solution at −78 °C. The reaction mixture was allowed to warm to room temperature and stirred overnight. The solvent was removed under vacuum, and the residue was extracted with pentane. The solid was removed by filtration. After evaporating the pentane under vacuum, the oily residue was distilled to afford 6-methyl-2-[(trimethylsilyl)methyl]pyridine. This product (12.2 g, 68 mmol) was dissolved in 100 mL of THF, and PPhCl_2_ (5.2 mL, 34 mmol) was added at −78 °C. The mixture was stirred overnight, and the THF was removed under vacuum to yield the final product. Yield: 95%. ^1^H NMR (500 MHz, CD_3_CN): δ ABX spin system (A = B = H, X = P) 2.40 (6H, s, methyl H), 3.26 (2H, d, ^1^*J*_AB_ = 13.31 Hz), 3.27 (2H, dd, ^1^*J*_AB_ = 13.31 Hz, ^2^*J*_XB_ = 1.48 Hz), 6.84 (2H, d, ^2^*J* = 7.70 Hz, pyridyl H), 6.95 (2H, d, ^2^*J* = 7.65 Hz, pyridyl H), 7.31–7.34 (3H, m, aromatic H), 7.40 (2H, t, ^2^*J* = 7.70 Hz, pyridyl H), 7.47–7.50 (2H, m, aromatic H); ^13^C{^1^H} NMR (125.8 MHz, CD_3_CN): δ 23.46 (s, *C*H_3_ of pyridyl), 37.23 (d, ^1^*J*_PC_ = 18.20 Hz, P–*C*H_2_), 120.16 (s, *C*H of pyridyl), 120.42 (d, ^3^*J*_PC_ = 4.91 Hz, *C*H of pyridyl), 128.15 (d, ^3^*J*_PC_ = 6.83 Hz, *m*–*C*H of phenyl), 129.05 (s, *p*–*C*H of phenyl), 132.78 (d, ^2^*J*_PC_ = 20.04 Hz, *o*–CH of phenyl), 136.37 (s, CH of pyridyl), 137.54 (d, ^1^*J*_PC_ = 19.35 Hz, P–*C* of phenyl), 157.66 (s, *C*H of pyridyl), 157.74 (d, ^2^*J*_PC_ = 5.87 Hz, N–*C*–CH_2_ of pyridyl); ^31^P{^1^H} NMR (202.5 MHz, CD_3_CN): δ – 13.9.

#### [Ni(PN1)_2_][(ClO_4_)_2_] ([**1**][(ClO_4_)_2_])

PN1 (0.100 g, 0.36 mmol) and Ni(ClO_4_)_2_·6H_2_O (0.066 g, 0.18 mmol) were dissolved in CH_3_CN, forming a brown solution. The reaction mixture was allowed to stand at room temperature and was subsequently layered with ether. After 2 to 4 days, brown crystalline solids of [**1**][(ClO_4_)_2_] were obtained. Yield: 66%. Anal. Calcd for C_36_H_32_Cl_2_N_2_NiO_8_P_2_: C, 53.24; N, 3.45; H,3.97. Found: C, 53.73; N, 3.44; H, 4.14. ^1^H NMR (500 MHz, CD_3_CN, 233 K): δ 3.93 (2H, s, CH_2_), 5.44 (2H, s, CH_2_), 7.12 (4H, s, *o*–H of phenyl), 7.30 (4H, s, *m*–H of phenyl), 7.38 (4H, s, *m*–H of phenyl), 7.42 (2H, s, H of pyridyl), 7.56 (4H, s, *p*–H of phenyl), 7.64 (6H, s, *o*–H of phenyl and H of pyridyl), 8.03 (2H, t, ^1^*J* = 7.3 Hz, H of pyridyl), 8.17 (2H, s, H of pyridyl); ^13^C{^1^H} NMR (125.8 MHz, CD_3_CN, 233 K): δ 41.65 (s, N–*C*–CH_2_), 122.32 (s, P–*C* of phenyl), 124.13 (s, P–*C* of phenyl), 124.54 (s, *C*H of pyridyl), 125.09 (s, *C*H of pyridyl), 129.30 (s, *m*–*C*H of phenyl), 132.96 (s, *o*–*C*H of phenyl), 133.49 (s, *p*–*C*H of phenyl), 134.31 (s, *o*–*C*H of phenyl), 141.83 (s, *C*H of pyridyl), 152.93 (s, *C*H of pyridyl), 158.29 (s, N–*C*–CH_2_); ^31^P{^1^H} NMR (202.5 MHz, CD_3_CN, 233 K): δ 44.4. UV–vis–NIR in CH_3_CN (λ, nm; ε, M^–1^ cm^–1^): 424 (4.6 × 10^2^).

#### [Ni(PN1^Me^)_2_][(ClO_4_)_2_] ([**2**][(ClO_4_)_2_])

PN1^Me^ (0.100 g, 0.34 mmol) and Ni(ClO_4_)_2_·6H_2_O (0.063 g, 0.17 mmol) were dissolved in CH_3_CN, forming a brown solution. The reaction mixture was layered with acetone and ether at room temperature. After 5 to 7 days, brown crystalline solids of [**2**][(ClO_4_)_2_] were obtained. Yield: 46%. Anal. Calcd for C_38_H_36_Cl_2_N_2_NiO_8_P_2_: C, 54.32; N, 3.33; H,4.32. Found: C, 54.73; N, 3.61; H, 4.17. ^1^H NMR (500 MHz, CD_3_CN, 233 K): δ 2.31 (6H, s, CH_3_), 4.10 (2H, s, CH_2_), 5.79 (2H, d, ^1^*J* = 16.90 Hz, CH_2_), 7.10 (4H, s, *o*–H of phenyl), 7.22 (4H, t, *m*–H of phenyl), 7.26 (2H, d, ^1^*J* = 7.83 Hz, H of pyridyl), 7.47 (2H, d, ^1^*J* = 7.76 Hz, H of pyridyl), 7.51 (6H, m, *m*–H and *p*–H of phenyl), 7.63 (2H, t, ^1^*J* = 6.73 Hz, *p*–H of phenyl), 7.86 (2H, t, ^1^*J* = 7.80 Hz, H of pyridyl), 7.94 (4H, s, *o*–H of phenyl); ^13^C{^1^H} NMR (125.8 MHz, CD_3_CN, 233 K): δ 24.61 (s, *C*H_3_), 39.92 (s, N–*C*–CH_2_), 120.81 (s, P–*C* of phenyl), 123.22 (s, *C*H of pyridyl), 123.68 (s, P–*C* of phenyl), 126.45 (s, *C*H of pyridyl), 129.45 (s, *m*– or *p*–*C*H of phenyl), 129.70 (s, *m*–*C*H of phenyl), 132.37 (s, *o*–*C*H of phenyl), 133.65 (s, *m*– or *p*–*C*H of phenyl), 134.21 (s, *p*–*C*H of phenyl), 135.14 (s, *o*–*C*H of phenyl), 142.12 (s, *C*H of pyridyl), 156.06 (s, N–*C*–CH_2_), 161.47 (s, N–*C*–CH_3_); ^31^P{^1^H} NMR (202.5 MHz, CD_3_CN, 233 K): δ 54.6. UV–vis–NIR in CH_3_CN (λ, nm; ε, M^–1^ cm^–1^): 428 (4.0 × 10^2^).

#### [Ni(PN2)_2_][(ClO_4_)_2_] ([**3**][(ClO_4_)_2_])

PN2 (0.100 g, 0.33 mmol) and Ni(ClO_4_)_2_·6H_2_O (0.063 g, 0.17 mmol) were dissolved in CH_3_CN, forming a brown solution. The reaction mixture was then layered with ether at room temperature. After 1 week, brown crystalline solids of [**3**][(ClO_4_)_2_] were obtained. Yield: 90%. Anal. Calcd for C_36_H_34_Cl_2_N_4_NiO_8_P_2_: C, 51.30; N, 6.60; H, 4.00. Found: C, 51.27; N, 6.67; H, 4.12. ^31^P{^1^H} NMR (202.5 MHz, CD_3_CN): δ 40.5. UV–vis–NIR in CH_3_CN (λ, nm; ε, M^–1^ cm^–1^): 475(3.2 × 10^2^).

#### [Ni(PN2^Me^)_2_][(ClO_4_)_2_]·2.5CH_3_CN ([**4**][(ClO_4_)_2_]·2.5CH_3_CN)

PN2^Me^ (0.100 g, 0.27 mmol) and Ni(ClO_4_)_2_·6H_2_O (0.063 g, 0.17 mmol) were dissolved in CH_3_CN, forming a brown solution. The reaction mixture was layered with ether at room temperature. After 1 week, brown crystalline solids of [**4**][(ClO_4_)_2_]·2.5CH_3_CN were obtained. Yield: 33%. Anal. Calcd for C_40_H_42_Cl_2_N_4_NiO_8_P_2_: C, 53.47; N, 6.23; H, 4.68. Found: C, 52.68; N, 6.22; H, 4.83. ^1^H NMR (500 MHz, CD_3_CN, 233 K): δ 1.64 (6H, s, CH_3_), 2.57 (6H, s, CH_3_), 2.87 (2H, m, CH_2_), 3.32 (2H, m, CH_2_), 4.01 (2H, m, CH_2_), 5.13 (2H, d, ^1^*J* = 17.62 Hz, CH_2_), 6.70 (2H, d, ^2^*J* = 7.52 Hz, H of pyridyl), 6.83 (2H, d, ^2^*J* = 7.75 Hz, H of pyridyl), 6.85 (2H, d, ^2^*J* = 7.77 Hz, H of pyridyl), 7.19 (2H, dd, ^2^*J* = 7.52 Hz, ^2^*J* = 7.77 Hz, H of pyridyl), 7.40 (2H, d, ^2^*J* = 7.73 Hz, H of pyridyl), 7.66 (4H, t, ^2^*J* = 7.68 Hz, *m*–H of phenyl), 7.70 (2H, t, ^2^*J* = 7.81 Hz, H of pyridyl), 7.82 (2H, t, ^2^*J* = 7.48 Hz, *p*–H of phenyl), 7.97 (4H, m, *o*–H of phenyl); ^13^C{^1^H} NMR (125.8 MHz, CD_3_CN, 233 K): δ 24.31 (s, *C*H_3_), 24.91 (s, *C*H_3_), 30.54 (t, *C*H_2_), 39.63 (t, *C*H_2_), 121.44 (s, *C*H of pyridyl), 122.41 (s, *C*H of pyridyl), 122.70 (s, *C*H of pyridyl), 123.63 (s, P–*C* of phenyl), 125.88 (s, *C*H of pyridyl), 129.80 (s, *m*–*C*H phenyl), 132.82 (s, *o*–*C*H of phenyl), 134.48 (s, *p*–*C*H of phenyl), 137.93 (s, *C*H of pyridyl), 141.39 (s, *C*H of pyridyl), 150.63 (s, N–*C*–CH_2_), 156.60 (s, N–*C*–CH_2_), 159.24 (s, N–*C*–CH_3_); 160.67 (s, N–*C*–CH_3_), ^31^P{^1^H} NMR (202.5 MHz, CD_3_CN, 233 K): δ 50.4. UV–vis–NIR in CH_3_CN (λ, nm; ε, M^–1^ cm^–1^): 443(3.2 × 10^2^).

### Computational Details

DFT calculations utilized the BP86^55^ functional unless otherwise specified. For all calculations, CPCM^56,57^ implicit solvation model was employed. For the calculation of redox potential from Ni(II) to Ni(I) and the HER free energy profile, acetonitrile and water were selected as solvent, respectively. Geometry optimization and vibrational frequency calculations were performed using the double−ξ quality basis set, def2–SVP.^58^ To rectify inaccuracies in vibrational entropies that arise from low–frequency modes, we have modified frequencies lower than 50 cm^–1^ to be exactly 50 cm^–1^ when performing thermal corrections.^59,60^ To attain a higher level of accuracy in electronic energy, a single point calculation was executed using the triple−ξ quality basis set, def2–TZVP.^58^ In our study, we address the common issue of overestimating molecular entropy when treating molecules as ideal gases, especially in solvent environments. To mitigate this, we have implemented the correction approach initially introduced by Wertz,^61^ which was subsequently applied in the work of Cooper and Ziegler.^62^

The Gibbs free energies were determined by the following equation
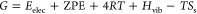
where *E*_elec_, ZPE, *H*_vib_ and denote electronic energy, zero–point energy, and enthalpy contribution from vibration, respectively. The 4*RT* term encompasses translational thermal correction, rotational thermal correction, and PV work. The temperature, *T*, was set to 298.15 K. The *S*_s_ term represents the entropy in the solvent phase.

To determine the Gibbs free energy of a proton in water, we combine the gas–phase Gibbs free energy of a proton with the experimentally measured solvation free energy of a proton in water, which is −264.0 kcal/mol.^63^ In the definition of standard hydrogen electrode (SHE)
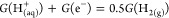
at pH = 0 and *U* = 0.0 *V*_SHE_.Thus, by calculating *G*(H_2(g)_), the *G*(e^–^) is determined to be −4.324 eV. Lastly, the impacts of pH and applied potential are accounted for using the following equation
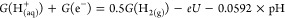
at arbitrary pH and *U*.

## Supporting Information Available

Characterization; additional experimental data and computational details (PDF)

## Abbreviations

Py_3_Me-Bpy: 6-[6-(1,1-dipyridin-2-yl-ethyl)-pyridin-2-ylmethyl]-[2,2′]bipyridinyl
PN1: (2-pyridyl)methyldiphenylphosphine
PN1^Me^: [(6-methyl-2-pyridyl)methyl]diphenylphosphine
PN2: bis[(2-pyridyl)methyl]phenylphosphine
PN2^Me^: bis[(6-methyl-2- pyridyl)methyl]phenylphosphine
TPA: tris(2-pyridylmethyl)amine
