# Supplementary material for: Square-Planar Nickel Bis(phosphinopyridyl) Complexes for Long-Lived Photocatalytic Hydrogen Evolution
Source: JACS Au. 2024 Sep 26;4(10):3976–87. doi: 10.1021/jacsau.4c00714 (PMC11522921; doi:10.1021/jacsau.4c00714)
Supplement: Supplementary file 1 — au4c00714_si_001.pdf [file au4c00714_si_001.pdf]

Supporting Information

Square-planar Nickel Bis(phosphinopyridyl) Complexes for Long-lived Photocatalytic Hydrogen Evolution

Chien-Ting Wu,[a#] Hung-Ruei Pan,[a#] Chi-Tien Hsieh,[a] Yu-Syuan Tsai,[b] Pei-Juan Liao,[a] Shuo-Huan Chiang,[a] Che-Min Chu,[a] Wei-Kai Shao,[a] Yi-Rong Lien,[a] Yu-Wei Chen,[b] Tsung-Lun Kan ,[c] Vincent C.-C. Wang,*[b] Mu-Jeng Cheng*[a] and Hua-Fen Hsu*

[a]Department of Chemistry, National Cheng Kung University, Tainan 701, Taiwan

[b]Department of Chemistry, National Sun Yat-sen University, Kaohsiung 804, Taiwan

[c]Instrument Center of National Cheng Kung University, Tainan 701, Taiwan

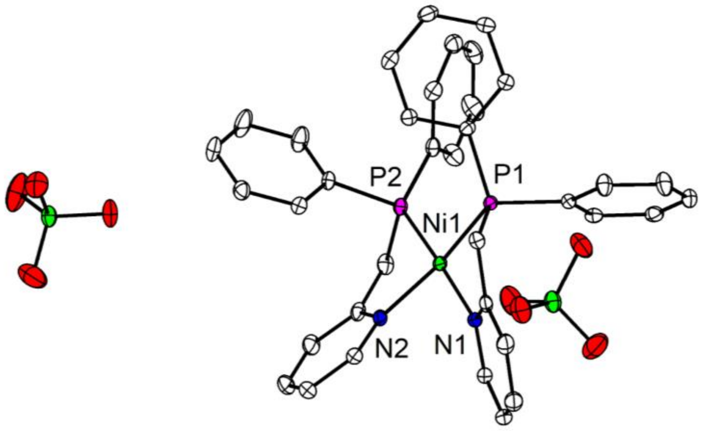

Figure S1. The ORTEP diagram of [**1**][$(ClO_4)_2$] with 35% probability. H atoms are omitted for clarity.

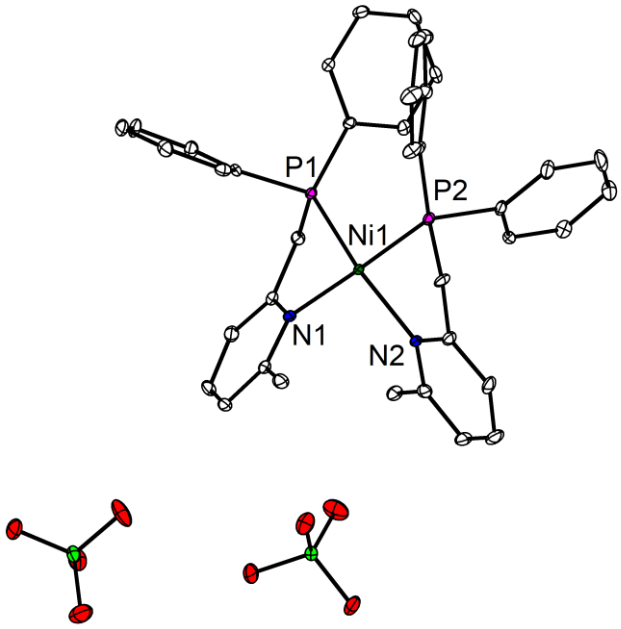

Figure S2. The ORTEP diagram of [**2**][$(ClO_4)_2$] with 35% probability. H atoms are omitted for clarity.

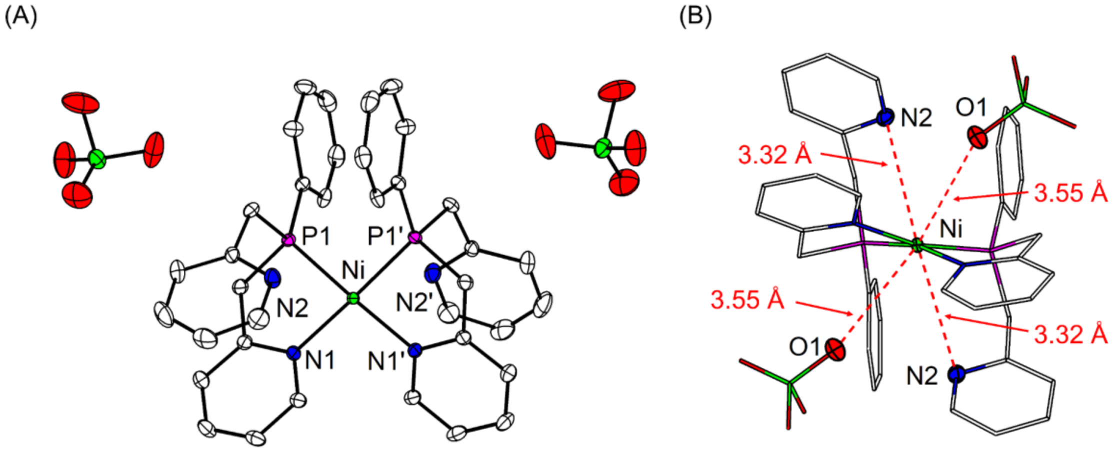

Figure S3. (A) The ORTEP diagram of [**3**][(ClO_4)$_2$] with 35% probability. H atoms are omitted for clarity. (B) Right: the figure showing the potentially weak interaction between Ni center and ClO_4^- anions or unbound pyridine donor.

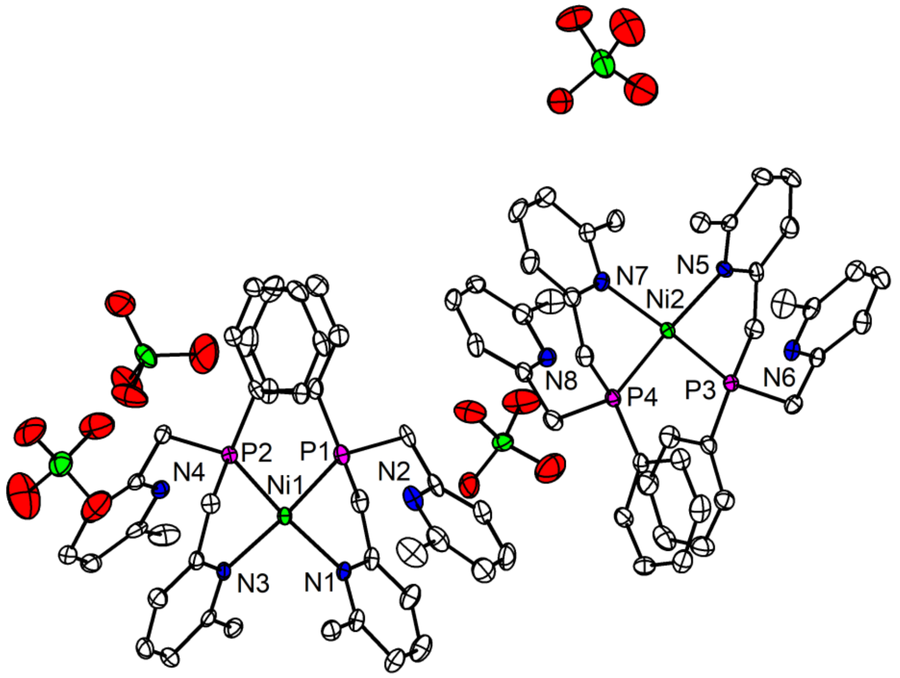

Figure S4. The ORTEP diagram of [**4**][(ClO_4)$_2$]·2.5CH_3CN with 35% probability. Each crystallographic asymmetric unit contains two independent molecules. Solvated CH_3CN molecules and H atoms are omitted for clarity.

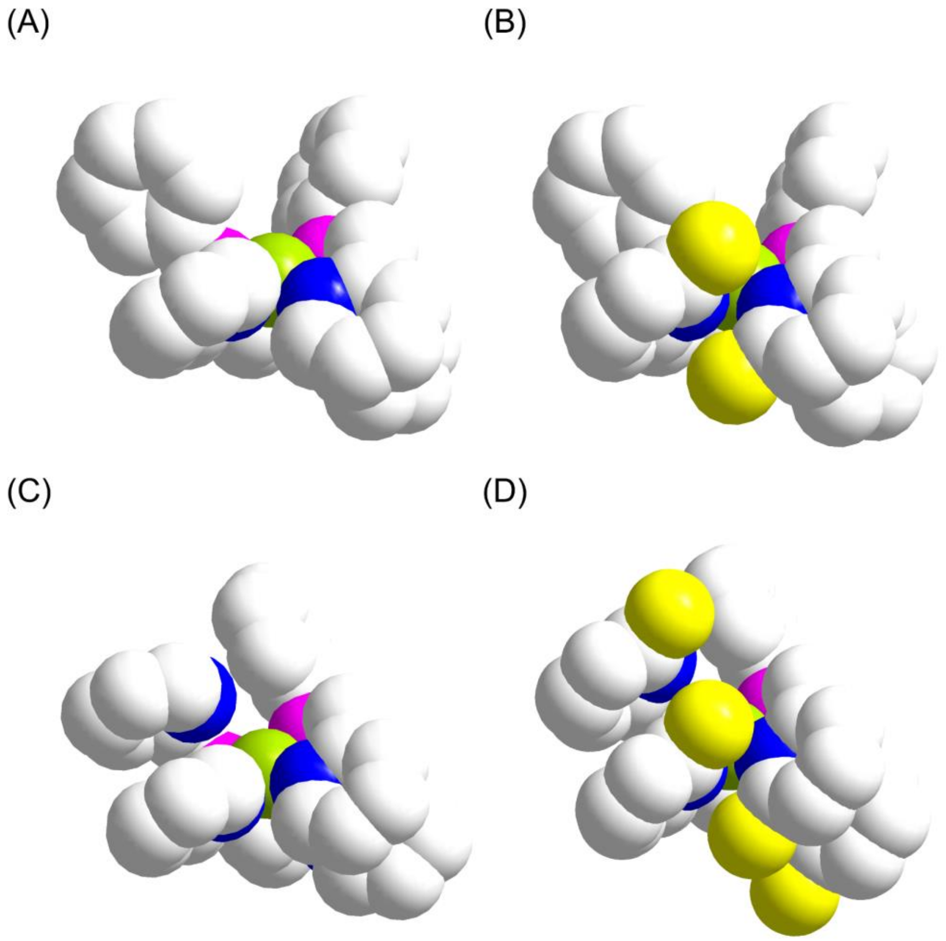

Figure S5. Spacing filling model of **1**-**4** (A-D) in side view, respectively. Green: Ni; blue: N; purple: P; white: C; yellow: methyl groups of **2** and **4**.

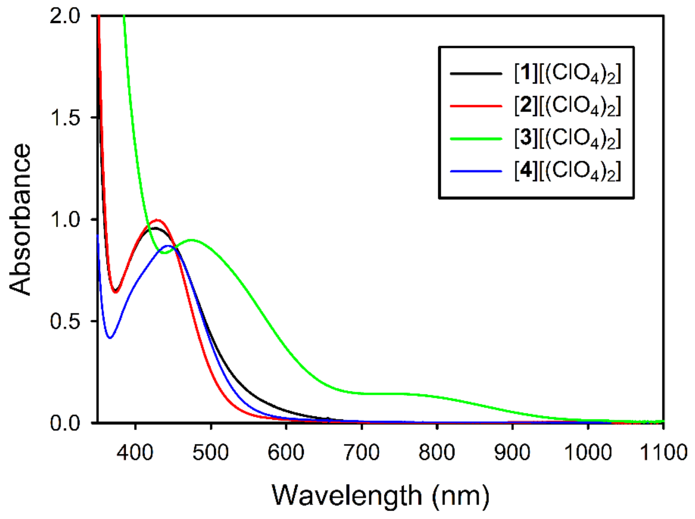

Figure S6. UV-vis NIR spectra of [**1**][$(ClO_4)_2$] to [**4**][$(ClO_4)_2$] (2 mM) in CH_3CN.

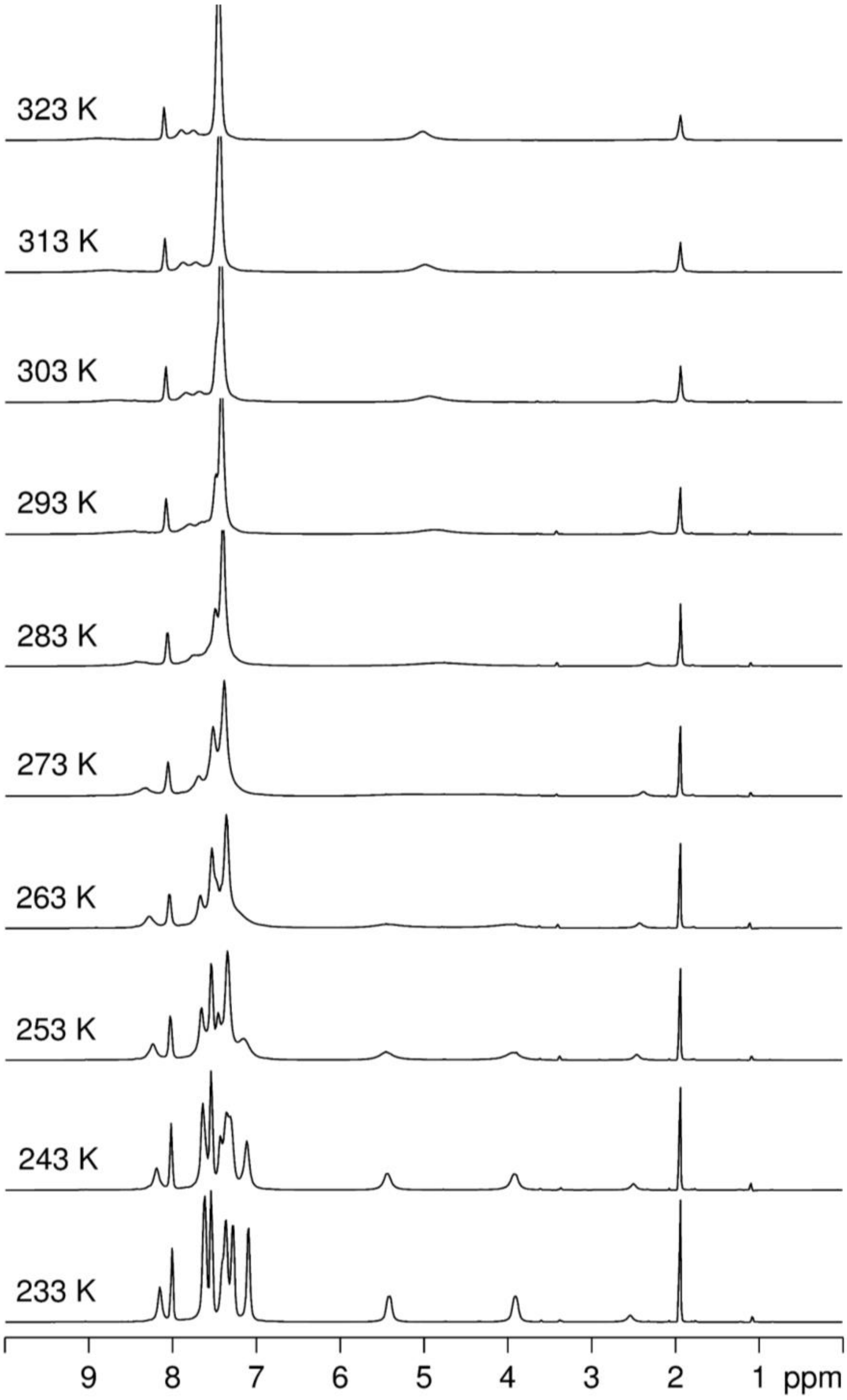

Figure S7. Variable-temperature ^{1}H NMR spectra of [**1**][(ClO_4)$_2$] in CD_3CN recorded from 233K to 323K.

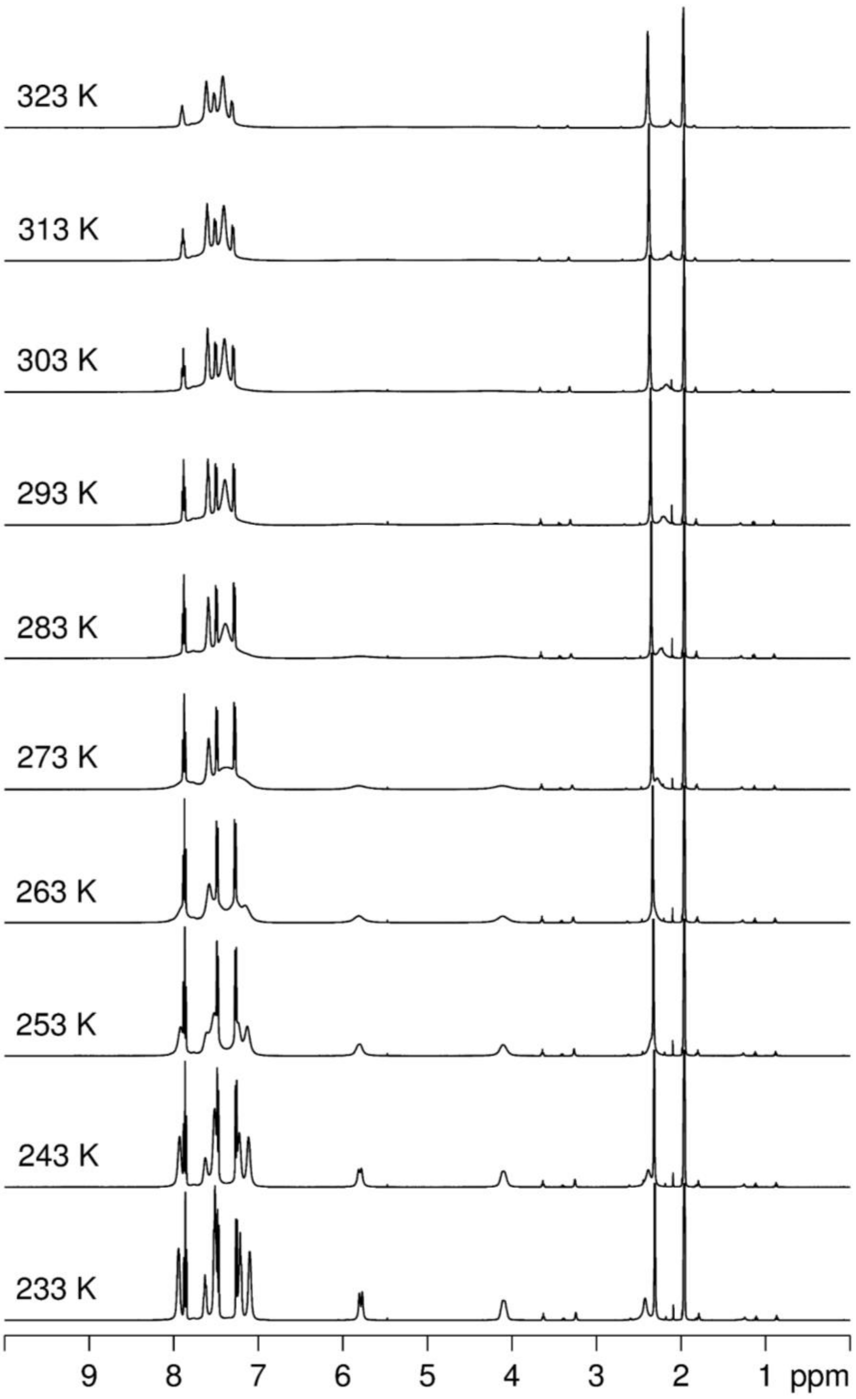

Figure S8. Variable-temperature ^{1}H NMR spectra of [**2**][$(ClO_4)_2$] in CD_3CN recorded from 233K to 323K.

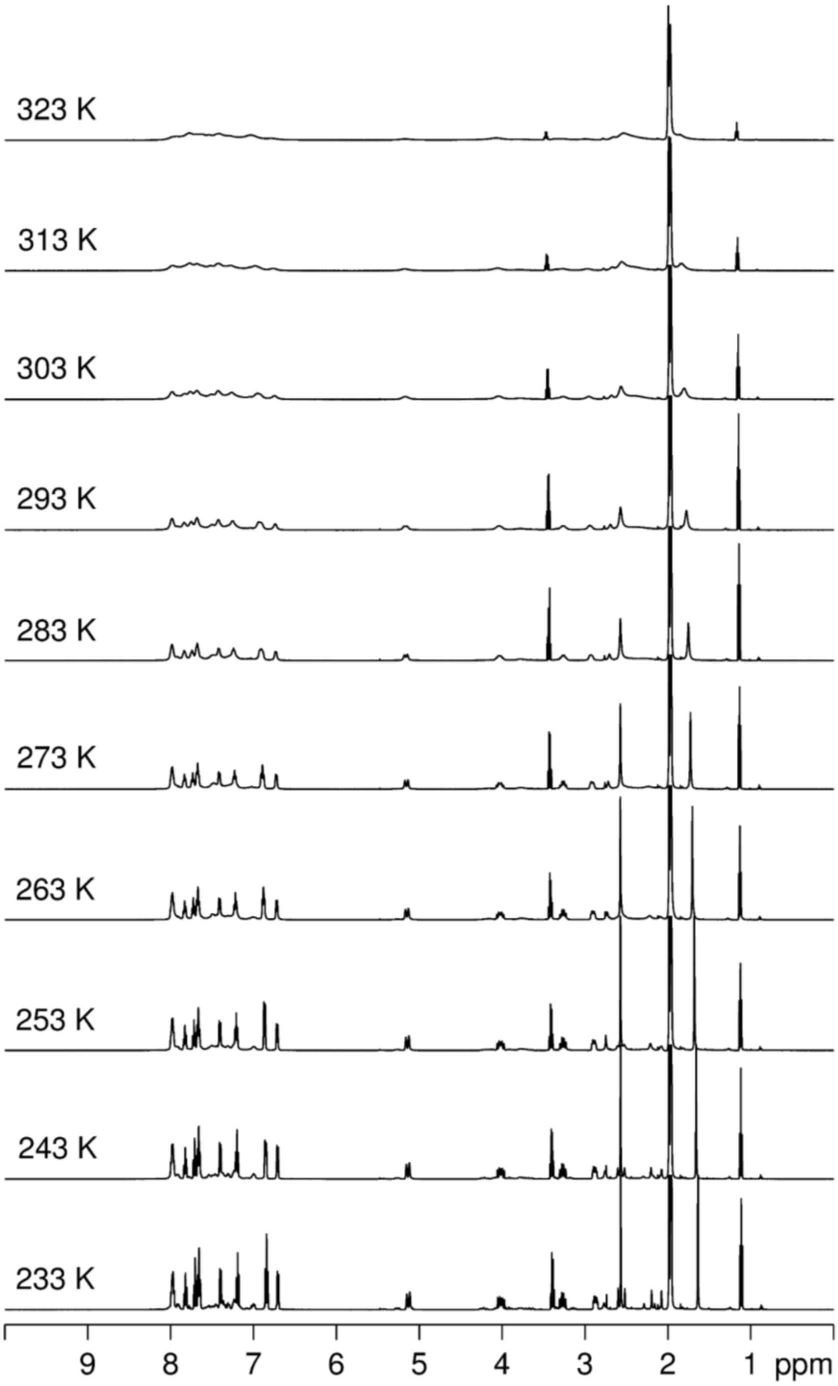

Figure S9. Variable-temperature ^{1}H NMR spectra of [**4**][$(ClO_4)_2$] in CD_3CN recorded from 233K to 323K.

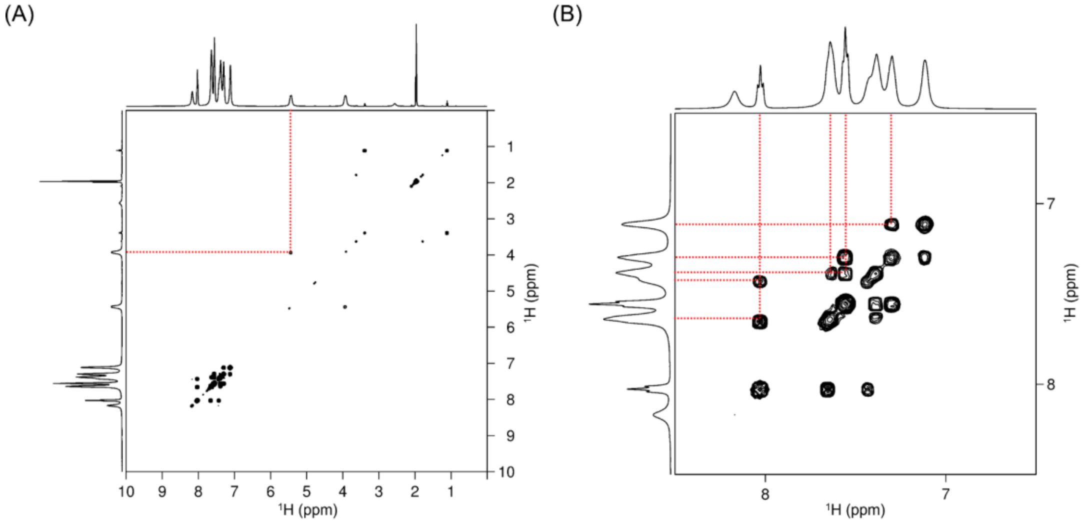

Figure S10. ^{1}H-^{1}H COSY NMR spectrum of [**1**][(ClO_4)$_2$] in CD_3CN at 233 K. (A) full range (B) expansion of aromatic region.

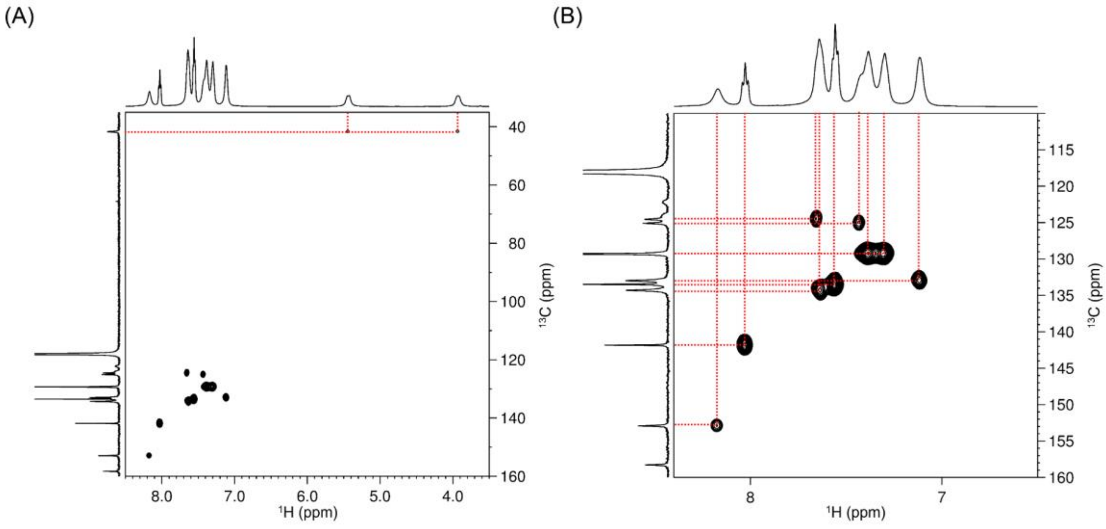

Figure S11. ^{1}H-^{13}C HSQC NMR spectrum of [**1**][(ClO_4)$_2$] in CD_3CN at 233 K. (A) full range (B) expansion of aromatic region.

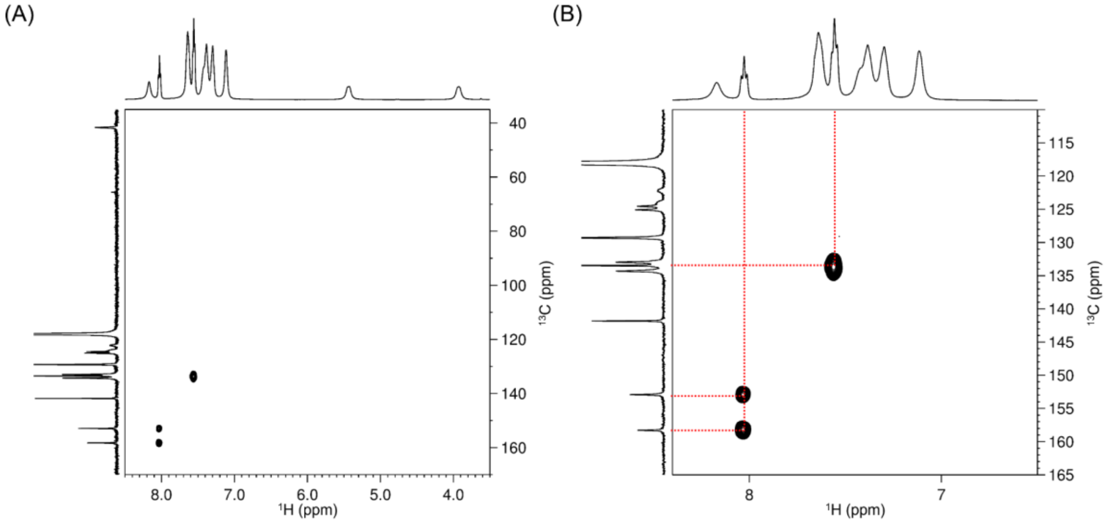

Figure S12. ^{1}H-^{13}C HMBC NMR spectrum of [**1**][(ClO_4)$_2$] in CD_3CN at 233 K. (A) full range (B) expansion of aromatic region.

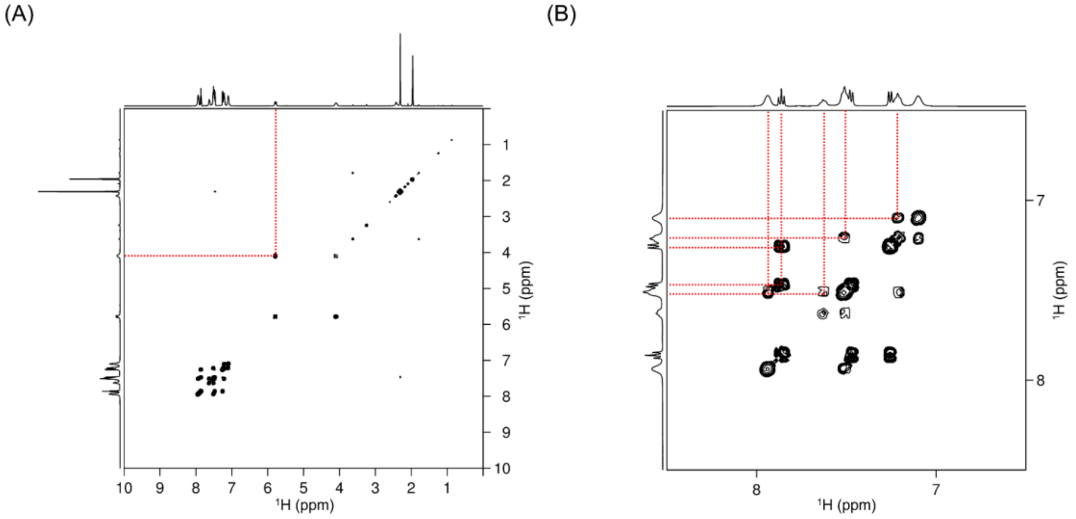

Figure S13. ^{1}H-^{1}H COSY NMR spectrum of [**2**][(ClO_4)$_2$] in CD_3CN at 233 K. (A) full range (B) expansion of aromatic region.

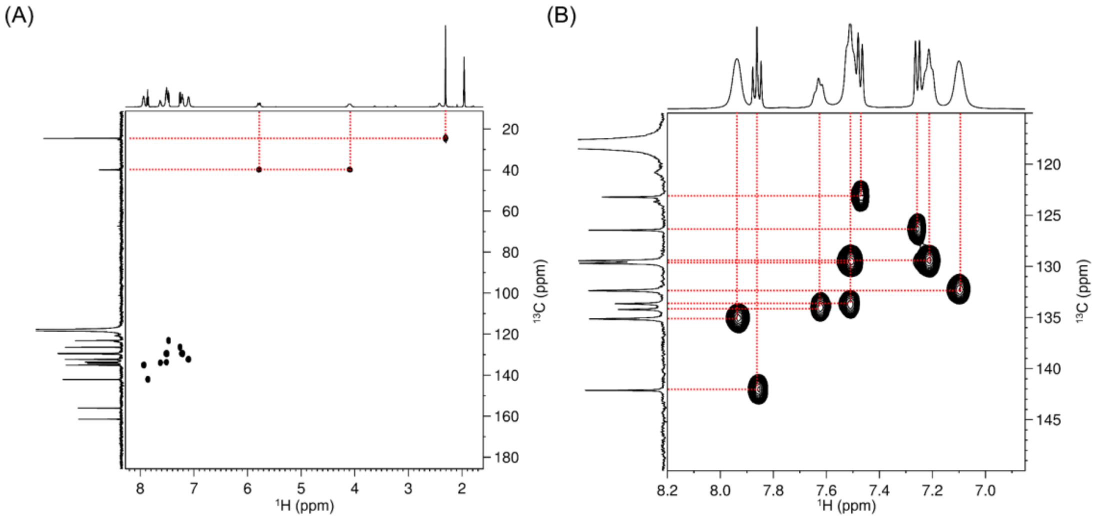

Figure S14. ^{1}H-^{13}C HSQC NMR spectrum of [**2**][(ClO_4)$_2$] in CD_3CN at 233 K. (A) full range (B) expansion of aromatic region.

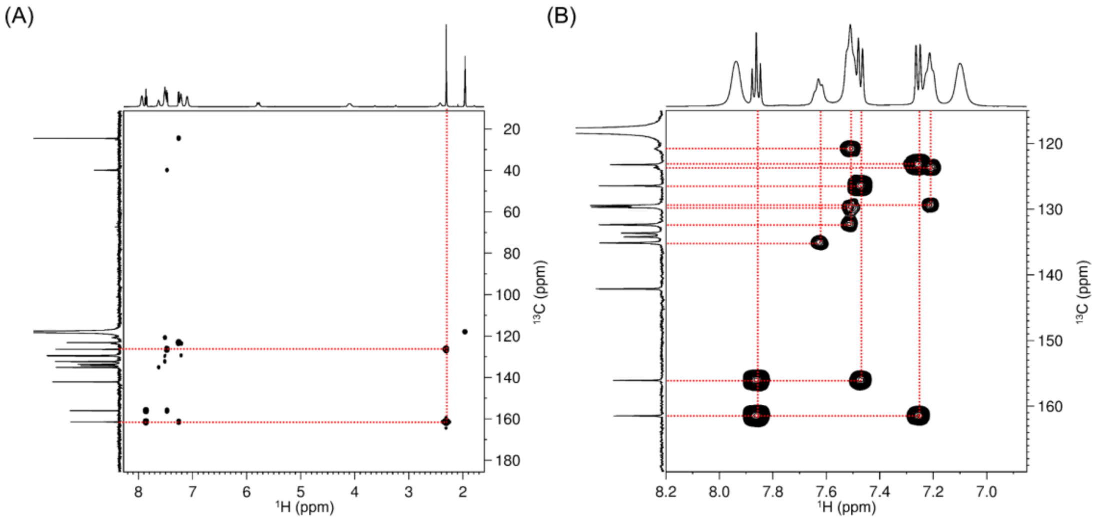

Figure S15. ^{1}H-^{13}C HMBC NMR spectrum of [**2**][(ClO_4)$_2$] in CD_3CN at 233 K. (A) full range (B) expansion of aromatic region.

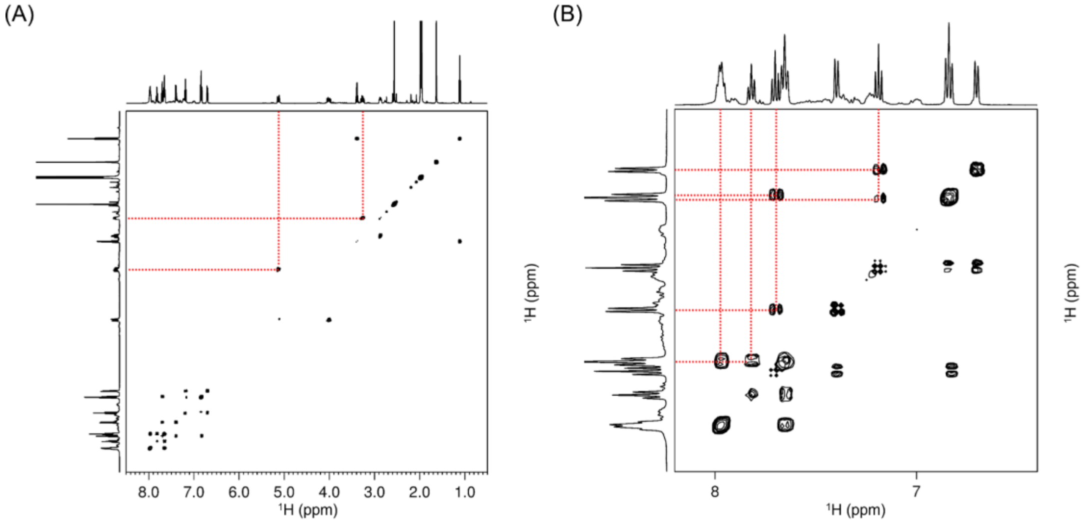

Figure S16. ^{1}H-^{1}H COSY NMR spectrum of [**4**][$(ClO_4)_2$] in CD_3CN at 233 K. (A) full range (B) expansion of aromatic region.

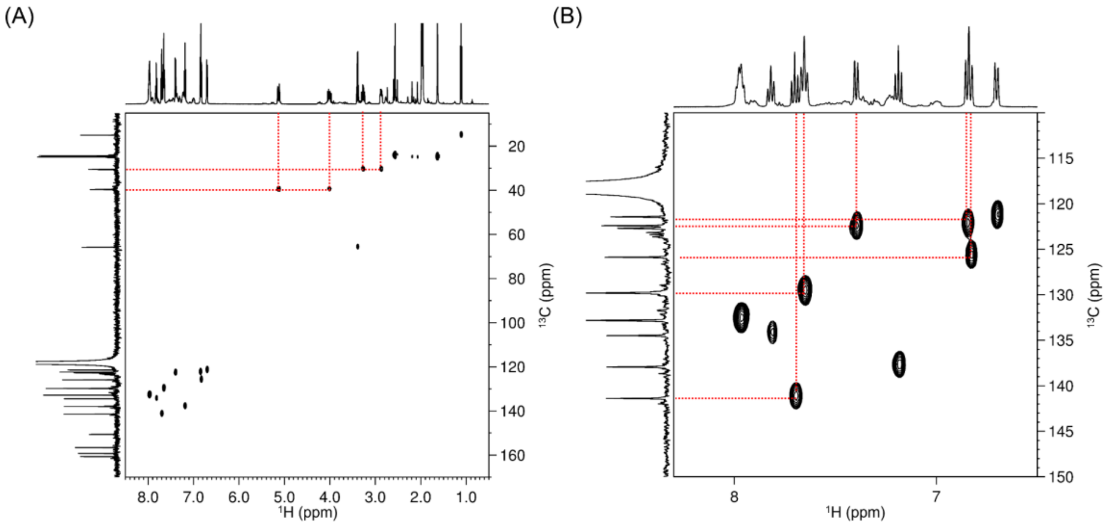

Figure S17. ^{1}H-^{13}C HSQC NMR spectrum of [**4**][$(ClO_4)_2$] in CD_3CN at 233 K. (A) full range (B) expansion of aromatic region.

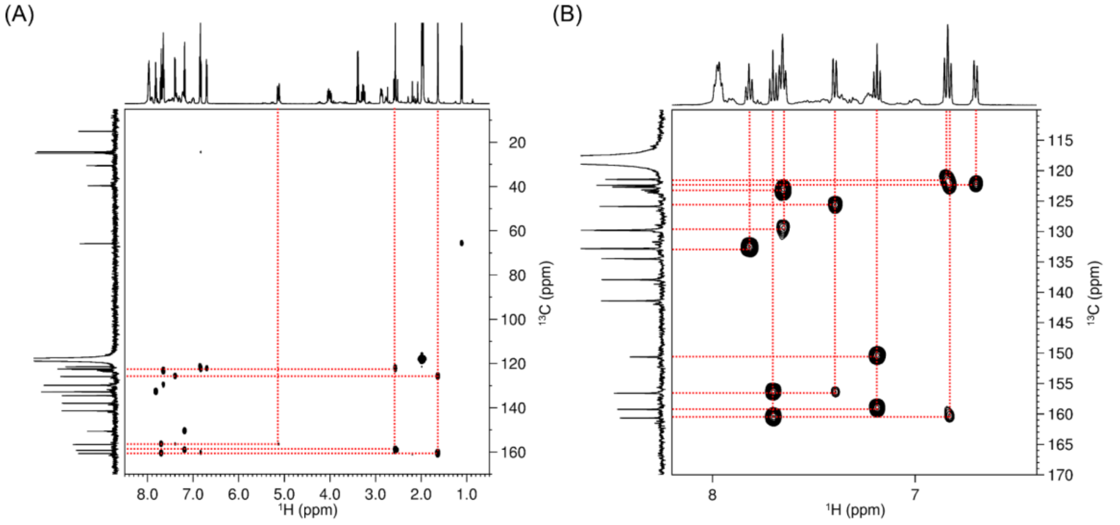

Figure S18. ^{1}H-^{13}C HMBC NMR spectrum of [**4**][(ClO_4)$_2$] in CD_3CN at 233 K. (A) full range (B) expansion of aromatic region.

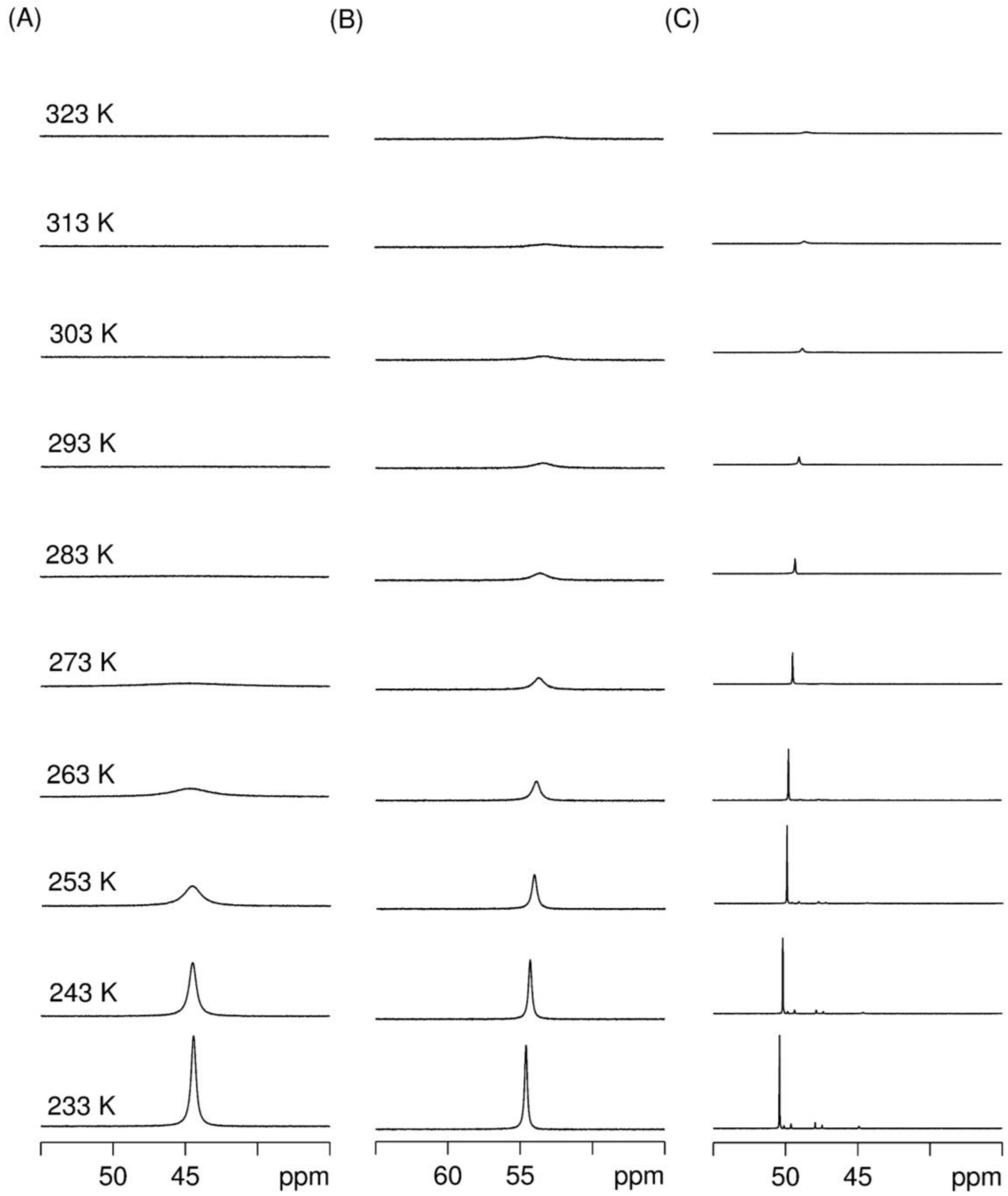

Figure S19. Variable-temperature ^{31}P NMR spectra of (A) [**1**][(ClO_4)$_2$], (B) [**2**][(ClO_4)$_2$] and (C) [**4**][(ClO_4)$_2$] in CD_3CN recorded from 233K to 323K. (note: The minor peaks observed in the spectrum of complex **4** are likely due to the presence of a small amount of the trans-isomer.)

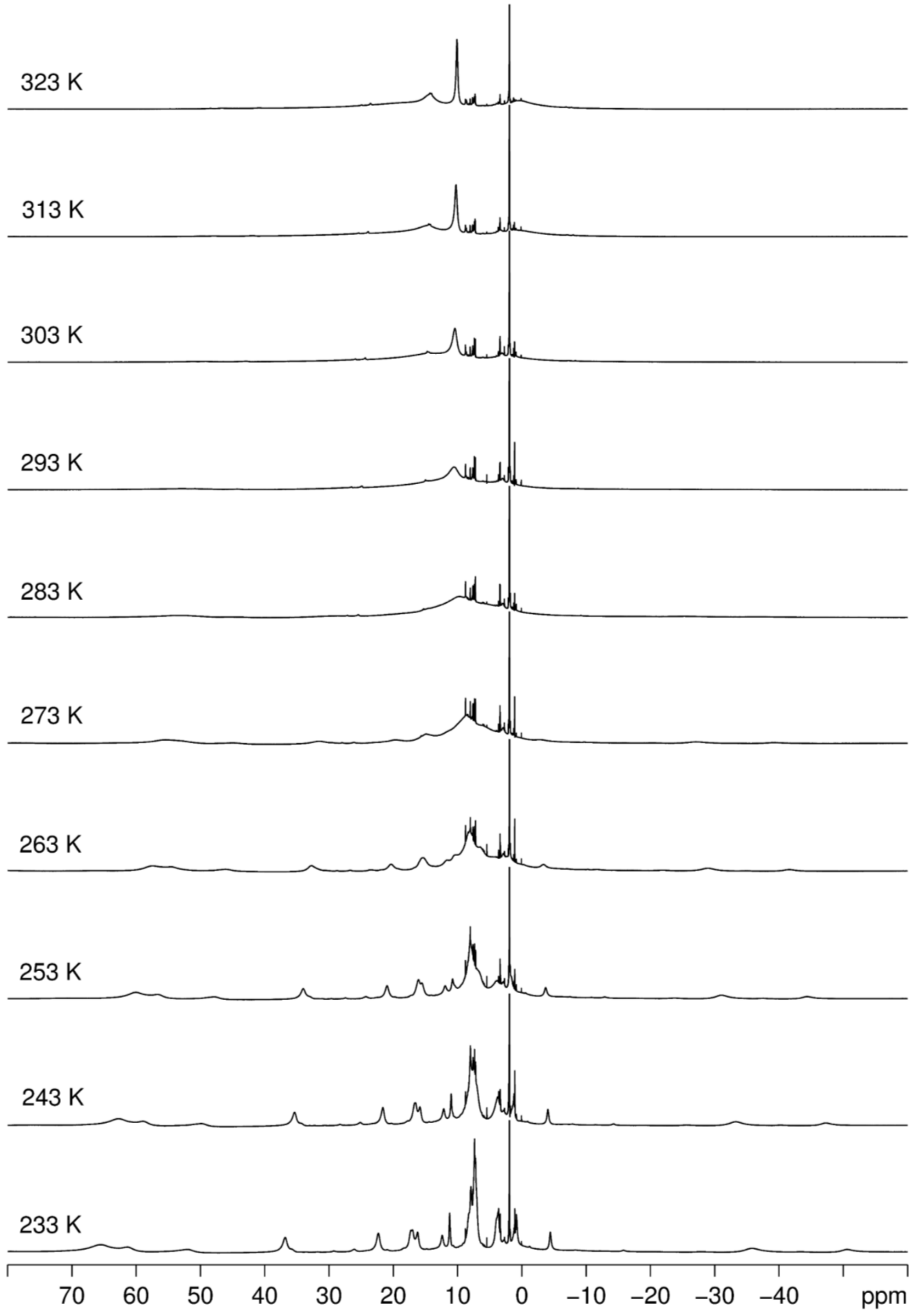

Figure S20. Variable-temperature ^{1}H NMR spectra of [**3**][(ClO_4)$_2$] in CD_3CN recorded from 233K to 323K. 80 to -60 ppm.

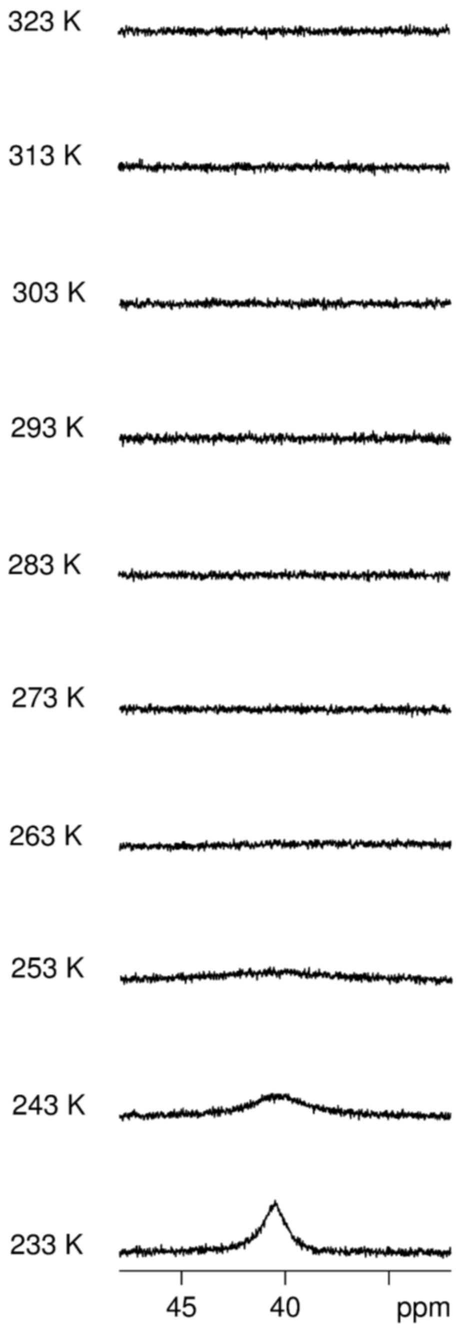

Figure S21. Varying-temperature ^{31}P NMR spectra of [**3**][$(ClO_4)_2$] in CD_3CN recorded from 233K to 323K.

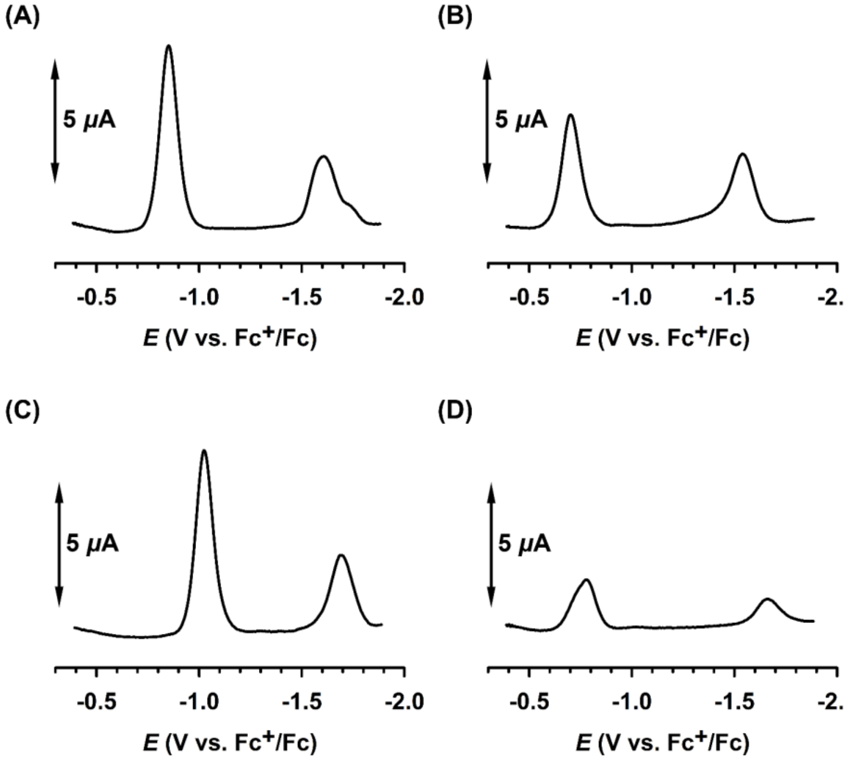

Figure S22. Differential pulse voltammetry (DPV diagrams of **1**-**4** (A-D) in CH_3CN (0.5 mM). Conditions: NBu_4PF_6, Pt, and Ag/$AgNO_3$ as the supporting electrolyte, working electrode, and reference electrode, respectively. Pulse height -0.005 V, pulse width 0.05 s, scan rate 0.01 V s^{-1}.

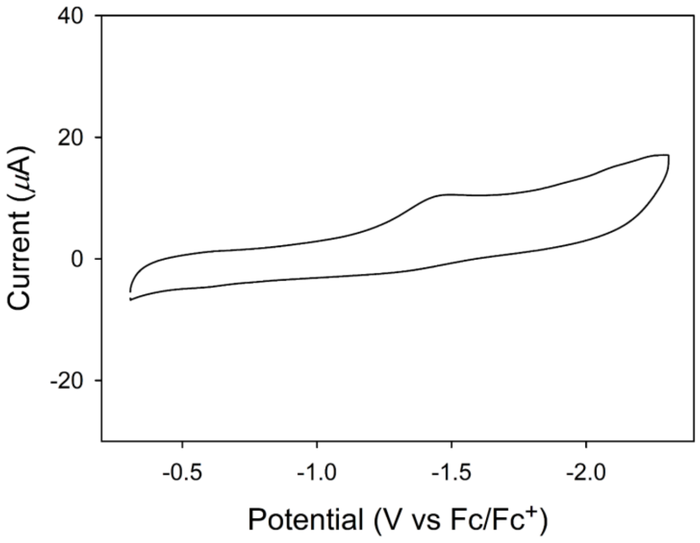

Figure S23. Cyclic voltammogram of 1 mM PN1 in CH_3CN. Conditions: 0.1 M NBu_4PF_6, Pt, and Ag/$AgNO_3$ as the supporting electrolyte, working electrode, and reference electrode, respectively.

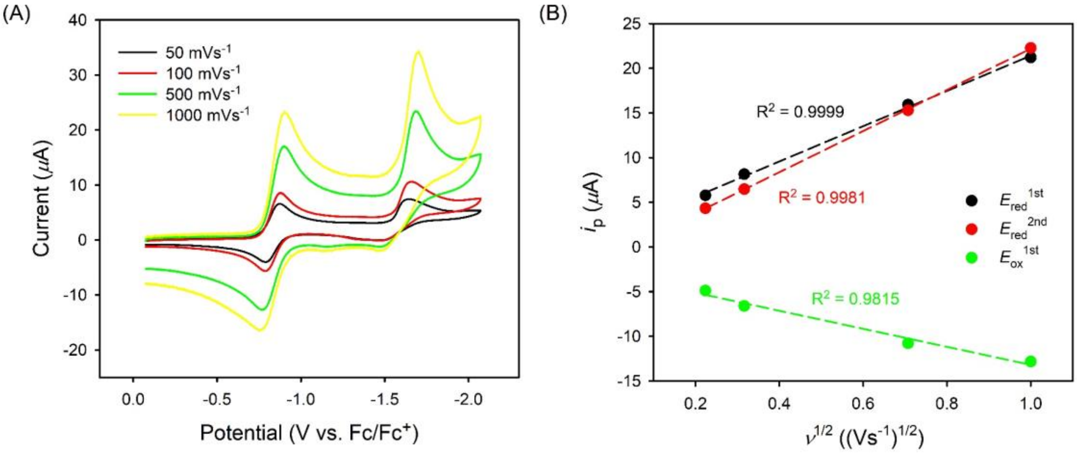

Figure S24. (A) Cyclic voltammograms of 1 mM [**1**][$(ClO_4)_2$] in CH_3CN with various scan rates. Conditions: 0.1 M NBu_4PF_6, Pt, and Ag/$AgNO_3$ as the supporting electrolyte, working electrode, and reference electrode, respectively. (B) Plot of i_p (μA) and (scan rate)$^{1/2}$.

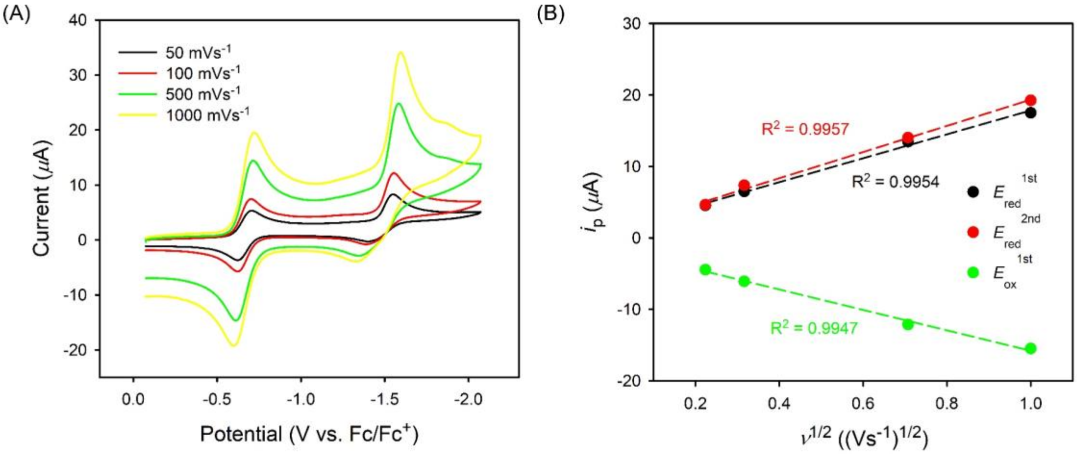

Figure S25. (A) Cyclic voltammograms of 1 mM [**2**][$(ClO_4)_2$] in CH_3CN with various scan rates. Conditions: 0.1 M NBu_4PF_6, Pt, and Ag/$AgNO_3$ as the supporting electrolyte, working electrode, and reference electrode, respectively. (B) Plot of i_p (μA) and (scan rate)$^{1/2}$.

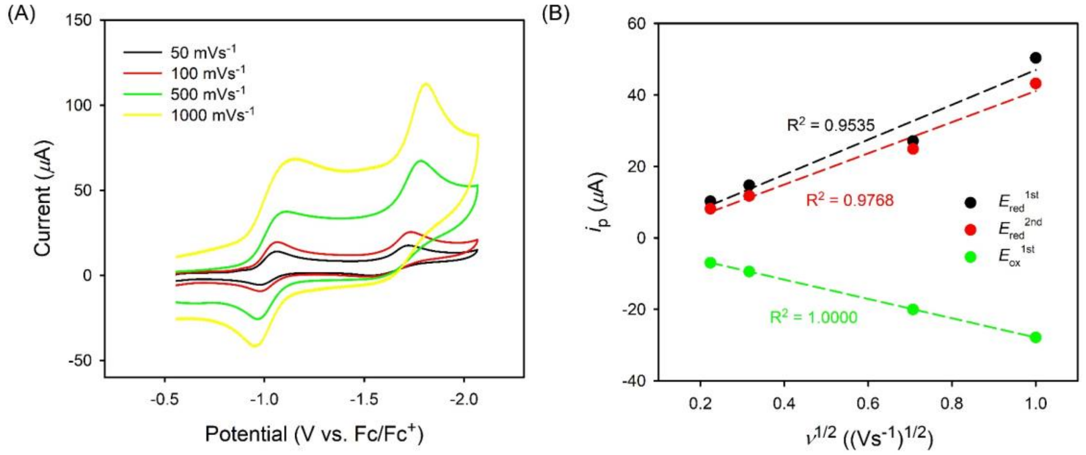

Figure S26. (A) Cyclic voltammograms of 1 mM [**3**][(ClO_4)$_2$] in CH_3CN with various scan rates. Conditions: 0.1 M NBu_4PF_6, Pt, and Ag/$AgNO_3$ as the supporting electrolyte, working electrode, and reference electrode, respectively. (B) Plot of i_p (μA) and (scan rate)$^{1/2}$.

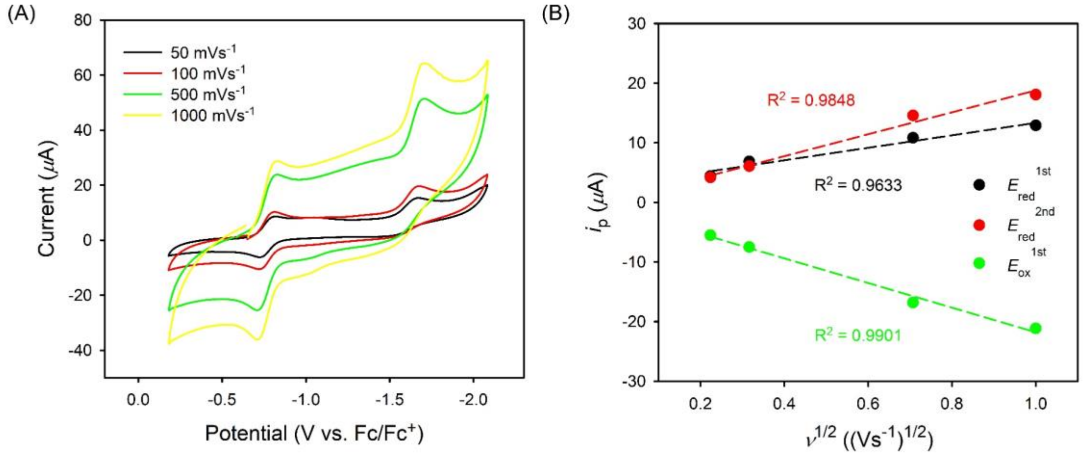

Figure S27. (A) Cyclic voltammograms of 0.5 mM [**4**][(ClO_4)$_2$] in CH_3CN with various scan rates. Conditions: 0.1 M NBu_4PF_6, Pt, and Ag/$AgNO_3$ as the supporting electrolyte, working electrode, and reference electrode, respectively. (B) Plot of i_p (μA) and (scan rate)$^{1/2}$.

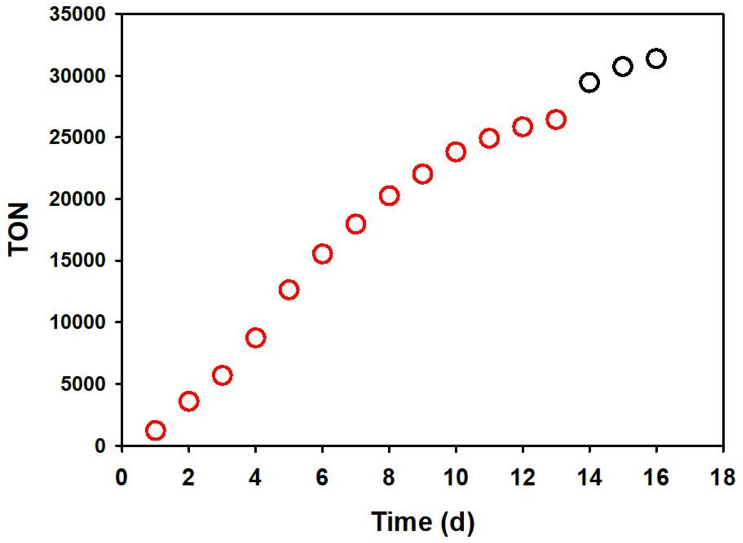

Figure S28. Photocatalytic hydrogen production from a system containing **1** (4.4 μM), Fl (18.6 mM), and TEOA (0.42 M) in MeOH/H_2O (1:1) at pH 10.45 at room temperature upon irradiation (λ = 400 nm, LED). An additional portion of **1** (4.4 μM) was added to the reaction mixture after 14 days.

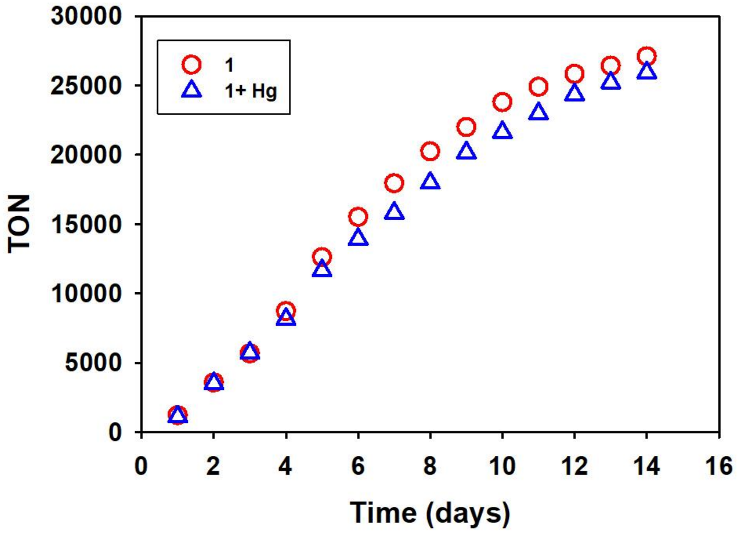

Figure S29. Photocatalytic hydrogen production with and without Hg. Both reactions contain **1** (4.4 μM), Fl (18.6 mM), and TEOA (0.42 M) in MeOH/H_2O (1:1) at pH 10.45 at room temperature upon irradiation (λ = 400 nm, LED).

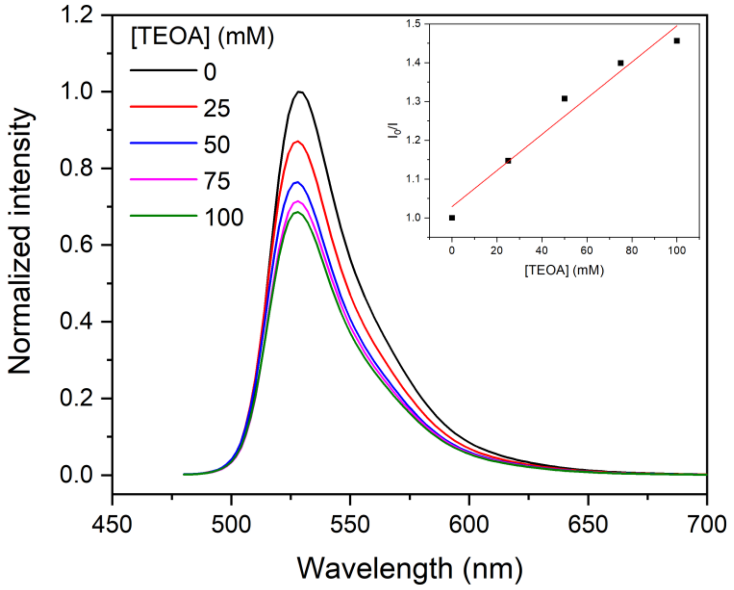

Figure S30. The fluorescence of Fl (0.01 mM in H_2O/CH_3OH) in the presence of TEOA. The excited wavelength: 500 nm. The inset figure is the corresponding Stern-Volmer plot. The Stern-Volmer (SV) constant K_{sv} (i.e. the slope value) is 4.66 M^{-1}. It is known that the excited lifetime of Fl is 4 ns.[1] Therefore, the bimolecular quenching constant, k_q(TEOA) is 1.16×10^9 $M^{-1}s^{-1}$ according to the SV equation.

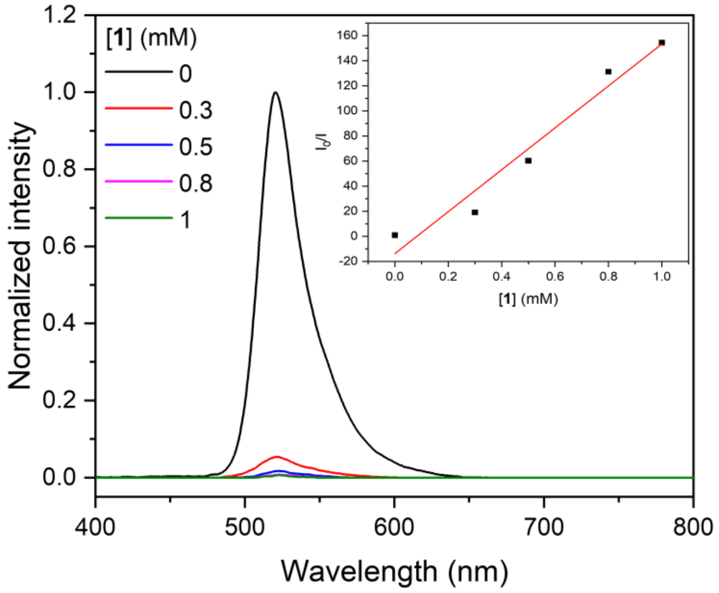

Figure S31. The fluorescence of Fl (0.01 mM in H_2O/CH_3OH) in the presence of **1**. The excited wavelength: 500 nm. The inset figure is the corresponding Stern-Volmer plot. The Stern-Volmer (SV) constant K_{sv} (i.e. the slope value) is 1.67×10^5 M^{-1}. It is known that the excited lifetime of Fl is 4 ns.[1] Therefore, the bimolecular quenching constant, k_q(**1**) is 4.18×10^{13} $M^{-1}s^{-1}$ according to the SV equation. Nevertheless, this apparent value way exceeds the diffusion-control limit, implying interaction between **1** and Fl.

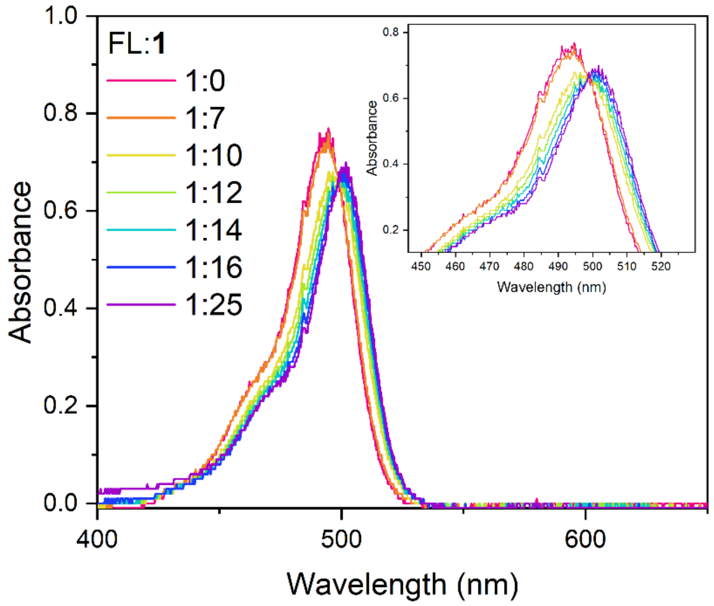

Figure S32. The absorption spectra of Fl (0.01 mM in H_2O/CH_3OH) in the presence of different equivalents of **1**.

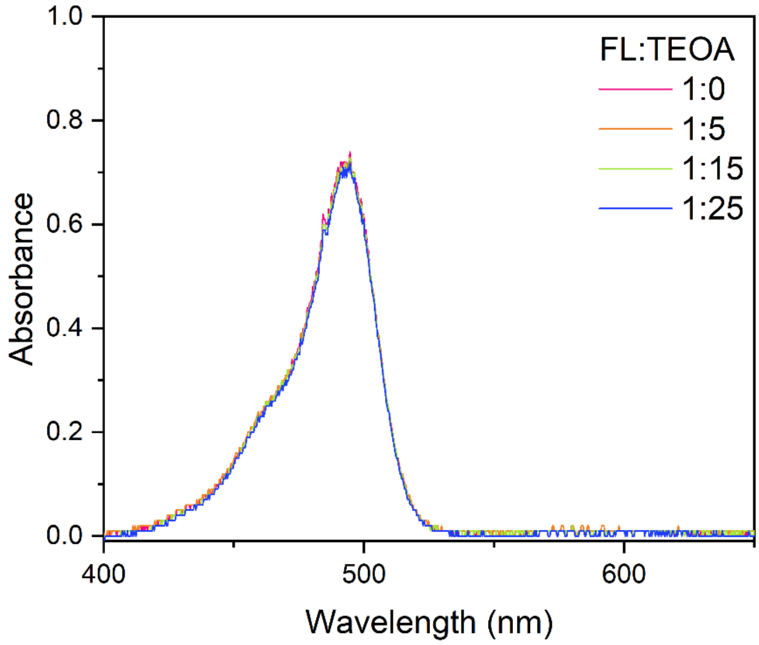

Figure S33. The absorption spectra of Fl (0.01 mM in H_2O/CH_3OH) in the presence of different equivalents of TEOA.

The considerations of the oxidative quenching pathway

The figure below illustrates the energy level diagram for the species involved in the oxidative quenching pathway. Based on the redox potentials, it is expected that the oxidative quenching pathway can occur. However, Stern-Volmer and titration experiments indicate that complex **1** interacts with Fl to form a new complex, quenching fluorescence. Furthermore, in the photocatalytic system, the concentration of complex **1** is significantly lower than that of TEOA and the photosensitizers. Consequently, it is highly unlikely that the oxidative quenching pathway is the dominant mechanism.

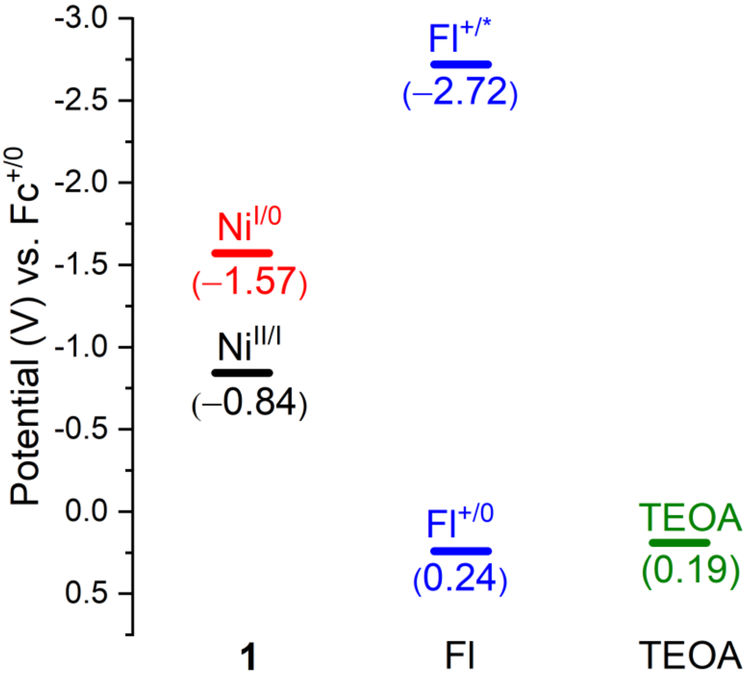

(A)

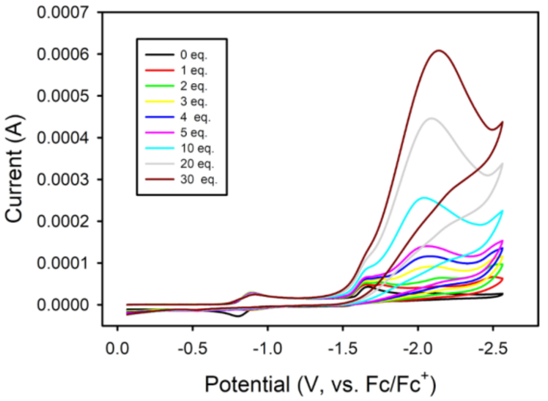

HOAc	TOF (s^{-1})	η (V)
10 μL (1 eq.)	-	-
20 μL (2 eq.)	-	-
30 μL (30 eq.)	-	-
40 μL (4 eq.)	-	-
50 μL (5 eq.)	-	-
100 μL (10 eq.)	9.51	0.36
200 μL (20 eq.)	16.95	0.38
300 μL (30 eq.)	23.52	0.39

(B)

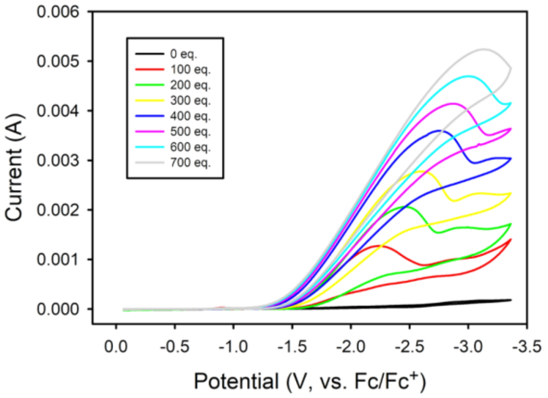

HOAc	TOF (s^{-1})[a]	η (V)[a]
28 μL (100eq.)	429 ± 39	0.38 ± 0.00
56 μL (200eq.)	1,317 ± 40	0.44 ± 0.04
84 μL (300eq.)	2,311 ± 232	0.47 ± 0.03
112 μL (400eq.)	3,551 ± 404	0.52 ± 0.03
140 μL (500eq.)	4,925 ± 500	0.57 ± 0.03
168 μL (600eq.)	6,401 ± 541	0.62 ± 0.03
196 μL (700eq.)	8,114 ± 735	0.67 ± 0.03

Figure S34. CV diagrams of 1mM [**1**][(ClO_4)$_2$] in CH_3CN with (A) 0 – 30eq. and (B) 0 – 700eq. of acetic acid added. Conditions: NBu_4ClO_4, glassy carbon and Ag/$AgNO_3$ as the supporting electrolyte, working electrode, and reference electrode, respectively. Scan rate: 300 mV/s.

Electrocatalytic activity of complex 1

Figure S34 exhibited the electrocatalytic activity of complex **1** in the presence of acetic acid. As the concentration of acid increased, the corresponding electrocatalytic current increased as well. Even though several hundred equivalents of acid were added, the ideal *S*-shape electrocatalytic voltammogram used to determine the TOF still cannot be achieved. Here, Instead, the peak current of electrocatalytic voltammograms was used to estimate the lowest limit of TOF. It is worth emphasizing that the reported TOF here *does not* reflect the TOF of complex **1**. It only provides the information of the lower limit of TOF from **1**.

Note: The calculation of TOF[2] and overpotential:[3]

$$k_{\mathrm{obs}}(\mathrm{TOF}, \mathrm{s}^{-1}) = 1.94 \times \ \nu \ \times \ (\frac{i_{\mathrm{cat}}}{i_{\mathrm{p}}})^2$$

v : scan rate (Vs^{-1})

i_{cat} : the catalytic current in the presence of HOAc (μA)

i_p : the current of reduction peak of Ni complexes without addition of HOAc (μA). (the dilute correct of i_p values were included, where $i_p = i_{p,exp}$ * (initial volume/final volume).

$$\eta = \ E^T_{1/2} - \ E_{cat/2}$$

$E^T_{1/2}$: the theoretical half-wave potential for reducing HOAc to H_2 in CH_3CN without Ni catalysts.

$E_{cat/2}$: the experimental value of half-wave potential for reducing HOAc to H_2 in CH_3CN with Ni catalysts.

$$E^T_{1/2} = \ E^0_{H^+/H_2} - \left(\frac{2.303RT}{F}\right) p\mathrm{K}_a + \varepsilon_D - \frac{RT}{2F} ln \frac{C_0}{C^0_{\mathrm{H}_2}}$$

$E^0_{H^+/H_2}$: -0.07 V, the standard potential for the reduction of protons in CH_3CN.

R: 8.314 $JK^{-1}mol^{-1}$, universal gas constant.

T: 298 K, temperature.

$p\mathrm{K}_a$: 23.5, acid dissociation constant of HOAc in CD_3CN.

ε_D: 0.04 V, measure of how fast is the diffusion of the products with respect to that of the reactant.

F: 96485.3 $C{\cdot}mol^{-1}$, Faraday constant.

C_0: M, the total concentration of addition of HOAc.

$C^0_{\mathrm{H}_2}$: 0.0033 M, the concentration of dissolved hydrogen corresponding to a partial pressure of 105 Pa.

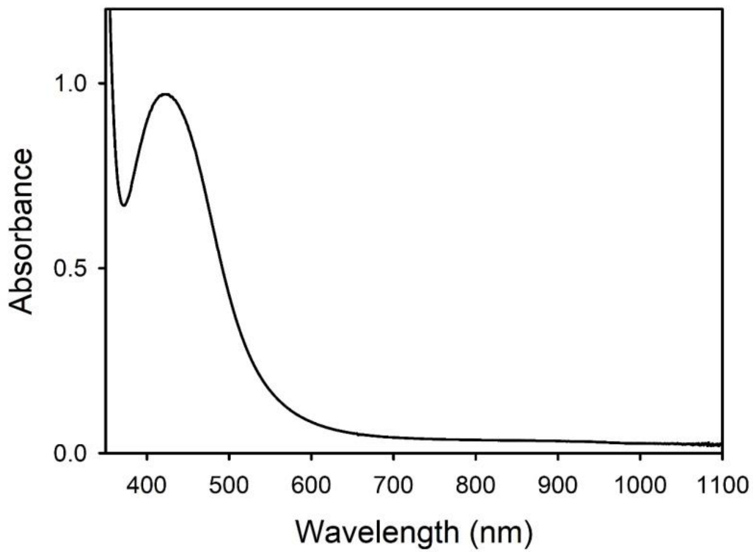

Figure S35. UV-vis-NIR spectrum of [**1**][(ClO_4)$_2$] (2 mM) in H_2O/CH_3OH/CH_3CN (2:2:1).

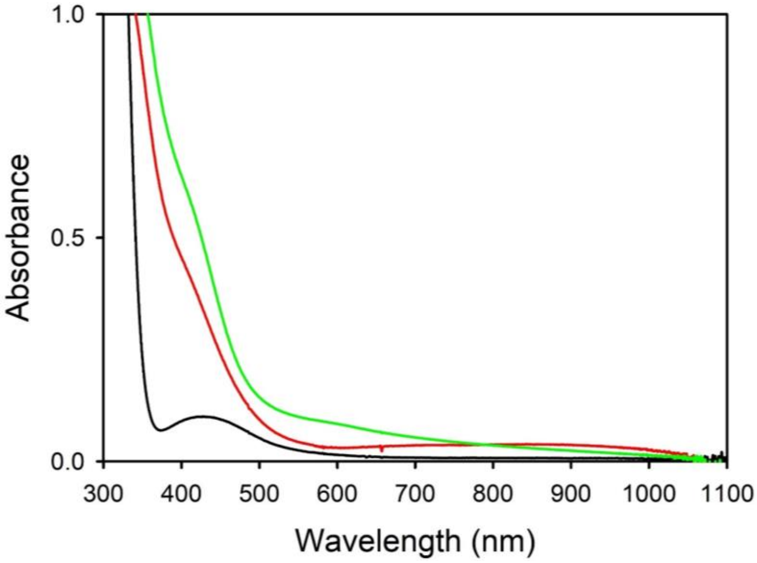

Figure S36. UV-vis-NIR spectra of (black) [**1**][(ClO_4)$_2$] (0.16 mM) treated with (red) 1eq. and (green) 2eq. $CoCp^*_2$ in CH_3CN.

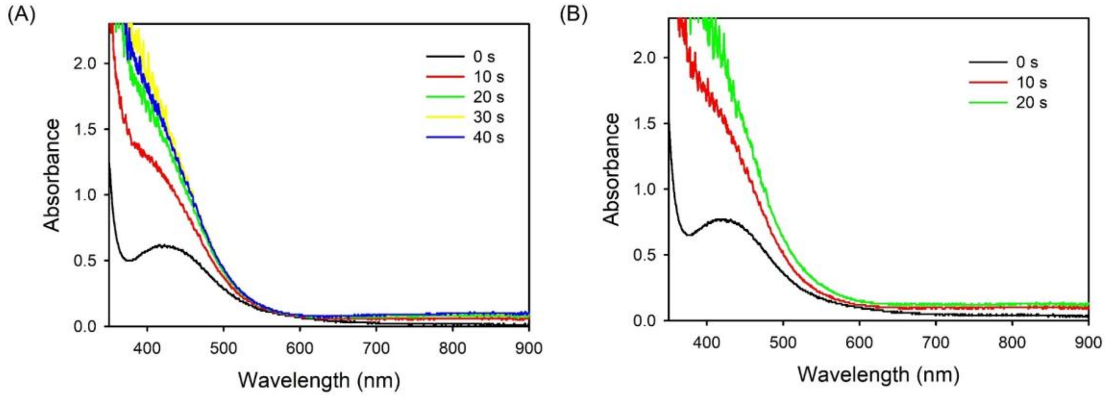

Figure S37. Monitoring UV-vis-NIR spectral changes of 1mM [**1**][$(ClO_4)_2$] in CH_3CN at the applied potentials of (A) -1.05 V and (B) -1.77 V.

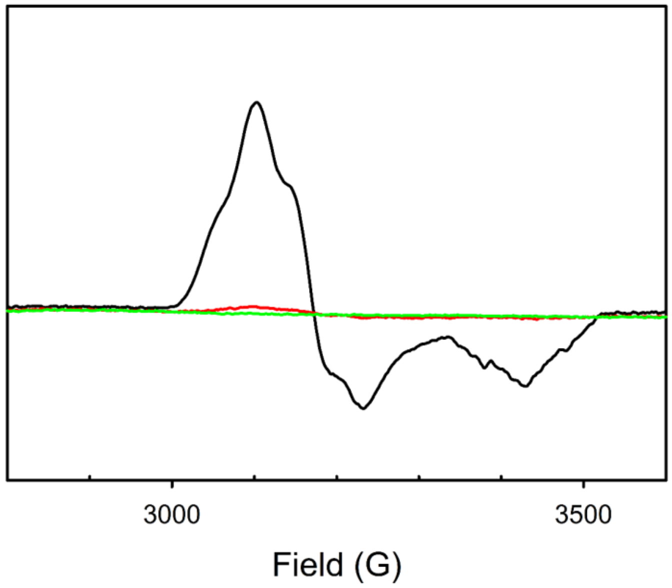

Figure S38. The X-band EPR spectrum of [**1**][$(ClO_4)_2$] (1.5 mM) in CH_3CN after applying a potential of -1.3 V for 30 minutes at 100 K (black); the spectra after applying a potential of -1.9 V for 30 minutes at 100 K (red) and room temperature (green). Sweep range: 2800-3600 G; microwave frequency: 9.62 GHz; modulation amplitude: 0.0004 T; modulation frequency: 100 kHz; power: 1 mW

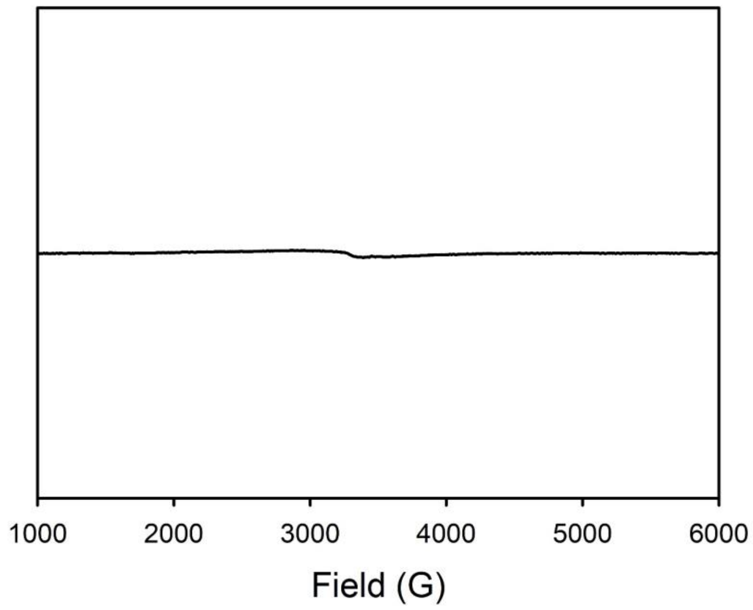

Figure S39. The X-band EPR spectrum of Ni(COD)$_2$ and PN1 in 1: 2 ratio in CH_3CN at 77K. Sweep range: 1000-6000 G; microwave frequency: 9.48 GHz; modulation amplitude: 0.0002 T; modulation frequency: 100 kHz; power: 2 mW

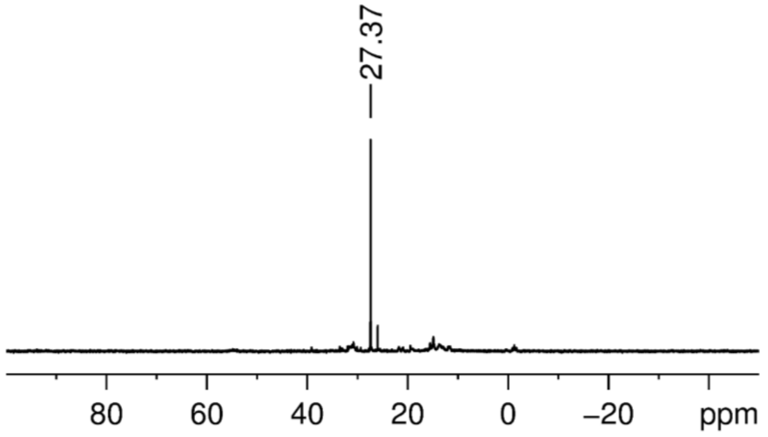

Figure S40. ^{31}P NMR spectrum of Ni(COD)$_2$ and PN1 in 1: 2 ratio in CD_3CN at room temperature.

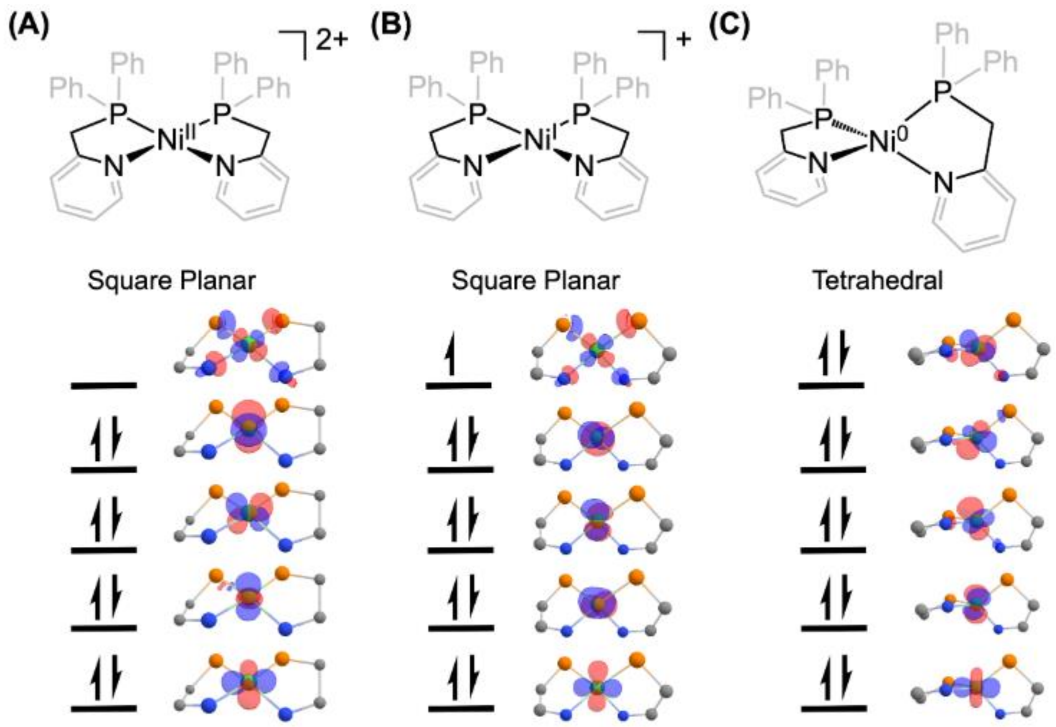

Figure S41. *d*-orbital occupations of Complex **1** for (A) $[Ni(II)]^{2+}$, (B) $[Ni(I)]^{+}$ and (C) $[Ni(0)]^{0}$. Hydrogen atoms and structures marked in grey were omitted for clarity.

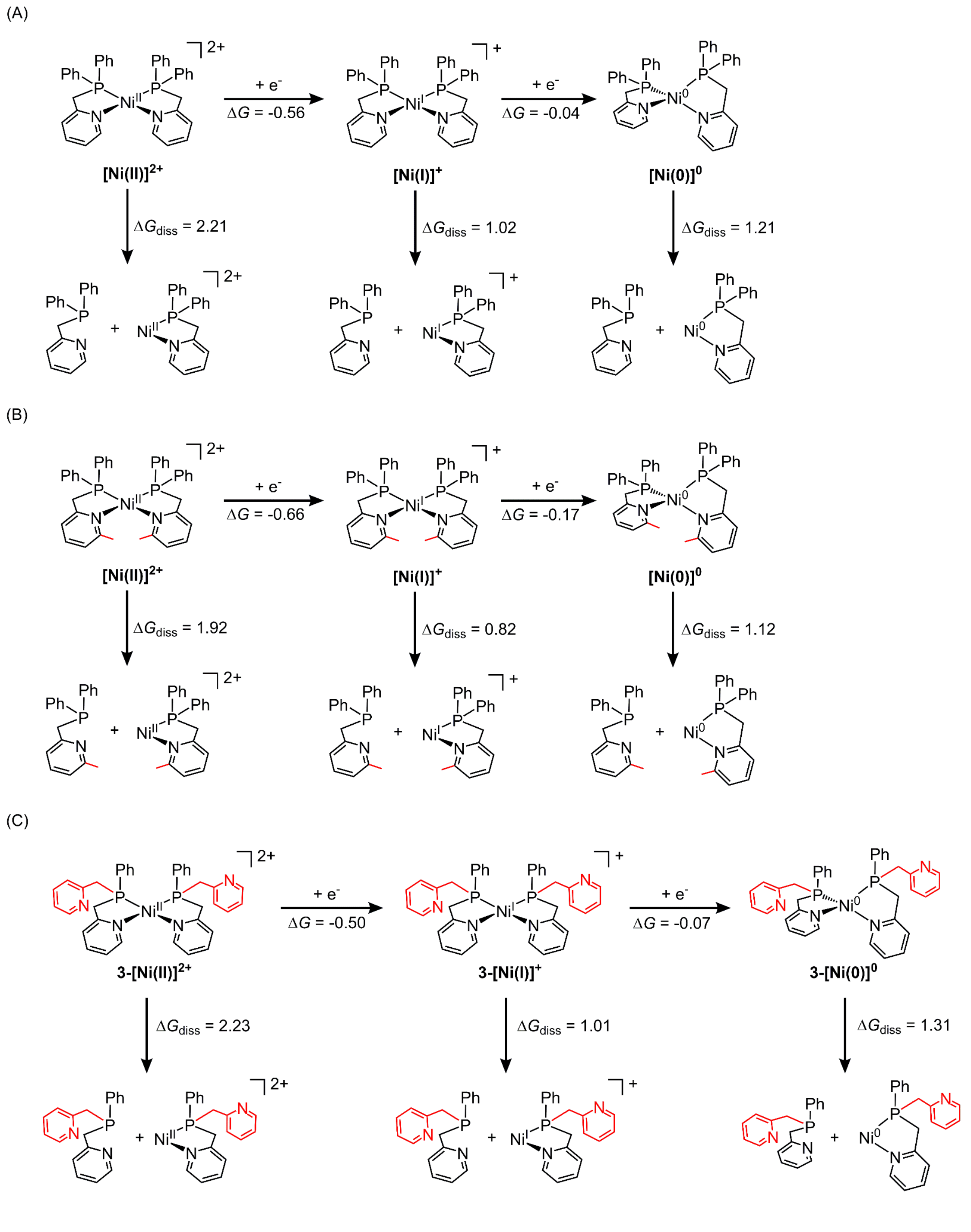

Figure S42. Ligand dissociation energies for (A) **1**, (B) **2**, and (C) **3** in their $[Ni(II)]^{2+}$, $[Ni(I)]^{+}$, and $[Ni(0)]^{0}$ states. Red skeletons mark the difference between catalysts. The unit is eV.

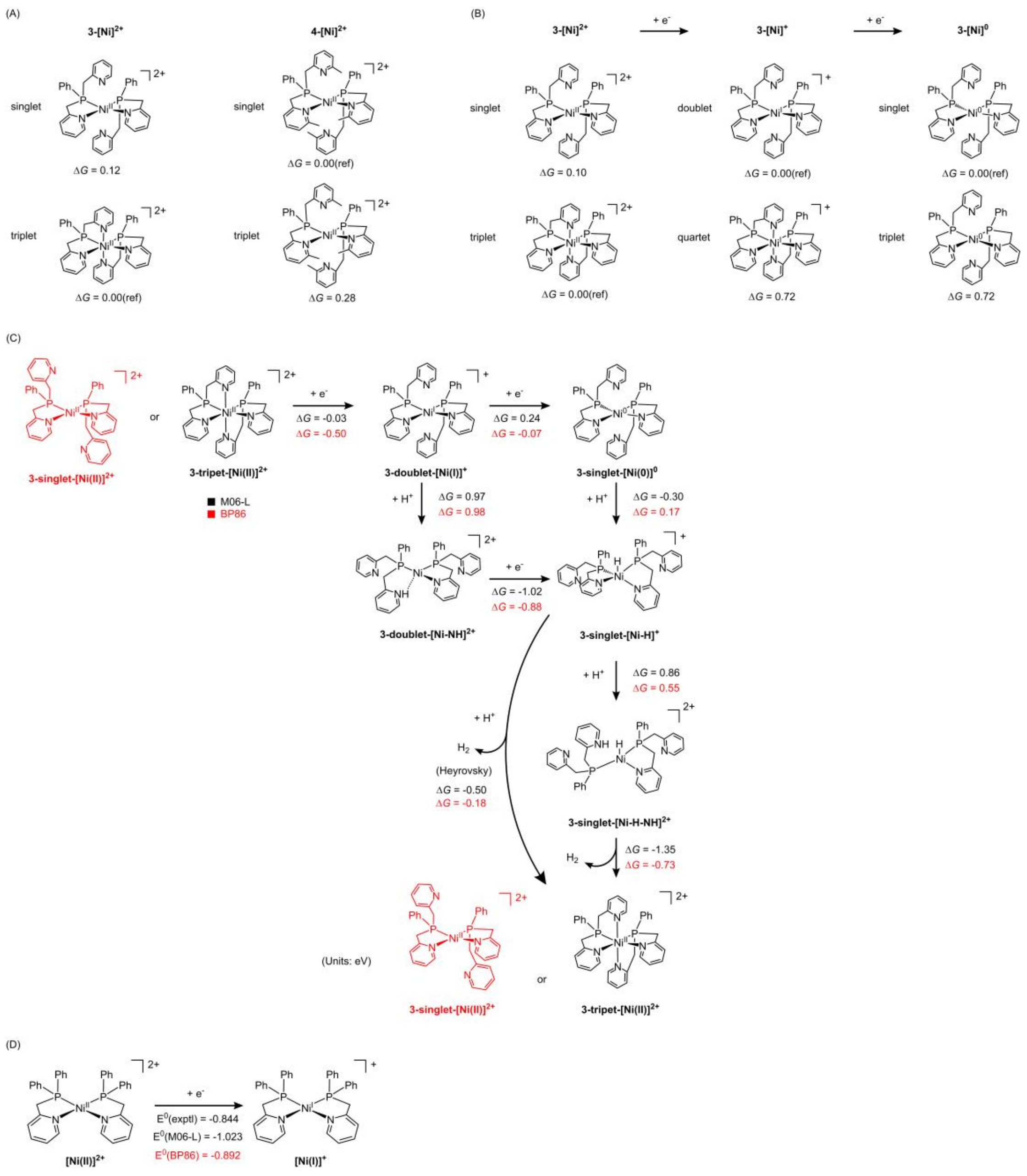

Scheme S1. Relative Gibbs free energies of (A) different spin states for complexes **3** and **4**, (B) spin states as reduction progresses for complex **3**, (C) HER Gibbs free energy profile for complex **3**, and (D) redox potential for complex **1**. To match the experimental environment, the solvent is acetonitrile for (A) and (D), and water for (B) and (C). Unless specified otherwise, the computational approach used in this scheme was M06-L/def2-SVP//M06-L/def2-SVP. The unit is eV.

Discussion Regarding Complex 3 under the M06−L Functional

Using M06−L functional, the triplet state of complex **3** is calculated to be the ground state and is more stable by 0.12 eV in acetonitrile (Scheme S1 (A)), aligning with our experimental observation. This stability is attributed to the bonding of methylpyridine groups to the Ni center, which stabilizes the high−spin Ni center. It is important to note that the original functional, BP86, inherently favors the low−spin state and thus predicted the singlet state as the ground state. Consequently, we opted for M06−L to accurately predict the energy difference between these spin states.[4]

Further, our analysis shows that as reduction progresses, intermediates of complex **3** tend to favor low−spin states (Scheme S1 (B)). We have also observed that after one electron reduction occurs at complex **3**, the reaction prefers the addition of a second electron (ΔG = 0.28) over protonation (ΔG = 1.01), as depicted in Scheme S1 (C). This preference maintains the EECC HER pathways under the M06−L level. It was observed that employing the BP86 functional led to the same reaction mechanism.

Regarding the difference between complexes **3** and **4**, we identified that the presence of methyl groups on pyridine groups in complex **4** introduces steric hindrance in the 6−coordinated geometry. This hinders the formation of a stable 6−coordinated conformation when the spin state is triplet. Post−geometry optimization, we found that only 4−coordinated conformations are feasible in both singlet and triplet states, leading to a singlet ground state for complex **4**.

Lastly, while BP86 is more adept at predicting redox potentials (Scheme S1 (D)), thereby offering more accurate HER energetics for complex **1**, where the ground state is singlet, M06−L is preferable for providing accurate descriptions of spin states. Therefore, M06−L was used solely for calculations pertaining to complex **3**.

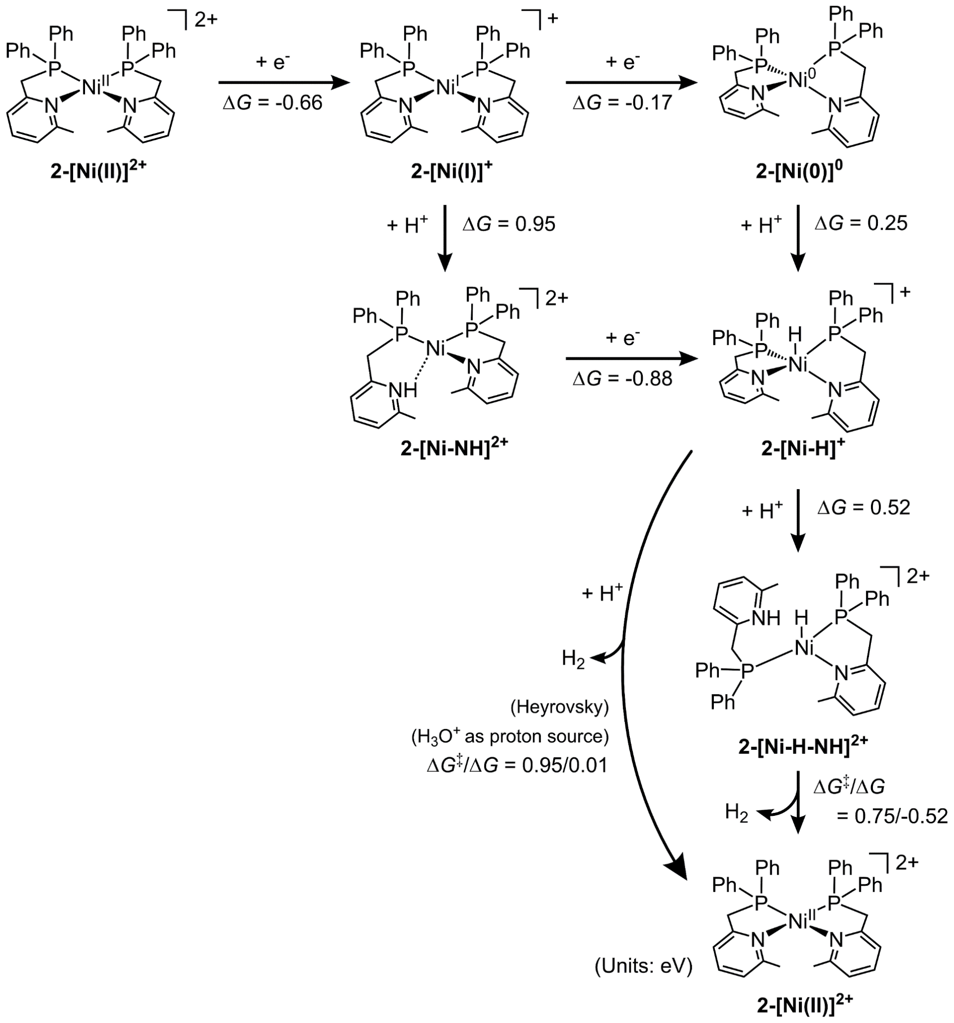

Scheme S2. Free energy profile for the HER of **2** in an aqueous medium, calculated at U = −1.53 V vs. Fc^{+}/Fc and pH 10.45.

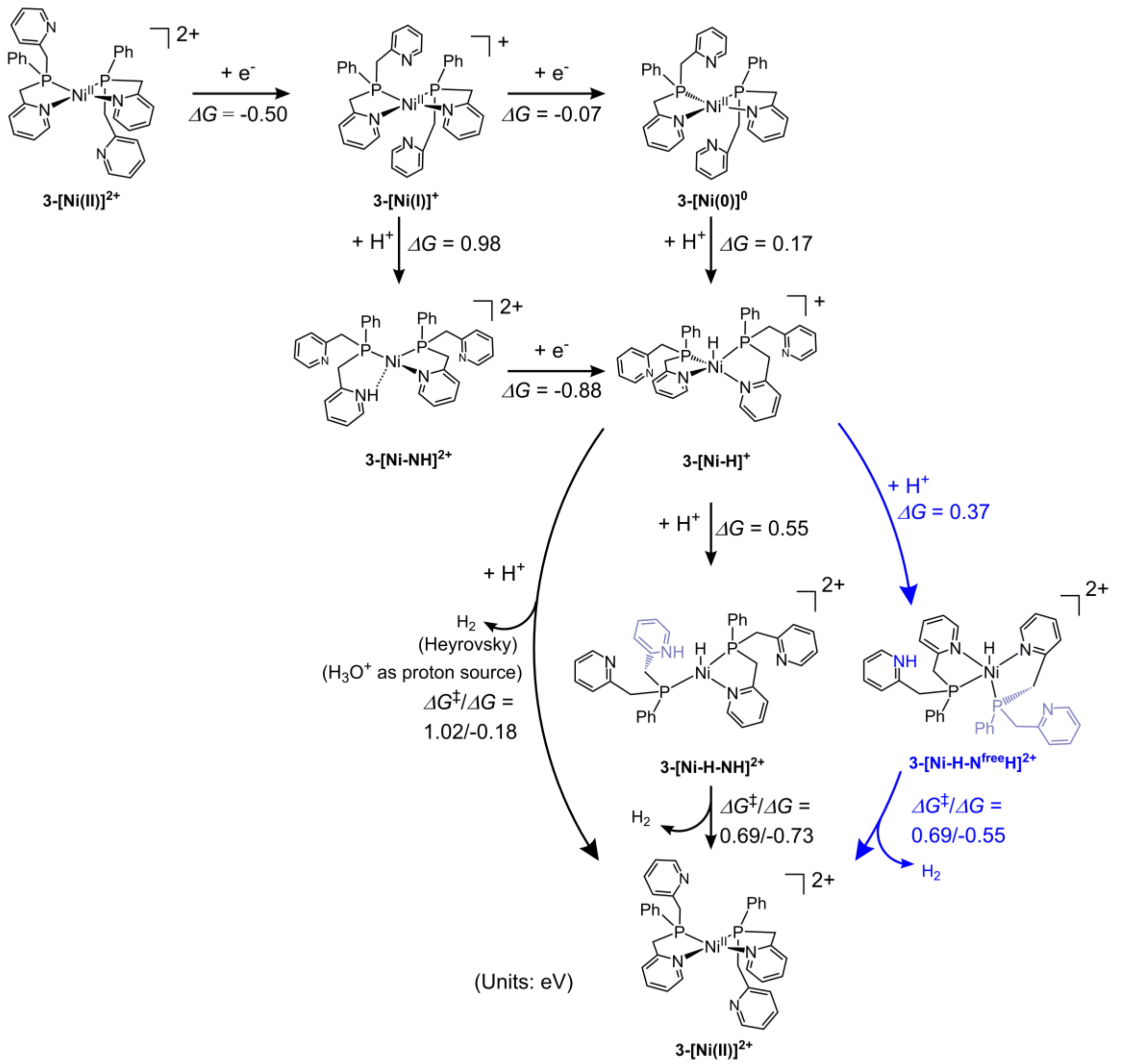

Scheme S3. Free energy profile for the HER of **3** in an aqueous medium, calculated at U = −1.53 V vs. Fc^{+}/Fc and pH 10.45.

Table S1. Crystallographic data of $[Ni(PN1)_2][(ClO_4)_2]$ ([**1**]$[(ClO_4)_2]$)

	$[Ni(PN1)_2][(ClO_4)_2]$
Chemical formula	$C_{36}H_{32}Cl_2N_2NiO_8P_2$
Fw	812.18
T, K	145(2)
Crystal system	Monoclinic
Space group	$P2_1/n$
a, Å	9.2278(6)
b, Å	25.407(2)
c, Å	15.009(1)
α, deg	90
β, deg	95.420(2)
γ, deg	90
V, Å ^{3}	3503.1(4)
Z	4
D_{cala}/g cm^{-3}	1.540
GOF[a] on F^2	1.070
R_1,[b] %	0.0372
wR_2,[c] %	0.0807
CCDC number	2242024

[a]GOF=$\{\Sigma[w(F_o^2–F_c^2)^2]/(M\text{-}N)\}1/2$($M$=number of reflections, N= number of parameters refined)

[b]$R_1 = \Sigma||F_o| – |F_c||/\Sigma||F_o|$.

[c]$wR_2 = \{\Sigma[w(F_o^2 – F_c^2)^2]/\Sigma[w(F_o^2)^2]\}^{1/2}$.

Table S2. The selected bond length and angles of $[Ni(PN1)_2][(ClO_4)_2]$ ([**1**]$[(ClO_4)_2]$)

Bond length (Å)		Bond angle (degree)	
Ni1-N1	1.928(2)	N1-Ni1-N2	93.62(7)
Ni1-N2	1.955(2)	N1-Ni1-P1	82.64(5)
Ni1-P1	2.1592(5)	N2-Ni1-P1	163.78(5)
Ni1-P2	2.1697(5)	N1-Ni1-P2	174.72(5)
		N2-Ni1-P2	82.59(5)
		P1-Ni1-P2	102.01(2)

Table S3. Crystallographic data of [Ni(PN1^{Me})$_2$][(ClO$_4$)$_2$] ([**2**][(ClO$_4$)$_2$])

	[Ni(PN1^{Me})$_2$][(ClO$_4$)$_2$]
Chemical formula	$C_{38}H_{36}Cl_2N_2NiO_8P_2$
Fw	840.24
T, K	100(2)
Crystal system	Monoclinic
Space group	$P2_1/n$
a, Å	9.902 (1)
b, Å	24.250(3)
c, Å	15.324(2)
α, deg	90
β, deg	95.395(3)
γ, deg	90
V, Å ^{3}	3663.3(7)
Z	4
D_{cala}/g cm^{-3}	1.523
GOF[a] on F^2	1.232
R_1,[b] %	0.0736
wR_2,[c] %	0.1230
CCDC number	2242025

[a]GOF=$\{\Sigma[w(F_o^2–F_c^2)^2]/(M-N)\}1/2$($M$=number of reflections, N= number of parameters refined)

[b]$R_1 = \Sigma||F_o| – |F_c||/\Sigma||F_o|$.

[c]$wR_2 = \{\Sigma[w(F_o^2 – F_c^2)^2]/\Sigma[w(F_o^2)^2]\}^{1/2}$.

Table S4. The selected bond length and angles of $[Ni(PN1^{Me})_2][(ClO_4)_2]$ ([**2**][$(ClO_4)_2$])

Bond length (Å)		Bond angle (degree)	
Ni1-N1	1.959(3)	N1-Ni1-N2	98.2(1)
Ni1-N2	2.011(3)	N1-Ni1-P1	81.6(1)
Ni1-P1	2.161(1)	N2-Ni1-P1	163.0(1)
Ni1-P2	2.165(1)	N1-Ni1-P2	172.3(1)
		N2-Ni1-P2	82.45(9)
		P1-Ni1-P2	100.03(4)

Table S5. Crystallographic data of $[Ni(PN2)_2][(ClO_4)_2]$ ([**3**]$[(ClO_4)_2]$)

	$[Ni(PN2)_2][(ClO_4)_2]$
Chemical formula	$C_{36}H_{34}Cl_2N_4NiO_8P_2$
Fw	842.22
T, K	150(2)
Crystal system	Orthorhombic
Space group	Aba2
a, Å	20.0118(6)
b, Å	18.0013(5)
c, Å	10.2777(3)
α, deg	90
β, deg	90
γ, deg	90
V, Å ^{3}	3702.4(2)
Z	4
D_{cala}/g cm^{-3}	1.511
GOF[a] on F^2	1.036
R_1,[b] %	0.0338
wR_2,[c] %	0.0829
CCDC number	2007060

[a]GOF=$\{\Sigma[w(F_o^2–F_c^2)^2]/(M-N)\}1/2$($M$=number of reflections, N= number of parameters refined)

[b]$R_1 = \Sigma||F_o| – |F_c||/\Sigma||F_o|$.

[c]$wR_2 = \{\Sigma[w(F_o^2 – F_c^2)^2]/\Sigma[w(F_o^2)^2]\}^{1/2}$.

Table S6. The selected bond length and angles of $[Ni(PN2)_2][(ClO_4)_2]$ ([**3**]$[(ClO_4)_2]$)

Bond length (Å)		Bond angle (degree)	
Ni1-N1	1.951(2)	N1-Ni1-N1’	97.4(2)
Ni1-P1	2.1476(8)	N1-Ni1-P1	84.90(7)
		N1’-Ni1-P1	167.25(8)
		N1-Ni1-P1’	167.25(8)
		N1’-Ni1-P1’	84.90(7)
		P1-Ni1-P1’	95.69(4)

Table S7. Crystallographic data of $[Ni(PN2^{Me})_2][(ClO_4)_2]\cdot 2.5CH_3CN$ ([**4**]$[(ClO_4)_2]\cdot 2.5CH_3CN$)

	$[Ni(PN2^{Me})_2][(ClO_4)_2]\cdot 2.5CH_3CN$
Chemical formula	$C_{90}H_{99}Cl_4N_{13}Ni_2O_{16}P_4$
Fw	2001.92
T, K	150(2)
Crystal system	Orthorhombic
Space group	$P2_12_12_1$
a, Å	16.043(1)
b, Å	18.785(2)
c, Å	31.099(3)
α, deg	90
β, deg	90
γ, deg	90
V, Å ^{3}	9372.3(14)
Z	4
D_{cala}/g cm^{-3}	1.419
GOF^a on F^2	1.079
R_1,^{b} %	0.0629
wR_2,^{c} %	0.1476
CCDC number	2007061

^{a}GOF=$\{\Sigma[w(F_o^2 - F_c^2)^2]/(M-N)\}1/2$($M$=number of reflections, N= number of parameters refined)

$^b R_1 = \Sigma||F_o| - |F_c||/\Sigma|F_o|$.

$^c wR_2 = \{\Sigma[w(F_o^2 - F_c^2)^2]/\Sigma[w(F_o^2)^2]\}^{1/2}$.

Note: The Flack parameter is 0.577, which means a racemic twin crystal.

Table S8. The selected bond length and angles of $[Ni(PN2^{Me})_2][(ClO_4)_2]\cdot 2.5CH_3CN$ ([**4**]$[(ClO_4)_2]\cdot 2.5CH_3CN$)

Bond length (Å)		Bond angle (degree)	
Ni1-N1	1.965(5)	N1-Ni1-N3	98.5(2)
Ni1-N3	1.974(5)	N1-Ni1-P1	83.5(2)
Ni1-P1	2.133(2)	N3-Ni1-P1	169.2(2)
Ni1-P2	2.139(2)	N1-Ni1-P2	168.9(2)
Ni2-N7	1.963(5)	N3-Ni1-P2	83.7(2)
Ni2-N5	1.963(5)	P1-Ni1-P2	96.38(7)
Ni2-P3	2.126(2)	N7-Ni2-N5	97.9(2)
Ni2-P4	2.143(2)	N7-Ni2-P3	167.2(2)
		N5-Ni2-P3	83.9(2)
		N7-Ni2-P4	83.5(2)
		N5-Ni2-P4	167.9(2)
		P3-Ni2-P4	97.46(7)

Table S9. Photocatalytic HER using photosensitizers of fluorescein (Fl) and eosin yellow (EY) at different concentrations.[a]

Entry	Catalyst	C_{PS} (mM)	TON[b]
1	**1**	EY (18.6)	610 ± 70
2	**1**	Fl (18.6)	1190 ± 90
3	**1**	Fl (1.9)	190 ± 30
4	**1**	Fl (186)	0
5	**2**	EY (18.6)	570 ± 70
6	**2**	Fl (18.6)	1920
7	**2**	Fl (1.9)	110 ± 40
8	**2**	Fl (186)	0
9	**3**	EY (18.6)	58 ± 8
10	**3**	Fl (18.6)	200 ± 100
11	**4**	EY (18.6)	130
12	**4**	Fl (18.6)	170
13	**4**	Fl (9.3)	480
14	**4**	Fl (37.2)	120

[a] [Catalyst]= 44 μM; [TEOA] = 0.42 M; Solvent: MeOH/ H_2O (v: v= 1: 1); Irradiation: 400 nm; Reaction time: 2 days. The reaction is equipped with a round-bottom glass flask. [b] Each reaction is repeated three times for entries 1-10.

Table S10. Photocatalytic hydrogen evolution reaction with various sacrificial electron donors including ascorbic acid (AA), triethylamine (TEA), triethanolamine (TEOA)[a]

Entry	Catalyst	C_{SED} (M)	TON [b]
1	**1**	AA (0.42)	0
2	**1**	TEA (0.42)	50 ± 2
3	**1**	TEOA (0.42)	1190 ± 90
4	**1**	TEOA (0.04)	350 ± 30
5	**1**	TEOA (4.2)	160 ± 30
6	**2**	AA (0.42)	0
7	**2**	TEA (0.42)	1140 ± 70
8	**2**	TEOA (0.42)	1920
9	**2**	TEOA (0.04)	230 ± 80
10	**2**	TEOA (4.2)	230 ± 50
11	**4**	AA (0.42)	0
12	**4**	TEA (0.42)	100
13	**4**	TEOA (0.42)	1840
14	**4**	TEOA (0.84)	390

[a] [Catalyst]= 44 μM; [Fl] = 18.6 mM; Solvent: MeOH/ H_2O (v: v= 1: 1); Irradiation: 400 nm; Reaction time: 2 days for entries 1-12 and 4 days for entries 13-14. The reaction is equipped with a round-bottom glass flask. [b] Each reaction is repeated three times for entries 2-5, 7, 9, 10.

Table S11. Photocatalytic hydrogen evolution reaction with various solvent systems.[a]

Entry	Catalyst	Solvent	TON [b]
1	**1**	CH_3CN/ H_2O (1:1)	90 ± 20
2	**1**	EtOH/ H_2O (1:1)	500 ± 200
3	**1**	MeOH/ H_2O (1:1)	1190 ± 80
4	**1**	MeOH/ H_2O (4:1)	620 ± 20
5	**1**	MeOH/ H_2O (1:4)	120 ± 10
6	**1**	MeOH/ H_2O (5:0)	80 ± 10
7	**1**	MeOH/ H_2O (0:5)	55 ± 3
8	**2**	CH_3CN/ H_2O (1:1)	180 ± 70
9	**2**	EtOH/ H_2O (1:1)	220 ± 10
10	**2**	MeOH/ H_2O (1:1)	1920
11	**2**	MeOH/ H_2O (4:1)	1500 ± 150
12	**2**	MeOH/ H_2O (1:4)	600 ± 120
13	**2**	MeOH/ H_2O (5:0)	720 ± 50
14	**2**	MeOH/ H_2O (0:5)	81 ± 50
15	**3**	CH_3CN/ H_2O (1:1)	685 ± 20
16	**3**	MeOH/ H_2O (1:1)	707 ± 20
17	**4**	MeOH/ H_2O (1:1)	110 ± 5
18	**4**	EtOH/ H_2O (1:1)	690
19	**4**	EtOH/ H_2O (4:1)	390
20	**4**	EtOH/ H_2O (1:4)	250
21	**4**	EtOH/ H_2O (5:0)	480
22	**4**	EtOH/ H_2O (0:5)	170

[a] [Catalyst]= 44 μM; [Fl] = 18.6 mM; [TEOA] = 0.42 M; Irradiation: 400 nm; Reaction time: 2 days. [b] Each reaction is repeated three times for entries 1-9, 11-17. The reaction is equipped with a round-bottom glass flask.

Table S12. Photocatalytic hydrogen evolution reaction of complex **1** at different pH values.[a]

Entry	pH [b]	TON[g]
1[b]	9.45	320 ± 40
2[c]	9.62	350 ± 30
3[d]	10.45	1190 ± 80
4[e]	10.77	160 ± 30
5[f]	11.85	150 ± 20

[a] [Catalyst]= 44 μM; [Fl] = 18.6 mM; Irradiation: 400 nm; Reaction time: 2 days. The reaction is equipped with a round-bottom glass flask. [b][TEOA] = 0.42 M; Adjusting the pH value to 9.45 by incorporating acetic acid. [c][TEOA] = 0.04 M [d][TEOA] = 0.42 M. [e][TEOA] = 4.2 M. [f][TEOA] = 0.42 M; Adjusting the pH value to 11.85 by incorporating NaOH. [g]Each reaction is repeated two times.

Table S13. Photocatalytic hydrogen evolution reaction of complexes **1** and **2** at various concentrations.[a]

Entry	Catalyst	[Catalyst] (μM)	TON
1	**1**	2.2	1990
2	**1**	4.4	2320
3	**1**	44	1280
4	**1**	440	70
5	**2**	4.4	2080
6	**2**	44	1920
7	**2**	440	12

[a] [Fl] = 18.6 mM; [TEOA] = 0.42 M; Solvent: MeOH/ H_2O (v: v= 1: 1); Irradiation: 400 nm; Reaction time: 2 days. The reaction is equipped with a round-bottom glass flask.

Table S14. Photocatalytic hydrogen evolution reaction using complexes **1-4** as catalysts.[a]

Day	Catalyst **1** (TON)	Catalyst **2** (TON)	Catalyst **3** (TON)[b]	Catalyst **4** (TON)
1	1200	900	1000 ± 200	900
2	3600	3100	2600 ± 600	3200
3	5700	5900	6000 ± 200	6800
4	8800	7700	9300 ± 100	8100
5	12600	8900	12100 ± 500	9300
6	15500	9800	13900 ± 600	10200
7	17900	11000	16100 ± 800	11400
8	20200	11500	17600 ± 200	12500
9	22000	12200	19200 ± 400	13400
10	23800	12700	20800 ± 100	14500
11	24900	13400	22000 ± 500	15100
12	25800	13600	22800 ± 200	16000
13	26400	14000	23700 ± 200	16600
14	27100	14300	24800 ± 500	17300

[a] [Catalyst]= 4.4 μM; [Fl] = 18.6 mM; [TEOA] = 0.42 M; Solvent: MeOH/ H_2O (v: v= 1: 1) for **1** and **2** and EtOH/ H_2O (v: v= 1: 1) for **3** and **4**; Irradiation: 400 nm; Reaction time:14 days. The reaction is conducted in a quartz cell. [b] Each reaction is repeated three times.

Table S15. Energy components of Gibbs free energies. The species calculated using water as solvent are not specifically labeled as such. All values are in Hartree units.

**Species**	E_{elec}	$ZPE+4RT+H_{vib}-TS_s$	***G***
$H_{2(g)}$	-1.1774	-0.0018	-1.1792
$H_3O^+_{(aq)}$	-76.8620	0.0221	-76.8620
$[Ni(II)]^{2+}$@CH_3CN	-3692.3322	0.5285	-3691.8036
$[Ni(I)]^{+}$@CH_3CN	-3692.4754	0.5227	-3691.9527
$[Ni(0)]^{0}$@CH_3CN	-3692.6006	0.5207	-3692.0800
$[Ni(II)]^{2+}$@CH_3CN(M06-L)	-3691.7836	0.5460	-3691.2376
$[Ni(I)]^{+}$@CH_3CN(M06-L)	-3691.9180	0.5391	-3691.3789
$[Ni(0)]^{0}$@CH_3CN(M06-L)	-3692.0220	0.5382	-3691.4838
$[Ni(II)]^{2+}$	-3692.3352	0.5483	-3691.7869
$[Ni(I)]^{+}$	-3692.4763	0.5434	-3691.9329
$[Ni(0)]^{0}$	-3692.6009	0.5412	-3692.0598
$[Ni\text{-}NH]^{2+}$	-3692.9019	0.5560	-3692.3459
$[Ni\text{-}H]^{+}$	-3693.0608	0.5521	-3692.5088
$[Ni\text{-}H\text{-}NH]^{2+}$	-3693.5053	0.5656	-3692.9398
TS-$[Ni\text{-}H\text{-}NH]^{2+}\rightarrow[Ni(II)]^{2+}$	-3693.4745	0.5629	-3692.9116
TS-$[Ni\text{-}H]^{+}+H_3O^{+}\rightarrow[Ni(II)]^{2+}$	-3769.9227	0.5866	-3769.9227
**2-**$[Ni(II)]^{2+}$	-3770.9879	0.6010	-3770.3869
**2-**$[Ni(I)]^{+}$	-3771.1330	0.5963	-3770.5367
**2-**$[Ni(0)]^{0}$	-3771.2622	0.5937	-3770.6685
**2-**$[Ni\text{-}NH]^{2+}$	-3771.5642	0.6090	-3770.9552
**2-**$[Ni\text{-}H]^{+}$	-3771.7174	0.6046	-3771.1129
**2-**$[Ni\text{-}H\text{-}NH]^{2+}$	-3772.1650	0.6178	-3771.5472
**2-**TS-$[Ni\text{-}H\text{-}NH]^{2+}\rightarrow[Ni(II)]^{2+}$	-3772.1343	0.6146	-3771.5197
**2-**TS-$[Ni\text{-}H]^{+}+H_3O^{+}\rightarrow[Ni(II)]^{2+}$	-3848.5812	0.6406	-3848.5812
**3-**$[Ni(II)]^{2+}$	-3803.0982	0.5784	-3802.5198
**3-**$[Ni(I)]^{+}$	-3803.2380	0.5743	-3802.6637
**3-**$[Ni(0)]^{0}$	-3803.3638	0.5720	-3802.7919
**1**-ligand	-1091.9601	0.2611	-1091.6990

diss-$[Ni(II)]^{2+}$	-2600.2721	0.2655	-2600.0067
diss-$[Ni(I)]^{+}$	-2600.4592	0.2629	-2600.1963
diss-$[Ni(0)]^{0}$	-2600.5772	0.2608	-2600.3164
**2**-ligand	-1131.2919	0.2868	-1131.0051
**2**-*diss*-$[Ni(II)]^{2+}$	-2639.6036	0.2925	-2639.3112
**2**-*diss*-$[Ni(I)]^{+}$	-2639.7914	0.2900	-2639.5014
**2**-*diss*-$[Ni(0)]^{0}$	-2639.9095	0.2872	-2639.6222
**3**-ligand	-1147.3410	0.2768	-1147.0642
**3**-*diss*-$[Ni(II)]^{2+}$	-2655.6547	0.2809	-2655.3738
**3**-*diss*-$[Ni(I)]^{+}$	-2655.8405	0.2782	-2655.5623
**3**-*diss*-$[Ni(0)]^{0}$	-2655.9561	0.2765	-2655.6797
**3**-singlet-$[Ni]^{2+}$@MeCN(M06-L)	-3802.5402	0.5773	-3801.9629
**3**-triplet-$[Ni]^{2+}$@MeCN(M06-L)	-3802.5420	0.5747	-3801.9672
**4**-singlet-$[Ni]^{2+}$@MeCN(M06-L)	-3959.8562	0.6852	-3959.1710
**4**-triplet-$[Ni]^{2+}$@MeCN(M06-L)	-3959.8431	0.6823	-3959.1608
$H_{2(g)}$(M06-L)	-1.1718	-0.0015	-1.1733
**3**-singlet-$[Ni]^{2+}$(M06-L)	-3802.5432	0.5975	-3801.9456
**3**-triplet-$[Ni]^{2+}$(M06-L)	-3802.5449	0.5956	-3801.9493
**3**-doublet-$[Ni(I)]^{+}$(M06-L)	-3802.6664	0.5921	-3802.0743
**3**-quartet-$[Ni(I)]^{+}$(M06-L)	-3802.6381	0.5901	-3802.0480
**3**-singlet-$[Ni(0)]^{0}$(M06-L)	-3802.7773	0.5909	-3802.1863
**3**-triplet-$[Ni(0)]^{0}$(M06-L)	-3802.7468	0.5871	-3802.1597
**3-**$[Ni\text{-}NH]^{2+}$(M06-L)	-3803.0972	0.6065	-3802.4907
**3-**$[Ni\text{-}H]^{+}$(M06-L)	-3803.2518	0.6010	-3802.6507
**3-**$[Ni\text{-}H\text{-}NH]^{2+}$(M06-L)	-3803.6860	0.6151	-3803.0708

Reference

1. Sjöback, R.; Nygren, J.; Kubista, M., Absorption and fluorescence properties of fluorescein. *Spectrochim. Acta A* **1995**, *51* (6), L7-L21.
2. Stewart, M. P.; Ho, M.-H.; Wiese, S.; Lindstrom, M. L.; Thogerson, C. E.; Raugei, S.; Bullock, R. M.; Helm, M. L., High Catalytic Rates for Hydrogen Production Using Nickel Electrocatalysts with Seven-Membered Cyclic Diphosphine Ligands Containing One Pendant Amine. *J. Am. Chem. Soc.* **2013**, *135* (16), 6033-6046.
3. Fourmond, V.; Jacques, P.-A.; Fontecave, M.; Artero, V., H2 Evolution and Molecular Electrocatalysts: Determination of Overpotentials and Effect of Homoconjugation. *Inorg. Chem.* **2010**, *49* (22), 10338-10347.
4. Römer, A.; Hasecke, L.; Blöchl, P.; Mata, R. A. A Review of Density Functional Models for the Description of Fe(II) Spin-Crossover Complexes. *Molecules* **2020**, *25*, 5176.

Appendix:

Coordinates of Optimized Structures

2

$H_{2(g)}$

H 0.000000 0.000000 -0.033722

H 0.000000 0.000000 0.733722

4

$H_3O^+_{(aq)}$

O 1.232144 -1.269092 2.029378

H 2.222728 -1.281084 2.037221

H 0.922543 -0.328696 2.065107

H 0.913359 -1.678446 1.184976

73

$[Ni(II)]^{2+}$@CH_3CN

Ni 22.112289 16.703126 10.834687

P 22.556735 18.324910 12.233106

P 21.574513 15.135810 12.265332

N 22.197647 18.124074 9.484534

N 22.140270 15.222665 9.548235

C 21.451583 18.058817 8.351917

C 21.438013 19.093502 7.411154

C 22.223002 20.233623 7.646454

C 22.981054 20.309012 8.825034

C 22.949368 19.240042 9.734374

C 23.680384 19.238106 11.047612

C 21.201450 19.497528 12.613944

C 19.903481 19.257486 12.111557

C 18.877735 20.190450 12.344144

C 19.142139 21.362209 13.073728

C 20.436000 21.606389 13.572070

C 21.466623 20.680543 13.345279

C 23.514791 18.058872 13.767139

C 22.898769 18.192030 15.033801

C 23.643115 17.966606 16.204194

C 24.997409 17.598843 16.122852
C 25.610905 17.452087 14.864907
C 24.875920 17.675581 13.690079
C 22.972260 15.243316 8.475594
C 23.056238 14.171697 7.580898
C 22.251775 13.041794 7.799417
C 21.404890 13.012803 8.918062
C 21.369522 14.116893 9.784287
C 20.542262 14.169722 11.038108
C 22.896816 13.979215 12.788691
C 24.228727 14.192142 12.370003
C 25.228783 13.265346 12.713020
C 24.905687 12.125906 13.469548
C 23.577994 11.907590 13.883517
C 22.572715 12.827540 13.546139
C 20.505655 15.458075 13.713406
C 19.155700 15.840684 13.519835
C 18.334966 16.108201 14.626677
C 18.850808 16.006534 15.932019
C 20.193194 15.639103 16.128970
C 21.022858 15.369613 15.027265
H 20.845347 17.152177 8.212078
H 20.813627 18.998601 6.511400
H 22.237140 21.063798 6.924178
H 23.594660 21.192929 9.049971
H 23.927432 20.256873 11.405587
H 24.627695 18.662782 10.964911
H 19.689490 18.345360 11.534258
H 17.867510 19.997331 11.951535
H 18.336857 22.090993 13.255649
H 20.646513 22.525044 14.141326
H 22.474499 20.882855 13.740715
H 21.840662 18.482401 15.111806
H 23.157321 18.081139 17.185431
H 25.578171 17.425873 17.042233
H 26.671117 17.164232 14.794801
H 25.377290 17.555654 12.717546
H 23.590086 16.143710 8.346901

H 23.748897 14.230726 6.729328
H 22.290903 12.183754 7.111447
H 20.772963 12.138503 9.129565
H 20.271987 13.165202 11.419108
H 19.602086 14.735586 10.861980
H 24.491132 15.076590 11.770654
H 26.265395 13.437997 12.384958
H 25.691068 11.402165 13.738012
H 23.320849 11.013820 14.472856
H 21.537677 12.644423 13.874876
H 18.729374 15.925596 12.508599
H 17.284434 16.395309 14.466089
H 18.203119 16.214419 16.797895
H 20.602925 15.559481 17.147637
H 22.070518 15.080192 15.196277

73
$[Ni(I)]^{+}@CH_3CN$
Ni 22.123430 16.692886 10.914226
P 22.920266 18.357802 12.177203
P 21.223875 15.091453 12.188634
N 21.836093 18.117026 9.523559
N 22.503826 15.205038 9.613363
C 20.962549 17.904495 8.506415
C 20.600486 18.905845 7.598705
C 21.174233 20.181106 7.738760
C 22.094094 20.399476 8.776382
C 22.415550 19.347902 9.653447
C 23.420420 19.499710 10.769525
C 21.753301 19.411810 13.148170
C 20.416525 18.980292 13.286531
C 19.478095 19.778706 13.966523
C 19.868641 21.013777 14.511761
C 21.200494 21.451957 14.376564
C 22.139134 20.657755 13.697472
C 24.436443 18.236761 13.218169
C 24.373259 18.330431 14.629108
C 25.523327 18.120220 15.410272

C 26.751492 17.810344 14.799199
C 26.821056 17.699580 13.397228
C 25.673207 17.900339 12.613266
C 23.448050 15.370088 8.652066
C 23.868522 14.328368 7.818007
C 23.279963 13.061765 7.974011
C 22.288042 12.891884 8.952561
C 21.910735 13.982321 9.757124
C 20.828166 13.882071 10.804359
C 22.305867 14.086081 13.299430
C 23.627218 14.527571 13.526105
C 24.506099 13.765071 14.317693
C 24.071081 12.555874 14.886816
C 22.754437 12.107711 14.663784
C 21.875267 12.866291 13.873484
C 19.630943 15.265350 13.099651
C 18.448370 15.578167 12.383756
C 17.243396 15.817868 13.064259
C 17.200916 15.770567 14.470909
C 18.374710 15.485147 15.190975
C 19.581590 15.236037 14.513857
H 20.541664 16.889296 8.435246
H 19.877653 18.684238 6.799885
H 20.911950 20.995827 7.046550
H 22.571638 21.381487 8.910642
H 23.526843 20.555584 11.088694
H 24.418544 19.160115 10.414863
H 20.114191 18.012446 12.856384
H 18.437895 19.431709 14.068407
H 19.134624 21.639625 15.043747
H 21.509696 22.419982 14.801751
H 23.178598 21.011129 13.600626
H 23.420411 18.573851 15.123947
H 25.456474 18.203194 16.506758
H 27.652111 17.651352 15.412773
H 27.776486 17.451995 12.908082
H 25.748179 17.787816 11.519367
H 23.877649 16.380430 8.565268

H 24.647142 14.512544 7.063317
H 23.586451 12.216296 7.339210
H 21.797403 11.917704 9.096202
H 20.697720 12.842402 11.165372
H 19.858349 14.200752 10.362677
H 23.964204 15.474882 13.076444
H 25.534784 14.119523 14.487902
H 24.758457 11.958020 15.506140
H 22.410610 11.159822 15.107356
H 20.846974 12.505641 13.708503
H 18.461078 15.641978 11.283379
H 16.331501 16.046234 12.490064
H 16.255594 15.960047 15.003199
H 18.353977 15.452143 16.291933
H 20.490770 15.012983 15.093185

73
$[Ni(0)]^0$@CH_3CN
Ni 22.111949 16.665595 11.005494
P 23.274246 18.242204 11.944501
P 20.885973 15.187822 12.020011
N 21.184411 18.056536 9.963447
N 23.094765 15.182697 10.161141
C 19.977525 17.858027 9.352156
C 19.233570 18.881924 8.757779
C 19.743288 20.193058 8.766717
C 20.996965 20.408469 9.368259
C 21.697921 19.336492 9.943916
C 23.067191 19.506619 10.552318
C 22.574465 19.202927 13.389290
C 21.267644 18.873388 13.808590
C 20.643024 19.588246 14.847063
C 21.324436 20.640943 15.484397
C 22.628704 20.978714 15.073348
C 23.248787 20.268040 14.030328
C 25.094449 18.304732 12.299297
C 25.544831 17.821165 13.556076
C 26.914260 17.706703 13.842922

C 27.874994 18.061772 12.875509
C 27.446147 18.535000 11.622917
C 26.073110 18.656379 11.337281
C 24.335605 15.324550 9.604414
C 25.109693 14.250057 9.155728
C 24.596318 12.944600 9.261579
C 23.309351 12.784964 9.807643
C 22.579452 13.905539 10.235996
C 21.176630 13.790403 10.777705
C 21.476290 14.373235 13.597512
C 22.750082 14.745271 14.078177
C 23.294426 14.134437 15.222900
C 22.564562 13.144658 15.905656
C 21.292720 12.764865 15.434032
C 20.753238 13.371377 14.285808
C 19.045136 15.165092 12.252217
C 18.132321 14.744109 11.253729
C 16.744323 14.899716 11.428616
C 16.233843 15.477760 12.604310
C 17.128227 15.903387 13.606215
C 18.512954 15.754479 13.429408
H 19.600533 16.823989 9.365794
H 18.262336 18.646169 8.296438
H 19.183935 21.026106 8.314445
H 21.446558 21.413346 9.387091
H 23.258508 20.555824 10.858722
H 23.826473 19.238460 9.783702
H 20.751714 18.041352 13.297013
H 19.621794 19.319141 15.162028
H 20.840931 21.199681 16.301799
H 23.166144 21.804067 15.568133
H 24.268725 20.543549 13.715963
H 24.808420 17.536242 14.325977
H 27.234595 17.336760 14.830411
H 28.949827 17.970469 13.098372
H 28.185550 18.821832 10.857647
H 25.774014 19.039490 10.349242
H 24.714071 16.356163 9.540129

H 26.106692 14.442718 8.730792
H 25.178304 12.073259 8.924752
H 22.856448 11.785382 9.897030
H 20.967954 12.776638 11.177832
H 20.463691 13.973132 9.942649
H 23.306352 15.525714 13.529305
H 24.290859 14.435670 15.584707
H 22.985021 12.667589 16.805501
H 20.717660 11.988131 15.963978
H 19.758030 13.063768 13.925323
H 18.495181 14.278316 10.324147
H 16.057469 14.556950 10.637817
H 15.147257 15.595798 12.740854
H 16.743658 16.356065 14.534557
H 19.196263 16.096272 14.224645

73
$[Ni(II)]^{2+}$@CH_3CN(M06-L)
Ni 22.103730 16.692683 10.848962
P 22.459752 18.303387 12.273154
P 21.659420 15.127521 12.295823
N 22.291593 18.157270 9.534083
N 22.021410 15.194809 9.561629
C 21.606078 18.155424 8.377351
C 21.667572 19.212931 7.482110
C 22.466368 20.309826 7.797345
C 23.161601 20.320991 9.003194
C 23.055573 19.229686 9.861134
C 23.693598 19.168706 11.209388
C 21.096056 19.476314 12.463951
C 19.859735 19.202125 11.863413
C 18.815963 20.120256 11.964257
C 19.003532 21.315435 12.656856
C 20.237503 21.599856 13.245068
C 21.284291 20.687210 13.149740
C 23.207499 17.986844 13.884148
C 22.579994 18.354388 15.082141
C 23.165786 18.024626 16.303115

C 24.362824 17.311588 16.339111
C 24.983902 16.927513 15.148483
C 24.412553 17.264413 13.925387
C 22.766251 15.192687 8.441513
C 22.781344 14.117138 7.566325
C 21.999259 13.003288 7.862769
C 21.242641 12.996045 9.030814
C 21.271578 14.106595 9.869997
C 20.547889 14.175576 11.173682
C 23.043341 14.019720 12.659294
C 24.277965 14.240479 12.032497
C 25.344608 13.374362 12.264482
C 25.182899 12.283743 13.117763
C 23.950874 12.049210 13.731196
C 22.880223 12.909384 13.503078
C 20.764173 15.482245 13.820970
C 19.474616 16.035183 13.734593
C 18.788495 16.389796 14.891906
C 19.378002 16.195679 16.142964
C 20.660109 15.657146 16.233406
C 21.358732 15.309565 15.078897
H 20.981821 17.280844 8.181069
H 21.090271 19.173023 6.558856
H 22.536936 21.158664 7.115355
H 23.780249 21.171407 9.290859
H 23.964659 20.161182 11.595283
H 24.613769 18.562365 11.186560
H 19.712192 18.272429 11.307125
H 17.854636 19.900052 11.496428
H 18.184799 22.033591 12.736223
H 20.387192 22.539175 13.780750
H 22.247963 20.919008 13.611738
H 21.623934 18.883420 15.069004
H 22.670514 18.314729 17.231974
H 24.813156 17.048381 17.298355
H 25.919239 16.364488 15.170533
H 24.911708 16.961240 13.000952
H 23.376690 16.079735 8.259272

H 23.404497 14.156740 6.673356
H 21.988195 12.139523 7.196191
H 20.633632 12.133979 9.303578
H 20.295728 13.182705 11.571571
H 19.604621 14.735998 11.068829
H 24.405870 15.090300 11.355548
H 26.304020 13.554328 11.776030
H 26.020326 11.608011 13.303321
H 23.821141 11.189379 14.391154
H 21.918313 12.714103 13.984885
H 18.998090 16.191375 12.762994
H 17.786529 16.816447 14.815670
H 18.835743 16.471109 17.049725
H 21.131518 15.517253 17.208406
H 22.375507 14.917375 15.163614

73
$[Ni(I)]^+$@CH_3CN(M06-L)
Ni 22.120056 16.694625 10.782131
P 22.884535 18.295386 12.139484
P 21.258999 15.154083 12.152384
N 22.046069 18.199467 9.403700
N 22.304725 15.129753 9.487091
C 21.270705 18.068262 8.313532
C 21.017404 19.115789 7.438894
C 21.597982 20.355062 7.702705
C 22.412430 20.493043 8.823211
C 22.629035 19.395784 9.657312
C 23.518902 19.473236 10.862159
C 21.573037 19.251071 12.977028
C 20.241895 18.901327 12.713761
C 19.194396 19.616712 13.294222
C 19.471184 20.685672 14.145297
C 20.795587 21.044339 14.409854
C 21.842662 20.334090 13.827371
C 24.204323 18.103041 13.368766
C 23.873905 17.893061 14.718953
C 24.860212 17.573137 15.649660

C 26.191867 17.453231 15.250804
C 26.529899 17.651397 13.910804
C 25.546247 17.965924 12.975901
C 23.156946 15.217683 8.451049
C 23.471496 14.135734 7.640713
C 22.872186 12.907056 7.912492
C 21.979823 12.813327 8.976775
C 21.705084 13.943315 9.747677
C 20.730036 13.912973 10.886878
C 22.500475 14.245977 13.137505
C 23.852185 14.556137 12.935213
C 24.847156 13.868507 13.630273
C 24.497373 12.868691 14.536852
C 23.152417 12.550791 14.742495
C 22.157805 13.232352 14.045127
C 19.845491 15.395595 13.263321
C 18.532477 15.463809 12.768495
C 17.476522 15.816893 13.605996
C 17.711694 16.121124 14.947782
C 19.013772 16.069417 15.447106
C 20.072062 15.713075 14.614145
H 20.833440 17.078280 8.152537
H 20.372659 18.960012 6.573500
H 21.419425 21.206027 7.042801
H 22.888080 21.446916 9.057125
H 23.612649 20.504776 11.231272
H 24.533754 19.144640 10.582541
H 20.035924 18.054135 12.050381
H 18.160462 19.332032 13.085119
H 18.653156 21.244342 14.605791
H 21.012634 21.884229 15.073689
H 22.877342 20.620593 14.039522
H 22.834221 17.981303 15.047734
H 24.584390 17.418039 16.695263
H 26.964674 17.204524 15.981285
H 27.569333 17.558542 13.587947
H 25.833485 18.096285 11.928969
H 23.605269 16.201145 8.281286

H 24.176646 14.257463 6.818007
H 23.096428 12.030360 7.302073
H 21.487455 11.869003 9.214830
H 20.615392 12.898533 11.295544
H 19.736940 14.218390 10.517350
H 24.117798 15.349699 12.228053
H 25.897643 14.120089 13.466149
H 25.274335 12.332657 15.086630
H 22.877991 11.765063 15.449981
H 21.106418 12.978278 14.211267
H 18.323286 15.247028 11.717613
H 16.461230 15.854879 13.204773
H 16.882326 16.398995 15.601719
H 19.209532 16.307527 16.495151
H 21.087411 15.680258 15.020378

73
$[Ni(0)]^0$@CH_3CN(M06-L)
Ni 22.180081 16.600577 10.770248
P 23.320762 18.150308 11.760009
P 20.904708 15.235899 11.857591
N 21.135228 18.047088 9.895423
N 23.205673 15.011255 10.163388
C 19.860193 17.851146 9.481466
C 18.945497 18.879768 9.309785
C 19.358157 20.196091 9.524110
C 20.687025 20.416082 9.897240
C 21.548347 19.337886 10.071879
C 22.969140 19.489116 10.506032
C 22.577840 18.899846 13.274513
C 21.193514 18.729051 13.440194
C 20.529281 19.286574 14.531208
C 21.243948 20.008343 15.489472
C 22.620787 20.179720 15.338926
C 23.282377 19.638512 14.235284
C 25.114286 18.304899 12.062826
C 25.662293 17.559731 13.125010
C 27.035737 17.522177 13.344993

C 27.902086 18.216064 12.495548
C 27.375977 18.953107 11.435267
C 25.997271 19.001467 11.221632
C 24.515641 15.073469 9.825148
C 25.342298 13.961374 9.761300
C 24.805689 12.700981 10.028903
C 23.447354 12.618942 10.345306
C 22.674382 13.774241 10.400677
C 21.222159 13.763680 10.754390
C 21.488083 14.587043 13.479800
C 22.850504 14.766728 13.765577
C 23.404685 14.272221 14.945098
C 22.597513 13.603897 15.867448
C 21.240130 13.421941 15.595527
C 20.689878 13.901529 14.406448
C 19.093396 15.301330 12.034619
C 18.205586 14.821038 11.056615
C 16.839207 15.093601 11.133659
C 16.326510 15.848705 12.188396
C 17.195352 16.328129 13.172727
C 18.558965 16.062523 13.093206
H 19.572270 16.810314 9.307806
H 17.923760 18.645976 9.003683
H 18.667735 21.032000 9.398289
H 21.062424 21.428571 10.061105
H 23.192849 20.509130 10.854459
H 23.630971 19.274051 9.648404
H 20.647372 18.135147 12.696078
H 19.449648 19.149367 14.639131
H 20.728911 20.435447 16.353202
H 23.185803 20.744108 16.085148
H 24.359483 19.794352 14.124569
H 24.995293 17.007752 13.796369
H 27.434534 16.944331 14.182444
H 28.981118 18.182320 12.661778
H 28.043173 19.505484 10.768819
H 25.615037 19.598513 10.390237
H 24.904036 16.074803 9.617683

H 26.395008 14.087593 9.501896
H 25.425646 11.803551 9.990729
H 22.977971 11.654781 10.552559
H 20.904386 12.798216 11.177354
H 20.630118 13.937560 9.838735
H 23.464053 15.314611 13.038448
H 24.469062 14.415743 15.149711
H 23.025277 13.225212 16.798774
H 20.604912 12.897304 16.313740
H 19.627446 13.740720 14.200060
H 18.576589 14.226110 10.217979
H 16.169417 14.706162 10.361861
H 15.256669 16.061097 12.245929
H 16.807202 16.916267 14.008287
H 19.227081 16.451318 13.869049

73
$[Ni(II)]^{2+}$
Ni 22.112286 16.703321 10.834900
P 22.556247 18.325604 12.232634
P 21.574878 15.135552 12.265074
N 22.197002 18.123935 9.484405
N 22.141477 15.222864 9.548612
C 21.450836 18.058094 8.351947
C 21.437161 19.092434 7.410795
C 22.222052 20.232698 7.645622
C 22.980173 20.308648 8.824113
C 22.948652 19.239995 9.733815
C 23.679797 19.238504 11.046960
C 21.201220 19.498588 12.613960
C 19.902859 19.258594 12.112591
C 18.877418 20.191777 12.345819
C 19.142482 21.363653 13.074976
C 20.436768 21.607770 13.572269
C 21.467072 20.681718 13.344839
C 23.514415 18.059922 13.766823
C 22.898395 18.192746 15.033494
C 23.642689 17.967048 16.203880

C 24.997013 17.599387 16.122492
C 25.610522 17.453028 14.864508
C 24.875551 17.676771 13.689697
C 22.974011 15.243870 8.476477
C 23.058121 14.172551 7.581420
C 22.253285 13.042775 7.799143
C 21.405904 13.013466 8.917400
C 21.370403 14.117246 9.783994
C 20.542704 14.169923 11.037517
C 22.896734 13.978292 12.788704
C 24.228963 14.190731 12.370809
C 25.228383 13.263327 12.714156
C 24.904387 12.123802 13.470166
C 23.576349 11.905986 13.883319
C 22.571720 12.826533 13.545613
C 20.506003 15.457325 13.713422
C 19.155889 15.839402 13.520070
C 18.335349 16.106994 14.627063
C 18.851518 16.005946 15.932322
C 20.194067 15.638977 16.129043
C 21.023498 15.369378 15.027180
H 20.844611 17.151388 8.212584
H 20.812730 18.997072 6.511134
H 22.236071 21.062599 6.923034
H 23.593708 21.192676 9.048743
H 23.927163 20.257322 11.404506
H 24.626813 18.662733 10.964207
H 19.688193 18.346475 11.535565
H 17.866903 19.998628 11.953967
H 18.337413 22.092574 13.257360
H 20.647892 22.526517 14.141167
H 22.475302 20.883958 13.739392
H 21.840238 18.482918 15.111531
H 23.156765 18.081134 17.185110
H 25.577729 17.426081 17.041850
H 26.670722 17.165146 14.794263
H 25.376844 17.556964 12.717131
H 23.592106 16.144173 8.348549

H 23.751170 14.231802 6.730196
H 22.292501 12.185013 7.110838
H 20.773682 12.139258 9.128338
H 20.271814 13.165426 11.418082
H 19.603013 14.736472 10.861083
H 24.492279 15.075118 11.771815
H 26.265225 13.435676 12.386656
H 25.689297 11.399607 13.738829
H 23.318394 11.012150 14.472219
H 21.536388 12.643802 13.873615
H 18.729354 15.923960 12.508916
H 17.284719 16.393809 14.466557
H 18.204012 16.214036 16.798300
H 20.604178 15.559941 17.147608
H 22.071305 15.080395 15.196017

73
$[Ni(I)]^+$
Ni 22.123632 16.692851 10.914366
P 22.919983 18.357865 12.177269
P 21.224426 15.091422 12.188749
N 21.836747 18.117058 9.523678
N 22.503224 15.204915 9.613308
C 20.963138 17.904852 8.506538
C 20.602303 18.906147 7.598265
C 21.177365 20.180878 7.737773
C 22.097200 20.398883 8.775502
C 22.417361 19.347404 9.653146
C 23.421984 19.498746 10.769509
C 21.752063 19.412780 13.146275
C 20.414915 18.981800 13.282793
C 19.475707 19.780834 13.961005
C 19.865855 21.016015 14.506330
C 21.198078 21.453666 14.372952
C 22.137474 20.658836 13.695604
C 24.434691 18.237153 13.220394
C 24.369370 18.330541 14.631284
C 25.518250 18.120295 15.414197

C 26.747412 17.810672 14.804951
C 26.819113 17.700251 13.403045
C 25.672454 17.901053 12.617331
C 23.447414 15.369564 8.651929
C 23.866503 14.327846 7.817155
C 23.276554 13.061813 7.972546
C 22.284739 12.892389 8.951291
C 21.908886 13.982778 9.756592
C 20.826639 13.883127 10.804215
C 22.307338 14.084893 13.297770
C 23.629106 14.525816 13.523183
C 24.508714 13.762516 14.313215
C 24.074008 12.553054 14.882060
C 22.756956 12.105444 14.660266
C 21.877082 12.864836 13.871497
C 19.632776 15.265290 13.102012
C 18.449135 15.578130 12.387867
C 17.245211 15.818032 13.070183
C 17.204800 15.770902 14.476916
C 18.379664 15.485412 15.195246
C 19.585492 15.236104 14.516317
H 20.541163 16.890079 8.435861
H 19.879390 18.684871 6.799430
H 20.916101 20.995500 7.045062
H 22.575724 21.380463 8.909388
H 23.529403 20.554650 11.088224
H 24.419811 19.157827 10.415312
H 20.112965 18.013889 12.852489
H 18.435200 19.434293 14.061388
H 19.131233 21.642364 15.036901
H 21.506971 22.421800 14.798128
H 23.177171 21.011863 13.600031
H 23.415734 18.573758 15.124715
H 25.449682 18.203020 16.510604
H 27.647100 17.651591 15.419877
H 27.775303 17.452871 12.915275
H 25.749128 17.788796 11.523541
H 23.878145 16.379464 8.565682

H 24.645134 14.511621 7.062386
H 23.581903 12.216398 7.337125
H 21.793064 11.918674 9.094497
H 20.695098 12.843412 11.164683
H 19.857085 14.203302 10.363060
H 23.965818 15.473303 13.073664
H 25.537742 14.116496 14.482387
H 24.761958 11.954556 15.500135
H 22.413370 11.157323 15.103542
H 20.848533 12.504547 13.707359
H 18.460176 15.641825 11.287481
H 16.332506 16.046454 12.497292
H 16.260302 15.960596 15.010604
H 18.360602 15.452528 16.296247
H 20.495515 15.012998 15.094308

73
$[Ni(0)]^0$
Ni 22.111799 16.665667 11.006311
P 23.273685 18.242592 11.945104
P 20.886277 15.187492 12.020666
N 21.184999 18.056229 9.962819
N 23.094071 15.183097 10.160470
C 19.978986 17.857154 9.349962
C 19.235640 18.880614 8.754032
C 19.745093 20.191855 8.763074
C 20.997934 20.407822 9.366162
C 21.698352 19.336248 9.943277
C 23.066964 19.507018 10.553014
C 22.574308 19.203394 13.390160
C 21.269199 18.871492 13.812871
C 20.645259 19.586297 14.851833
C 21.325640 20.641520 15.486096
C 22.628193 20.981773 15.071577
C 23.247585 20.270991 14.028216
C 25.093905 18.304813 12.300237
C 25.543914 17.822871 13.557787
C 26.913270 17.707991 13.844922

C 27.874293 18.061090 12.877056
C 27.445805 18.532705 11.623709
C 26.072851 18.654411 11.337745
C 24.334069 15.325503 9.601968
C 25.107749 14.251380 9.151652
C 24.594811 12.945764 9.257717
C 23.308634 12.785581 9.805485
C 22.579070 13.905817 10.235368
C 21.176841 13.790023 10.778509
C 21.476378 14.372914 13.598354
C 22.749233 14.746195 14.080553
C 23.293204 14.135385 15.225498
C 22.563897 13.144249 15.906903
C 21.293011 12.763125 15.433724
C 20.753921 13.369697 14.285336
C 19.045392 15.164927 12.252948
C 18.132585 14.745475 11.253783
C 16.744603 14.901356 11.428644
C 16.234113 15.478213 12.604943
C 17.128484 15.902354 13.607505
C 18.513207 15.753110 13.430754
H 19.602208 16.823032 9.363468
H 18.265127 18.644445 8.291392
H 19.186209 21.024569 8.309613
H 21.447325 21.412786 9.385003
H 23.257414 20.556289 10.859706
H 23.827063 19.239624 9.784956
H 20.753904 18.037685 13.303540
H 19.625395 19.315238 15.169539
H 20.842672 21.200317 16.303772
H 23.164784 21.809136 15.563912
H 24.266182 20.548438 13.711189
H 24.807317 17.539555 14.328106
H 27.233305 17.339329 14.832988
H 28.949055 17.969523 13.100145
H 28.185419 18.817979 10.858067
H 25.774122 19.036148 10.349063
H 24.712175 16.357239 9.537436

H 26.104050 14.444443 8.725267
H 25.176493 12.074703 8.919646
H 22.856053 11.785861 9.894931
H 20.969157 12.776236 11.179075
H 20.463039 13.971855 9.944010
H 23.305190 15.527585 13.532704
H 24.288882 14.437658 15.588520
H 22.984050 12.667139 16.806866
H 20.718422 11.985294 15.962565
H 19.759485 13.060991 13.923657
H 18.495367 14.280678 10.323672
H 16.057779 14.559762 10.637317
H 15.147546 15.596472 12.741438
H 16.743931 16.354084 14.536316
H 19.196446 16.093702 14.226569

74
$[Ni\text{-}NH]^{2+}$
Ni 22.524851 16.402118 11.001944
P 22.190501 18.110218 12.379564
P 21.689313 14.490332 11.760532
N 23.083109 17.686990 9.642545
N 19.554872 16.218911 10.102013
C 23.161123 17.314034 8.337913
C 23.477625 18.217955 7.319588
C 23.716132 19.560274 7.660860
C 23.640112 19.945435 9.007831
C 23.330177 18.986288 9.988132
C 23.296548 19.313741 11.461494
C 20.533155 18.893684 12.483136
C 19.545516 18.263372 13.280456
C 18.245709 18.790083 13.347106
C 17.908347 19.940031 12.608468
C 18.878695 20.562012 11.803356
C 20.185654 20.044779 11.738876
C 22.877627 18.112621 14.084242
C 22.401755 18.960293 15.108948
C 23.013386 18.940322 16.374836

C 24.101148 18.083310 16.624009
C 24.577638 17.237130 15.605357
C 23.963968 17.244286 14.341690
C 18.673888 17.175182 9.722326
C 17.365687 16.817515 9.405226
C 16.996238 15.463028 9.505702
C 17.937835 14.503755 9.908760
C 19.254676 14.895055 10.205853
C 20.344161 13.920946 10.563069
C 22.840040 13.049834 11.680890
C 24.081219 13.213704 11.027307
C 24.968851 12.128866 10.915186
C 24.623703 10.878797 11.458192
C 23.389387 10.712309 12.114886
C 22.498225 11.792246 12.228930
C 20.917769 14.364320 13.428477
C 19.519807 14.353301 13.628765
C 18.990355 14.302340 14.931612
C 19.848610 14.260206 16.043527
C 21.242766 14.270208 15.850661
C 21.776191 14.325921 14.553149
H 22.951930 16.255118 8.119479
H 23.526659 17.870784 6.277539
H 23.959476 20.301774 6.884730
H 23.827900 20.986282 9.309689
H 23.047280 20.376642 11.653815
H 24.307576 19.145295 11.894623
H 19.792747 17.356083 13.855029
H 17.490509 18.295281 13.977502
H 16.887366 20.349388 12.658973
H 18.622717 21.461061 11.221293
H 20.926740 20.550785 11.102298
H 21.555910 19.639460 14.920368
H 22.638596 19.603576 17.170280
H 24.577542 18.072390 17.616957
H 25.425505 16.561114 15.797419
H 24.326331 16.570639 13.547092
H 19.052356 18.206970 9.695686

H 16.649580 17.590208 9.094068
H 15.967513 15.151936 9.269379
H 17.663959 13.442264 9.987878
H 20.912814 13.681983 9.636144
H 19.894319 12.970252 10.912046
H 24.355792 14.193799 10.605961
H 25.935535 12.265084 10.405707
H 25.320609 10.030127 11.373752
H 23.118354 9.734587 12.543275
H 21.538392 11.653407 12.751668
H 18.823311 14.380740 12.777781
H 17.898346 14.290037 15.072207
H 19.431738 14.216657 17.061843
H 21.922042 14.234008 16.716582
H 22.868975 14.328727 14.416918
H 20.543806 16.507295 10.382117

74
$[Ni\text{-}H]^+$
Ni 0.000383 0.001468 0.010867
P 0.542637 1.661256 1.225567
P -1.594493 -1.086303 1.070181
N -0.943038 1.449122 -1.305128
N 0.224309 -1.625062 -1.073276
C -1.787854 1.146197 -2.316479
C -2.390100 2.115277 -3.129567
C -2.105130 3.467877 -2.879432
C -1.215917 3.789558 -1.843344
C -0.638456 2.755977 -1.078632
C 0.397828 3.055348 -0.015318
C -0.527513 2.176556 2.642456
C -1.639403 3.027176 2.447529
C -2.455116 3.389352 3.534292
C -2.177717 2.900127 4.822686
C -1.084240 2.036936 5.020842
C -0.265684 1.672970 3.938476
C 2.249836 1.832945 1.905004
C 3.211952 0.839467 1.624998

C 4.527541 0.968379 2.105370
C 4.890528 2.092788 2.867099
C 3.934992 3.087457 3.151306
C 2.618369 2.958824 2.678702
C 1.332149 -1.826778 -1.844696
C 1.525192 -2.974577 -2.617688
C 0.543782 -3.979441 -2.603861
C -0.602087 -3.779027 -1.821604
C -0.748129 -2.594769 -1.076916
C -1.996018 -2.327001 -0.272128
C -1.044511 -2.154712 2.474911
C 0.292213 -2.060407 2.916054
C 0.766117 -2.902965 3.938060
C -0.092557 -3.850177 4.522302
C -1.426273 -3.955349 4.082256
C -1.902561 -3.113866 3.063152
C -3.235248 -0.417795 1.581201
C -4.148700 0.031316 0.596852
C -5.376829 0.602134 0.969621
C -5.706699 0.746883 2.329849
C -4.795773 0.325518 3.313903
C -3.566464 -0.248527 2.944868
H -1.985159 0.074779 -2.484515
H -3.072765 1.809324 -3.936001
H -2.564785 4.263048 -3.486387
H -0.956977 4.837144 -1.627414
H 0.228177 4.039299 0.465730
H 1.399941 3.102300 -0.497439
H -1.877729 3.421849 1.447811
H -3.311478 4.061908 3.369473
H -2.814608 3.189769 5.673220
H -0.861544 1.647080 6.026679
H 0.593487 1.005479 4.110633
H 2.915131 -0.034608 1.022807
H 5.269210 0.184754 1.884391
H 5.920285 2.194921 3.244560
H 4.216640 3.970432 3.746448
H 1.877878 3.739477 2.915094

H 2.086207 -1.028431 -1.820425
H 2.444115 -3.074427 -3.213708
H 0.667863 -4.902283 -3.190541
H -1.400224 -4.535251 -1.784600
H -2.435189 -3.262995 0.128057
H -2.770200 -1.868454 -0.926316
H 0.960605 -1.324600 2.439243
H 1.811220 -2.819108 4.275294
H 0.276978 -4.511873 5.321643
H -2.100834 -4.699153 4.534996
H -2.948942 -3.206062 2.729915
H -3.912021 -0.060477 -0.474746
H -6.079502 0.935377 0.189740
H -6.671197 1.191806 2.621015
H -5.040255 0.442635 4.381326
H -2.863702 -0.568363 3.728770
H 1.350012 0.485190 -0.379526

75
[Ni-H-NH]$^{2+}$
Ni -1.058313 0.522041 1.186155
P 0.593013 1.924792 1.238719
P -2.264134 -1.301712 0.895822
N -2.110847 2.012924 0.449304
N 0.775469 -2.095949 0.085414
C -3.450785 2.129573 0.632650
C -4.176766 3.247198 0.206548
C -3.496149 4.290345 -0.439560
C -2.107916 4.184973 -0.616589
C -1.434748 3.040350 -0.160466
C 0.058686 2.876038 -0.279295
C 0.610190 3.122879 2.631632
C -0.474501 3.135585 3.536792
C -0.500050 4.068580 4.588139
C 0.554689 4.985548 4.742257
C 1.638863 4.971655 3.844094
C 1.671906 4.043160 2.790742
C 2.335013 1.416348 0.992703

C 2.984459 0.750120 2.060629
C 4.315684 0.325651 1.922389
C 5.010497 0.558492 0.719442
C 4.369859 1.218681 -0.342768
C 3.036751 1.649292 -0.211002
C 2.056263 -2.522172 0.199315
C 2.413960 -3.768703 -0.308686
C 1.418155 -4.560118 -0.910622
C 0.100355 -4.085521 -1.003022
C -0.222532 -2.814592 -0.499872
C -1.584711 -2.197561 -0.625817
C -2.215084 -2.561874 2.236156
C -1.614750 -2.234843 3.471321
C -1.575084 -3.181611 4.510627
C -2.127342 -4.459725 4.319487
C -2.727904 -4.791066 3.089578
C -2.778680 -3.846905 2.051675
C -4.054184 -1.176391 0.461411
C -4.473185 -0.900759 -0.861294
C -5.839463 -0.739830 -1.149487
C -6.797295 -0.837330 -0.124407
C -6.384904 -1.096039 1.195255
C -5.021262 -1.263985 1.490758
H -3.948672 1.294602 1.144850
H -5.260521 3.289057 0.386117
H -4.035639 5.182427 -0.792244
H -1.536627 4.988900 -1.102714
H 0.579314 3.846602 -0.401023
H 0.303073 2.237879 -1.157129
H -1.300017 2.415745 3.420515
H -1.346626 4.073598 5.292265
H 0.535381 5.712836 5.569100
H 2.466868 5.687525 3.965361
H 2.526409 4.034520 2.095635
H 2.448403 0.560559 3.004757
H 4.812764 -0.189388 2.759045
H 6.054117 0.224096 0.612323
H 4.908532 1.406003 -1.284565

H 2.558913 2.168958 -1.054417
H 2.754901 -1.840589 0.705682
H 3.453399 -4.113458 -0.223422
H 1.669229 -5.554022 -1.310517
H -0.686420 -4.690831 -1.474304
H -2.308113 -2.973690 -0.941502
H -1.553570 -1.433504 -1.433726
H -1.176836 -1.234608 3.617820
H -1.108330 -2.916641 5.472064
H -2.092553 -5.202539 5.131901
H -3.162515 -5.791347 2.937572
H -3.265457 -4.116692 1.100946
H -3.743905 -0.806273 -1.680170
H -6.154299 -0.535678 -2.184731
H -7.866680 -0.710130 -0.354189
H -7.128775 -1.172397 2.003630
H -4.711815 -1.470076 2.527314
H -0.132526 -0.396977 1.841919
H 0.495004 -1.186969 0.540018

75

TS-[Ni-H-NH]$^{2+}$→ [Ni(II)]$^{2+}$

Ni 0.177176 -0.160050 0.280729
P 0.114498 1.766150 1.380332
P -1.621891 -1.072873 1.137327
N -0.064109 1.837693 -1.659714
N 0.302174 -1.830782 -0.732667
C 0.176828 1.813972 -2.986124
C -0.121700 2.894649 -3.828271
C -0.680015 4.049022 -3.254447
C -0.929629 4.074410 -1.873872
C -0.616602 2.937403 -1.100715
C -0.956821 2.897248 0.376130
C -0.510371 1.874629 3.104832
C -1.497043 2.805995 3.498061
C -1.875047 2.898111 4.849222
C -1.279065 2.066151 5.812210

C -0.297179 1.135656 5.423905
C 0.088733 1.039400 4.077693
C 1.787341 2.540637 1.474445
C 2.919404 1.724941 1.700264
C 4.190895 2.305879 1.842844
C 4.343417 3.700914 1.753309
C 3.219913 4.515507 1.526613
C 1.943189 3.942679 1.392407
C 1.496444 -2.280902 -1.202226
C 1.609375 -3.469424 -1.928319
C 0.452831 -4.223763 -2.182651
C -0.778517 -3.764085 -1.694250
C -0.830176 -2.560950 -0.972314
C -2.102842 -1.996540 -0.408645
C -1.254111 -2.346984 2.397275
C 0.079323 -2.564778 2.810056
C 0.369516 -3.590576 3.725983
C -0.663556 -4.400544 4.228337
C -1.991932 -4.189319 3.813350
C -2.293446 -3.167271 2.899358
C -3.096316 -0.136699 1.664886
C -3.889414 0.525372 0.694208
C -5.028533 1.243467 1.090754
C -5.384432 1.310910 2.450622
C -4.592719 0.666564 3.417171
C -3.446990 -0.048897 3.032510
H 0.634526 0.892531 -3.384437
H 0.090088 2.832529 -4.905786
H -0.919187 4.925723 -3.875909
H -1.370350 4.962546 -1.396876
H -1.992361 2.507375 0.502617
H -0.955352 3.917004 0.810181
H -1.981356 3.468665 2.766899
H -2.644266 3.627637 5.146069
H -1.579502 2.142183 6.868817
H 0.174289 0.480663 6.172705
H 0.864769 0.312841 3.790710
H 2.817742 0.629639 1.768859

H 5.065865 1.661512 2.019792
H 5.341235 4.154651 1.858130
H 3.332790 5.608221 1.451835
H 1.079116 4.602488 1.224071
H 2.382430 -1.670068 -0.980095
H 2.598495 -3.791201 -2.283991
H 0.508784 -5.163968 -2.751620
H -1.705302 -4.329311 -1.867408
H -2.865136 -2.774113 -0.204332
H -2.545408 -1.264219 -1.118339
H 0.894754 -1.939973 2.413480
H 1.409779 -3.755096 4.046252
H -0.433976 -5.202769 4.946868
H -2.802158 -4.825140 4.202414
H -3.336993 -3.014025 2.582498
H -3.632109 0.483023 -0.375287
H -5.643300 1.748154 0.329872
H -6.281789 1.870505 2.757045
H -4.864042 0.721092 4.482425
H -2.832802 -0.542944 3.799888
H 1.696912 0.261513 -0.225145
H 1.166567 0.655689 -0.758678

78

TS-$[Ni\text{-}H]^{+}{}_{+}H_3O^{+}\rightarrow$ $[Ni(II)]^{2+}$

Ni 0.061152 -0.028727 -0.015070
P 0.531185 1.662518 1.190477
P -1.505341 -1.097262 1.104063
N -0.869326 1.405030 -1.381805
N 0.170716 -1.626591 -1.161187
C -1.658413 1.073041 -2.428385
C -2.237344 2.020652 -3.282435
C -1.989459 3.381429 -3.036829
C -1.158476 3.732905 -1.963113
C -0.601185 2.718796 -1.158394
C 0.375150 3.055345 -0.050376
C -0.536754 2.182637 2.604290

C -1.702398 2.950483 2.379542
C -2.511795 3.340235 3.460554
C -2.175481 2.958597 4.771360
C -1.028723 2.176456 4.999158
C -0.213378 1.786632 3.923070
C 2.236047 1.837130 1.878491
C 3.127134 0.739667 1.822928
C 4.435608 0.856815 2.333480
C 4.863470 2.073635 2.890172
C 3.979172 3.167664 2.951352
C 2.668201 3.050997 2.459236
C 1.197462 -1.800965 -2.042055
C 1.310860 -2.919978 -2.870517
C 0.329726 -3.922313 -2.799152
C -0.739567 -3.744358 -1.911453
C -0.809221 -2.586176 -1.115583
C -1.988961 -2.333796 -0.212168
C -0.936208 -2.168696 2.497916
C 0.390917 -2.051884 2.960072
C 0.868321 -2.894395 3.980233
C 0.022552 -3.866467 4.541432
C -1.301774 -3.995395 4.080382
C -1.781508 -3.152966 3.063873
C -3.114959 -0.387268 1.657329
C -3.988291 0.169440 0.691504
C -5.205610 0.750154 1.082739
C -5.561628 0.798746 2.443481
C -4.687808 0.272364 3.410085
C -3.469382 -0.313076 3.022749
H -1.830343 -0.003905 -2.589308
H -2.874660 1.692338 -4.116668
H -2.433512 4.160123 -3.675848
H -0.930129 4.787674 -1.748024
H 0.124835 4.017291 0.439991
H 1.388481 3.180088 -0.491763
H -1.987267 3.257842 1.361388
H -3.410451 3.948483 3.273752
H -2.808810 3.269615 5.616921

H -0.759953 1.871943 6.022911
H 0.688990 1.186548 4.119067
H 2.771989 -0.221446 1.411616
H 5.115671 -0.008317 2.296177
H 5.886875 2.169987 3.284745
H 4.312796 4.122181 3.387541
H 1.985343 3.912563 2.527142
H 1.949994 -1.003323 -2.068453
H 2.168064 -2.998797 -3.554842
H 0.393503 -4.824607 -3.426009
H -1.538929 -4.495748 -1.831279
H -2.385547 -3.275159 0.218460
H -2.818707 -1.884176 -0.800718
H 1.052274 -1.299307 2.503727
H 1.906493 -2.791304 4.332814
H 0.394965 -4.529162 5.338505
H -1.966879 -4.758502 4.514385
H -2.821043 -3.265406 2.716112
H -3.727300 0.154658 -0.378589
H -5.878837 1.168030 0.317846
H -6.517717 1.252082 2.748971
H -4.952993 0.314998 4.478107
H -2.795999 -0.717597 3.792826
H 1.449021 0.426782 -0.399463
O 3.960363 0.885712 -1.265811
H 4.769623 0.321247 -1.176018
H 4.237792 1.834525 -1.335456
H 3.373048 0.773312 -0.447754

79
2-[Ni(II)]$^{2+}$
Ni 6.833315 15.398602 5.132941
P 6.474957 17.092280 3.810870
P 7.481676 13.959354 3.630100
N 6.762386 16.805055 6.559250
N 6.652513 13.762001 6.273945
C 5.219095 17.832215 4.979452
C 5.651073 16.917597 2.188531

C 7.779540 18.365169 3.622375
C 8.496931 13.002764 4.874395
C 6.267626 12.758766 2.965883
C 8.612266 14.408463 2.265712
C 5.891005 17.837519 6.326045
C 7.519155 16.798327 7.699464
C 7.571817 12.772227 6.039141
C 5.686484 13.581476 7.226020
H 4.894280 18.843601 4.663864
H 4.322895 17.174861 4.980429
C 4.296885 16.508915 2.120678
C 6.367171 17.158190 0.992225
C 9.064985 18.148114 4.163882
C 7.479385 19.596221 2.990690
H 8.892095 12.056487 4.453954
H 9.366702 13.629482 5.166544
C 4.890175 12.924569 3.226484
C 6.717236 11.627893 2.242221
C 9.919225 14.868573 2.558926
C 8.186622 14.329771 0.919048
C 5.684568 18.858605 7.263062
C 7.335958 17.803324 8.670216
C 8.567090 15.737650 7.896848
C 7.606555 11.598063 6.802931
C 5.691068 12.417147 8.019907
C 4.605488 14.612226 7.401087
H 3.718234 16.311711 3.035674
C 3.668124 16.362904 0.874197
C 5.728794 17.010470 -0.250753
H 7.420978 17.472067 1.026915
H 9.303608 17.195639 4.659560
C 10.044310 19.152638 4.071648
C 8.462676 20.594422 2.904940
H 6.481001 19.779728 2.563244
H 4.534034 13.797779 3.792625
C 3.968562 11.969308 2.762859
C 5.790150 10.678168 1.784943
H 7.789009 11.484513 2.032373

H 10.272139 14.945687 3.598584
C 10.789952 15.223701 1.516928
C 9.066103 14.686047 -0.117477
H 7.172192 13.981391 0.674765
H 4.974341 19.667083 7.039220
C 6.402592 18.826654 8.466686
H 7.950236 17.776222 9.581676
H 9.181882 15.601423 6.984880
H 8.117129 14.758574 8.155662
H 9.241141 16.021833 8.726025
H 8.361432 10.829534 6.585061
C 6.663752 11.429123 7.826603
H 4.908633 12.293896 8.782442
H 4.176474 14.920542 6.427055
H 4.983004 15.520401 7.911635
H 3.787594 14.200363 8.020868
H 2.612265 16.054277 0.832204
C 4.381427 16.614461 -0.312548
H 6.292134 17.207391 -1.175831
H 11.045616 18.976860 4.494022
C 9.744584 20.373720 3.443575
H 8.224547 21.550832 2.414115
H 2.895367 12.104709 2.967421
C 4.416669 10.848211 2.043365
H 6.144212 9.799490 1.223808
H 11.807074 15.571410 1.754484
C 10.366200 15.131440 0.177983
H 8.728744 14.613975 -1.162899
H 6.256451 19.609213 9.226463
H 6.670388 10.521791 8.449250
H 3.883849 16.501969 -1.288399
H 10.512960 21.159359 3.372148
H 3.693240 10.101026 1.681287
H 11.053074 15.407735 -0.637105

79

2-$[Ni(I)]^{+}$

Ni 6.826614 15.387980 5.193093

P 6.095219 17.059432 3.917306
P 7.844159 13.971451 3.807344
N 7.079654 16.867251 6.613025
N 6.318783 13.675360 6.227640
C 5.373194 18.069883 5.318731
C 4.759382 16.948659 2.656339
C 7.320740 18.231953 3.177865
C 8.283650 12.706639 5.116898
C 6.821050 12.980319 2.626457
C 9.407533 14.289658 2.890365
C 6.349490 18.018642 6.469760
C 7.980381 16.778892 7.639573
C 7.089712 12.559256 6.029874
C 5.224337 13.587691 7.044335
H 5.139068 19.113641 5.027767
H 4.418063 17.584916 5.617119
C 3.390890 16.889755 3.014843
C 5.111707 16.788895 1.292818
C 8.696137 17.982707 3.375419
C 6.919700 19.400117 2.487895
H 8.597360 11.731180 4.693834
H 9.144748 13.113183 5.691247
C 5.429326 13.212980 2.582215
C 7.378801 11.956342 1.825309
C 10.673967 14.243009 3.521695
C 9.336289 14.719116 1.541459
C 6.493289 19.105831 7.346745
C 8.150481 17.837172 8.552517
C 8.813271 15.532383 7.740291
C 6.793948 11.331831 6.644411
C 4.895031 12.382644 7.693790
C 4.360317 14.807622 7.194830
H 3.078241 17.004246 4.064119
C 2.403631 16.692047 2.033714
C 4.121767 16.590854 0.316319
H 6.170068 16.826685 0.989380
H 9.009489 17.070650 3.907993
C 9.658746 18.888066 2.891733

C 7.883280 20.300124 2.003376
H 5.849847 19.607999 2.324794
H 4.995223 14.014117 3.201488
C 4.604685 12.432659 1.750850
C 6.553221 11.181258 0.994104
H 8.463687 11.764239 1.847022
H 10.769160 13.919449 4.569561
C 11.836409 14.602483 2.817374
C 10.500350 15.077688 0.842248
H 8.361843 14.767371 1.029739
H 5.889831 20.011854 7.189796
C 7.401086 19.012558 8.411181
H 8.883864 17.729784 9.365284
H 9.516068 15.470472 6.881763
H 8.172506 14.629428 7.709090
H 9.408515 15.520582 8.672480
H 7.437784 10.460906 6.452840
C 5.684872 11.242066 7.497619
H 4.008244 12.350009 8.343857
H 3.836374 15.026739 6.239642
H 4.975108 15.693787 7.446595
H 3.594532 14.664078 7.979981
H 1.343485 16.659885 2.331156
C 2.763629 16.542389 0.681975
H 4.415248 16.475062 -0.739124
H 10.729317 18.683702 3.050107
C 9.253933 20.045979 2.205265
H 7.562585 21.206967 1.466375
H 3.520268 12.622996 1.722878
C 5.165127 11.417441 0.956385
H 6.995631 10.386335 0.372848
H 12.813761 14.549727 3.322767
C 11.755389 15.020535 1.476573
H 10.425078 15.403162 -0.207465
H 7.527995 19.848445 9.116073
H 5.435608 10.293075 7.996606
H 1.987828 16.389306 -0.084560
H 10.007051 20.753848 1.824161

H 4.520510 10.807715 0.303528
H 12.667930 15.300047 0.927006

79
2-[Ni(0)]^0
Ni 6.833854 15.390319 5.145406
P 5.778045 16.892908 4.002442
P 8.129194 14.107597 3.982204
N 7.467955 16.810364 6.391856
N 5.987379 13.775636 5.950803
C 5.876211 18.308245 5.246615
C 3.977656 16.976900 3.504801
C 6.568817 17.642319 2.496256
C 7.803565 12.485178 4.888878
C 7.682106 13.667404 2.231835
C 9.994104 14.094107 3.863167
C 7.005838 18.097812 6.229327
C 8.415706 16.584913 7.365269
C 6.495783 12.532218 5.646754
C 4.860306 13.835041 6.740006
H 5.935673 19.313452 4.780095
H 4.911703 18.272337 5.802472
C 3.263005 18.188958 3.351087
C 3.295197 15.758378 3.293054
C 7.802581 18.328054 2.628937
C 6.043523 17.454181 1.195568
H 7.853004 11.589015 4.236188
H 8.634566 12.389538 5.624200
C 6.436701 13.038220 1.979845
C 8.484975 14.031085 1.124760
C 10.765405 12.916560 3.714273
C 10.665688 15.334507 3.950469
C 7.514596 19.175237 6.971249
C 8.944787 17.632186 8.141113
C 8.877355 15.167785 7.553289
C 5.867995 11.345843 6.057459
C 4.205376 12.672451 7.184901
C 4.345238 15.201297 7.093389

H 3.772393 19.155736 3.494325
C 1.900076 18.177634 3.005656
C 1.934736 15.744786 2.931680
H 3.844366 14.810131 3.419324
H 8.242591 18.482893 3.628244
C 8.476137 18.826264 1.500039
C 6.724458 17.942529 0.065553
H 5.087082 16.923469 1.063067
H 5.784407 12.752013 2.821823
C 6.019588 12.763796 0.665905
C 8.060417 13.768070 -0.190660
H 9.457048 14.522141 1.291270
H 10.271376 11.935389 3.627409
C 12.169343 12.981107 3.665762
C 12.069862 15.403064 3.884531
H 10.072552 16.256069 4.077117
H 7.118936 20.186497 6.790623
C 8.506506 18.950054 7.941439
H 9.708344 17.401028 8.899770
H 9.422789 14.816074 6.650588
H 8.007923 14.488926 7.673129
H 9.545273 15.073014 8.431893
H 6.309732 10.378147 5.774142
C 4.698146 11.406307 6.834312
H 3.301520 12.773509 7.805266
H 3.984373 15.726164 6.182125
H 5.160919 15.828485 7.508533
H 3.513941 15.143884 7.823261
H 1.355245 19.129417 2.896183
C 1.233146 16.955555 2.792833
H 1.420277 14.784583 2.765641
H 9.426954 19.368731 1.628440
C 7.941651 18.633714 0.211540
H 6.296028 17.785139 -0.937756
H 5.053945 12.260797 0.494615
C 6.828638 13.130126 -0.427147
H 8.702374 14.059918 -1.037821
H 12.755971 12.054448 3.556691

C 12.825167 14.224477 3.748099
H 12.575292 16.380119 3.949027
H 8.919041 19.781460 8.533325
H 4.190322 10.487682 7.165969
H 0.165912 16.949058 2.519231
H 8.471204 19.021015 -0.673416
H 6.500130 12.918789 -1.457197
H 13.925082 14.272939 3.706959

80
2-[Ni-NH]$^{2+}$
Ni 22.399881 16.434973 10.936717
P 22.168962 18.094780 12.409438
P 21.612048 14.530221 11.770464
N 23.025063 17.760665 9.616349
N 19.386023 15.966215 9.919652
C 23.088782 17.462899 8.283731
C 23.452950 18.444523 7.344740
C 23.758391 19.741638 7.778110
C 23.709724 20.031014 9.149770
C 23.352343 19.016528 10.051375
C 23.368530 19.240050 11.543394
C 20.584853 19.006563 12.588569
C 19.506146 18.332480 13.212570
C 18.262153 18.966858 13.358060
C 18.072323 20.273250 12.869607
C 19.134323 20.943510 12.238139
C 20.386236 20.317224 12.097519
C 22.887485 17.961299 14.097391
C 22.372486 18.647730 15.218127
C 23.002535 18.519691 16.469211
C 24.147870 17.715141 16.608467
C 24.663073 17.027471 15.493256
C 24.031042 17.140677 14.244429
C 18.480502 16.864637 9.434158
C 17.155457 16.427858 9.290864
C 16.810104 15.115550 9.649591
C 17.787124 14.233679 10.139281

C 19.110719 14.676582 10.267108
C 20.244105 13.785465 10.694376
C 22.823102 13.135498 11.751880
C 24.045403 13.304034 11.065208
C 24.971107 12.247289 11.000206
C 24.682893 11.019930 11.621921
C 23.466763 10.848038 12.310256
C 22.538577 11.900135 12.378353
C 20.901923 14.478338 13.472190
C 19.510724 14.530211 13.713362
C 19.018151 14.531734 15.031377
C 19.906645 14.481233 16.119102
C 21.293247 14.429630 15.885701
C 21.790278 14.431553 14.572219
H 23.486401 18.181394 6.277369
H 24.034765 20.522414 7.053213
H 23.954437 21.033645 9.529278
H 23.234923 20.306937 11.811789
H 24.364786 18.942828 11.940115
H 19.638347 17.305715 13.590055
H 17.434456 18.433822 13.851426
H 17.094353 20.767501 12.979149
H 18.993737 21.965148 11.851930
H 21.200865 20.865547 11.601907
H 21.482391 19.287557 15.116728
H 22.596388 19.058695 17.339630
H 24.639371 17.620542 17.589498
H 25.555738 16.391109 15.598044
H 24.422447 16.582028 13.377165
H 16.402882 17.127136 8.901363
H 15.769348 14.774101 9.543785
H 17.535298 13.199943 10.413966
H 20.781579 13.439177 9.782965
H 19.839280 12.877578 11.182981
H 24.276229 14.263258 10.576292
H 25.922580 12.388085 10.464078
H 25.409326 10.193509 11.573518
H 23.238934 9.888209 12.799842

H 21.594596 11.756871 12.927963
H 18.791161 14.565463 12.881927
H 17.931109 14.566401 15.203152
H 19.518779 14.478042 17.149725
H 21.996283 14.385107 16.732003
H 22.877266 14.383172 14.405375
H 20.376410 16.293344 10.103940
C 22.781959 16.051113 7.869303
H 23.711911 15.444073 7.833055
H 22.104977 15.569369 8.604803
H 22.309663 16.009968 6.869201
C 18.964022 18.235330 9.081933
H 19.476237 18.708693 9.945193
H 19.693579 18.187191 8.246453
H 18.118691 18.875435 8.773514

80
2-[Ni-H]$^+$
Ni -0.102840 0.030712 -0.073474
P 0.602017 1.523822 1.276758
P -1.568540 -1.194076 1.012875
N -0.933235 1.734210 -1.206139
N 0.218622 -1.507191 -1.302065
C -1.901835 1.655529 -2.163193
C -2.362047 2.806098 -2.835400
C -1.823269 4.059836 -2.517279
C -0.828858 4.134990 -1.532390
C -0.402145 2.952063 -0.901240
C 0.702712 2.994090 0.133313
C -0.470471 2.086979 2.676954
C -1.399653 3.141654 2.520219
C -2.227222 3.524078 3.591459
C -2.141229 2.856987 4.826221
C -1.225303 1.800851 4.986381
C -0.397982 1.413308 3.918930
C 2.270608 1.429150 2.058759
C 3.129705 0.351500 1.755621
C 4.419687 0.292279 2.313435

C 4.860033 1.309706 3.177574
C 4.006605 2.386057 3.487630
C 2.715702 2.445226 2.937238
C 1.156948 -1.463266 -2.307140
C 1.491785 -2.618389 -3.039579
C 0.868955 -3.840441 -2.760710
C -0.121712 -3.867914 -1.771224
C -0.442580 -2.690212 -1.076218
C -1.598126 -2.696254 -0.105332
C -1.007502 -1.897910 2.632650
C 0.290833 -2.458222 2.689957
C 0.777724 -3.006101 3.888098
C -0.018391 -2.985788 5.049573
C -1.303758 -2.419183 5.002711
C -1.798790 -1.878121 3.801052
C -3.358778 -0.822755 1.273644
C -4.324806 -1.836132 1.482091
C -5.679728 -1.499004 1.641366
C -6.083394 -0.150697 1.601217
C -5.129030 0.861391 1.397890
C -3.773230 0.526292 1.229906
H -3.146674 2.706951 -3.600172
H -2.176027 4.968994 -3.028238
H -0.378317 5.098132 -1.250054
H 0.722015 3.960769 0.674938
H 1.687545 2.887135 -0.373811
H -1.485335 3.680776 1.564262
H -2.939486 4.353417 3.457775
H -2.787240 3.160653 5.664932
H -1.151969 1.273371 5.950366
H 0.317436 0.587817 4.059174
H 2.778758 -0.444299 1.079213
H 5.079989 -0.555718 2.072962
H 5.869776 1.263697 3.615274
H 4.348106 3.185427 4.164021
H 2.054001 3.287640 3.194496
H 2.251607 -2.541818 -3.831068
H 1.137350 -4.751599 -3.316698

H -0.666790 -4.794108 -1.536955
H -1.633115 -3.643703 0.469192
H -2.546032 -2.645331 -0.686266
H 0.928874 -2.464286 1.790720
H 1.786848 -3.446809 3.915927
H 0.365663 -3.410339 5.990573
H -1.931446 -2.395777 5.907688
H -2.807925 -1.439732 3.778909
H -4.024667 -2.895348 1.525285
H -6.424969 -2.294633 1.799201
H -7.146301 0.109704 1.726703
H -5.439558 1.917478 1.363049
H -3.024827 1.315914 1.059019
H 1.307596 0.437541 -0.256661
C 1.830233 -0.158683 -2.630803
H 1.098085 0.671288 -2.668614
H 2.572873 0.108332 -1.847540
H 2.361623 -0.224522 -3.599671
C -2.471092 0.299072 -2.474526
H -1.689153 -0.376325 -2.877674
H -2.865406 -0.173009 -1.550884
H -3.291076 0.366618 -3.213872

81
2-[Ni-H-NH]$^{2+}$
Ni -1.057153 0.501448 1.472722
P 0.485562 2.001337 1.235702
P -2.123576 -1.307196 0.809278
N -2.309867 1.968785 1.074579
N 1.003894 -2.347418 0.624627
C -3.556233 2.085765 1.622710
C -4.416716 3.124498 1.216400
C -3.979805 4.074316 0.285373
C -2.665705 3.995068 -0.201225
C -1.850736 2.933166 0.213814
C -0.407847 2.805726 -0.205263
C 0.666498 3.292633 2.525664
C 0.157868 3.031661 3.817500

C 0.256472 4.009910 4.822922
C 0.855293 5.250329 4.541605
C 1.360976 5.514967 3.254301
C 1.270641 4.541159 2.246435
C 2.175921 1.609074 0.640226
C 3.302154 1.853030 1.459309
C 4.588437 1.500961 1.013853
C 4.762951 0.902695 -0.246829
C 3.644236 0.649731 -1.062373
C 2.355477 0.994868 -0.622396
C 2.147116 -2.949984 1.062409
C 2.391830 -4.260789 0.626917
C 1.463109 -4.903350 -0.206321
C 0.293806 -4.238829 -0.610765
C 0.069050 -2.922812 -0.185363
C -1.124784 -2.112477 -0.595160
C -2.376136 -2.677405 2.007896
C -1.993313 -2.466255 3.351339
C -2.149245 -3.491394 4.301016
C -2.682413 -4.732769 3.913444
C -3.061698 -4.950757 2.574938
C -2.911945 -3.929577 1.622461
C -3.739844 -1.055914 -0.048023
C -3.756666 -0.327522 -1.261499
C -4.972421 -0.075437 -1.918298
C -6.183181 -0.532299 -1.365656
C -6.173522 -1.241629 -0.152009
C -4.959268 -1.503678 0.507570
H -5.421361 3.187980 1.659172
H -4.645907 4.888988 -0.037176
H -2.267076 4.748171 -0.895851
H 0.029013 3.779918 -0.503888
H -0.306366 2.115278 -1.070481
H -0.314033 2.060177 4.036309
H -0.139044 3.800823 5.829052
H 0.929440 6.017009 5.328828
H 1.829555 6.486605 3.032820
H 1.675495 4.758093 1.244971

H 3.178342 2.322518 2.447072
H 5.458875 1.698757 1.658605
H 5.771837 0.631270 -0.594688
H 3.773004 0.179986 -2.049820
H 1.493850 0.780163 -1.273989
H 3.308602 -4.770160 0.954294
H 1.649482 -5.934491 -0.542435
H -0.442947 -4.729085 -1.261744
H -1.809162 -2.751918 -1.184063
H -0.804608 -1.278432 -1.256002
H -1.566984 -1.495231 3.650402
H -1.850053 -3.317466 5.346270
H -2.803195 -5.536697 4.656453
H -3.478136 -5.923314 2.269540
H -3.220864 -4.113384 0.581191
H -2.820974 0.049936 -1.703900
H -4.971903 0.484900 -2.866239
H -7.135562 -0.332039 -1.881015
H -7.117365 -1.599119 0.288628
H -4.968018 -2.062504 1.455654
H -0.010661 -0.407542 1.941055
H 0.762229 -1.394753 0.993086
C -3.938688 1.157695 2.739007
H -5.036656 1.075457 2.842568
H -3.505647 0.147336 2.604528
H -3.532364 1.548756 3.697228
C 3.046920 -2.178362 1.976424
H 3.373696 -1.228613 1.504249
H 2.519227 -1.918059 2.918139
H 3.941879 -2.773957 2.229211

81

2-TS-[Ni-H-NH]$^{2+}$→ [Ni(II)]$^{2+}$

Ni 0.367719 -0.109617 0.353901
P 0.207954 1.800249 1.461658
P -1.527589 -0.984281 1.012513
N -0.298961 1.881078 -1.495654

N 0.599147 -1.847105 -0.576622
C -0.226844 1.841404 -2.850899
C -0.843833 2.835716 -3.637484
C -1.527269 3.886270 -3.007685
C -1.582621 3.923109 -1.607814
C -0.954789 2.890186 -0.882399
C -1.041758 2.882341 0.633641
C -0.242185 1.749075 3.239992
C -0.874870 2.848196 3.866085
C -1.149375 2.805218 5.243084
C -0.788758 1.678637 6.005156
C -0.149795 0.589000 5.388161
C 0.122125 0.621197 4.009248
C 1.809391 2.710629 1.424846
C 2.860993 2.259211 2.256715
C 4.105462 2.908577 2.233193
C 4.313917 4.007383 1.378995
C 3.272326 4.455760 0.548654
C 2.022542 3.810684 0.564951
C 1.820037 -2.419255 -0.821051
C 1.897866 -3.611996 -1.566562
C 0.736790 -4.229397 -2.044998
C -0.507186 -3.655212 -1.750211
C -0.545321 -2.463721 -1.014845
C -1.839631 -1.798755 -0.634159
C -1.402709 -2.342498 2.235011
C -0.135576 -2.815385 2.643124
C -0.043958 -3.911628 3.517064
C -1.211889 -4.539220 3.984794
C -2.475278 -4.073201 3.576117
C -2.577671 -2.978091 2.703199
C -3.011193 -0.006822 1.427886
C -3.847142 0.507355 0.405952
C -4.977279 1.269975 0.742109
C -5.280714 1.530813 2.090684
C -4.451372 1.026018 3.108411
C -3.318457 0.262906 2.784344
H -0.776123 2.784547 -4.734397

H -2.010612 4.675169 -3.604516
H -2.105915 4.733794 -1.079316
H -2.033409 2.488245 0.947932
H -0.972385 3.912854 1.038229
H -1.156793 3.744173 3.293301
H -1.648740 3.661405 5.722440
H -1.006501 1.651294 7.084175
H 0.136724 -0.294905 5.978384
H 0.616166 -0.239537 3.533722
H 2.711787 1.402600 2.932739
H 4.916035 2.553043 2.888082
H 5.290804 4.515246 1.361578
H 3.427009 5.316939 -0.119768
H 1.225131 4.180102 -0.096475
H 2.886710 -4.055623 -1.750934
H 0.798438 -5.162120 -2.625549
H -1.446656 -4.120331 -2.080096
H -2.687417 -2.510607 -0.587092
H -2.097398 -1.002735 -1.365735
H 0.782249 -2.330791 2.277159
H 0.946381 -4.272909 3.833837
H -1.138410 -5.395858 4.672733
H -3.391436 -4.564576 3.938476
H -3.571515 -2.623729 2.389241
H -3.631452 0.317802 -0.655490
H -5.625124 1.658648 -0.058297
H -6.168409 2.128603 2.349286
H -4.685849 1.225285 4.165117
H -2.680058 -0.128350 3.590692
H 1.898038 0.381280 -0.121376
H 1.365202 0.717376 -0.663663
C 3.074480 -1.788341 -0.280554
H 3.363418 -0.893724 -0.872767
H 2.965721 -1.478056 0.778262
H 3.915804 -2.503260 -0.343413
C 0.555144 0.715054 -3.476576
H 1.631685 0.793073 -3.213309
H 0.199401 -0.268526 -3.109255

H 0.472066 0.729404 -4.579331

84

2-TS-$[Ni-H]^{+}{}_{+}H_3O^{+}\rightarrow$ $[Ni(II)]^{2+}$

Ni 0.141183 -0.066341 -0.057354
P 0.848849 1.448535 1.264451
P -1.291608 -1.263113 1.138186
N -0.887319 1.764860 -1.104573
N 0.142014 -1.547476 -1.396887
C -1.946092 1.739865 -1.960318
C -2.513659 2.932310 -2.458626
C -1.975046 4.168463 -2.082090
C -0.874741 4.187698 -1.213338
C -0.354588 2.965843 -0.748144
C 0.855106 2.954367 0.163007
C -0.125915 1.992377 2.740922
C -1.283409 2.787723 2.564204
C -2.045873 3.189154 3.674718
C -1.668366 2.797278 4.971825
C -0.523438 2.001845 5.153948
C 0.243051 1.597360 4.046828
C 2.574106 1.360978 1.927050
C 3.324287 0.167592 1.768089
C 4.647671 0.085998 2.255338
C 5.231485 1.197438 2.895170
C 4.488231 2.378888 3.057203
C 3.165076 2.461284 2.582842
C 0.559954 -1.401060 -2.694134
C 0.445844 -2.461118 -3.616097
C -0.093645 -3.689453 -3.221288
C -0.504886 -3.838225 -1.889923
C -0.367161 -2.760601 -1.002018
C -0.735666 -2.905190 0.448013
C -1.121134 -1.554754 2.957044
C 0.166231 -1.820570 3.479546
C 0.336301 -2.106749 4.843887
C -0.774324 -2.111243 5.708408

C -2.054106 -1.830965 5.199148
C -2.230316 -1.557812 3.830096
C -3.115870 -1.163012 0.872369
C -3.908791 -2.261177 0.470654
C -5.290830 -2.098860 0.264189
C -5.896055 -0.845799 0.465588
C -5.113500 0.252038 0.869497
C -3.730763 0.097814 1.061607
H -3.377333 2.876634 -3.138057
H -2.408136 5.108359 -2.457897
H -0.419201 5.136141 -0.892412
H 0.928629 3.893542 0.746305
H 1.780892 2.870332 -0.448177
H -1.593959 3.113784 1.559407
H -2.937555 3.817119 3.522077
H -2.265653 3.114653 5.840932
H -0.219394 1.691893 6.165968
H 1.138254 0.978931 4.209294
H 2.849558 -0.710169 1.296643
H 5.214361 -0.851367 2.141556
H 6.263239 1.135237 3.273185
H 4.939571 3.249377 3.557859
H 2.598292 3.393936 2.730395
H 0.781940 -2.300295 -4.650936
H -0.193063 -4.518766 -3.937951
H -0.927871 -4.785717 -1.526238
H 0.180254 -3.152694 1.029118
H -1.450232 -3.733548 0.622757
H 1.048263 -1.806429 2.818665
H 1.343674 -2.321418 5.233788
H -0.640588 -2.331884 6.779102
H -2.928642 -1.830905 5.868746
H -3.240169 -1.353879 3.443411
H -3.460963 -3.254037 0.314650
H -5.897058 -2.961799 -0.053462
H -6.978739 -0.723336 0.304927
H -5.580093 1.236965 1.027797
H -3.121734 0.964981 1.363590

H 1.507613 0.338729 -0.489674
O 4.583884 0.707322 -0.811861
H 4.314814 0.558469 0.173292
H 4.931613 1.628986 -0.914411
H 3.761812 0.626366 -1.361019
C 1.168958 -0.101276 -3.139706
H 0.755226 0.754988 -2.579071
H 2.268086 -0.124398 -2.963635
H 1.026247 0.050671 -4.226963
C -2.484161 0.403345 -2.394060
H -1.904904 0.015146 -3.259671
H -2.402278 -0.338681 -1.579038
H -3.540995 0.480648 -2.713222

77
3-[Ni(II)]$^{2+}$
Ni 6.782267 15.358151 5.719654
P 6.424179 17.028160 4.373244
P 7.407751 13.913795 4.216547
N 6.715060 16.712399 7.136400
N 6.586861 13.795299 6.890152
C 5.439738 18.067274 5.565069
C 5.378866 16.760503 2.899877
C 8.147126 12.673894 5.393745
C 8.724622 14.402175 3.049165
C 6.094550 17.911449 6.908146
C 7.385808 16.518011 8.301453
C 7.242995 12.630953 6.592669
C 5.700471 13.818607 7.919230
H 5.435505 19.115367 5.200530
H 4.398152 17.680776 5.581599
C 4.063194 16.269084 3.089972
C 5.859301 16.973055 1.587178
H 8.230216 11.697070 4.873813
H 9.162689 13.028308 5.672404
C 9.963557 14.839870 3.580492
C 8.524855 14.414198 1.649606
C 6.117549 18.931045 7.874387

C 7.448243 17.497078 9.297870
C 7.034924 11.470128 7.356119
C 5.447600 12.693883 8.710762
H 3.676918 16.071981 4.102783
C 3.237380 16.020039 1.982582
C 5.025872 16.718440 0.483957
H 6.878978 17.344510 1.412758
H 10.133443 14.862432 4.668831
C 10.990747 15.258032 2.720123
C 9.558696 14.836953 0.795649
H 7.570696 14.086822 1.213070
H 5.609544 19.883388 7.665474
C 6.793611 18.721095 9.085662
H 8.007738 17.294142 10.222132
H 7.576855 10.549749 7.095851
C 6.134717 11.501511 8.431752
H 4.717259 12.761244 9.529656
H 2.214714 15.644647 2.140942
C 3.716181 16.246372 0.678322
H 5.406780 16.894366 -0.533802
H 11.951632 15.589935 3.142529
C 10.791064 15.255365 1.326673
H 9.395929 14.834944 -0.293092
H 6.819291 19.511527 9.850992
H 5.962009 10.599111 9.037560
H 3.066058 16.051329 -0.188673
H 11.598865 15.582813 0.653907
C 6.045993 13.040044 3.309755
H 5.457061 13.813968 2.777277
H 5.387620 12.668546 4.126723
C 7.920417 18.026308 3.917227
H 8.625850 17.344866 3.400349
H 8.387588 18.253681 4.901572
C 6.428470 11.898663 2.381785
C 5.846009 11.802821 1.098813
N 7.299957 10.978564 2.846879
C 6.179238 10.709528 0.283488
H 5.139478 12.571332 0.749358

C 7.618384 9.937575 2.055889
C 7.085637 9.753067 0.768400
H 5.736271 10.609374 -0.719684
H 7.378209 8.881207 0.164052
C 7.708797 19.307631 3.127823
C 8.541161 19.607477 2.027048
N 6.737433 20.147143 3.545107
C 8.356714 20.823178 1.349480
H 9.323548 18.900087 1.711770
C 6.563759 21.307171 2.885647
C 7.346394 21.695729 1.784722
H 8.994793 21.082694 0.490339
H 7.163598 22.658011 1.283149
H 8.337891 9.209470 2.471174
H 5.756203 21.964155 3.255141
H 7.890511 15.548574 8.422430
H 5.178096 14.769671 8.097349

77
3-[Ni(I)]$^{+}$
Ni 7.116305 15.391634 5.085621
P 6.377353 17.145761 3.971252
P 8.439094 14.239015 3.726641
N 7.056762 16.543942 6.718580
N 6.603302 13.612951 5.856564
C 6.690625 18.480181 5.237242
C 4.566007 17.197097 3.626804
C 8.630334 12.723950 4.820709
C 10.168138 14.774663 3.370318
C 6.817338 17.889125 6.620994
C 7.259496 16.008188 7.952867
C 7.329933 12.493777 5.553659
C 5.441348 13.477534 6.546153
H 7.657291 18.941150 4.929522
H 5.951555 19.306821 5.208587
C 3.644673 17.726499 4.561702
C 4.063434 16.571624 2.458163
H 8.972201 11.819799 4.278823

H 9.426374 12.994894 5.549740
C 10.655236 15.882812 4.101758
C 11.004568 14.144359 2.419885
C 6.742694 18.700299 7.769027
C 7.218104 16.762660 9.128801
C 6.879009 11.213709 5.921562
C 4.936167 12.233460 6.940543
H 3.998689 18.203245 5.488854
C 2.260697 17.649806 4.323255
C 2.680977 16.505003 2.219925
H 4.753529 16.126014 1.723153
H 9.992508 16.386248 4.826244
C 11.966751 16.347103 3.898382
C 12.313028 14.618602 2.216729
H 10.624127 13.275778 1.852564
H 6.537369 19.775274 7.654608
C 6.944143 18.138409 9.038334
H 7.392429 16.271850 10.097640
H 7.482087 10.331767 5.659364
C 5.668863 11.077610 6.621066
H 3.983415 12.178710 7.487334
H 1.559592 18.071585 5.060945
C 1.773383 17.044305 3.151431
H 2.310502 16.024251 1.300606
H 12.337198 17.210894 4.472981
C 12.797007 15.715725 2.954334
H 12.962135 14.124016 1.476157
H 6.890950 18.765019 9.941717
H 5.303961 10.081109 6.913602
H 0.689349 16.988521 2.965356
H 13.822592 16.082729 2.789260
C 7.184583 17.812918 2.411939
H 6.891805 17.131066 1.588487
H 8.271669 17.678427 2.586838
C 7.744672 13.586740 2.089661
H 8.247690 14.180970 1.298813
H 6.678345 13.885942 2.102317
C 6.891748 19.256159 2.082931

C 5.859347 19.619855 1.187655
N 7.665600 20.181436 2.699350
C 5.630087 20.979686 0.927992
H 5.252056 18.843975 0.697080
C 7.434466 21.482271 2.444131
C 6.433433 21.938066 1.568315
H 4.835537 21.286656 0.229570
H 6.293235 23.015538 1.392773
C 7.863402 12.108983 1.798068
C 6.735234 11.262543 1.900350
N 9.077621 11.633409 1.436133
C 6.875049 9.896238 1.610395
H 5.758567 11.676713 2.194771
C 9.202366 10.322763 1.159687
C 8.135749 9.409368 1.228684
H 6.007436 9.221242 1.679519
H 8.294336 8.346875 0.989731
H 7.460651 14.926564 7.981204
H 4.904188 14.411924 6.774574
H 8.087961 22.206457 2.964255
H 10.210629 9.978804 0.865757

77
3-[Ni(0)]^{0}
Ni 6.809759 15.387771 5.090735
P 5.644821 16.907190 4.087118
P 8.183480 14.007891 4.151868
N 7.624446 16.803874 6.176287
N 5.805176 13.844013 5.766102
C 5.795100 18.244583 5.401765
C 3.826570 16.897283 3.715207
C 7.782559 12.502713 5.207286
C 10.038835 14.041842 4.171239
C 7.110902 18.084339 6.125014
C 8.778372 16.622524 6.889870
C 6.340667 12.576966 5.649404
C 4.522590 13.938794 6.234623
H 5.650674 19.286340 5.045753

H 4.966680 18.026336 6.114334
C 2.905600 17.892597 4.118766
C 3.332572 15.762686 3.026637
H 8.017621 11.518727 4.749515
H 8.437535 12.609991 6.102375
C 10.869378 12.982701 4.607194
C 10.649395 15.255894 3.773023
C 7.761925 19.169081 6.734394
C 9.468329 17.660335 7.523186
C 5.594485 11.426233 5.950920
C 3.732461 12.831709 6.557486
H 3.252217 18.792058 4.650938
C 1.533326 17.756106 3.837566
C 1.965576 15.634428 2.728882
H 4.036418 14.964940 2.732796
H 10.431982 12.021372 4.918466
C 12.268102 13.135114 4.639643
C 12.046585 15.402796 3.787396
H 10.011089 16.099448 3.458207
H 7.314800 20.172330 6.656140
C 8.961021 18.970347 7.444024
H 10.396478 17.436059 8.071063
H 6.065964 10.438172 5.832753
C 4.268605 11.539281 6.409219
H 2.705019 12.989083 6.920128
H 0.830299 18.540596 4.161777
C 1.059249 16.631409 3.137919
H 1.603319 14.746029 2.186720
H 12.899129 12.299839 4.984470
C 12.861677 14.341559 4.225631
H 12.501433 16.354251 3.467618
H 9.479708 19.814055 7.924088
H 3.674984 10.643566 6.647238
H -0.015140 16.528747 2.916750
H 13.957064 14.456966 4.249648
C 7.825838 13.396650 2.368961
H 8.109209 14.250560 1.720790
H 6.721579 13.292525 2.344590

C 6.354665 17.753343 2.518422
H 6.218480 17.003086 1.713331
H 7.439419 17.839058 2.734640
C 8.514459 12.146810 1.904632
C 7.927044 10.869008 2.088617
N 9.728911 12.291766 1.317016
C 8.617929 9.725793 1.661318
H 6.928370 10.781636 2.544923
C 10.380935 11.186445 0.913610
C 9.879625 9.880704 1.060593
H 8.173433 8.725698 1.788892
H 10.459046 9.014541 0.705906
C 5.764093 19.068294 2.101464
C 6.287721 20.296727 2.579443
N 4.699674 19.029512 1.260876
C 5.689602 21.501192 2.181587
H 7.168585 20.300603 3.240726
C 4.134940 20.192415 0.888890
C 4.582135 21.455346 1.316594
H 6.088016 22.465253 2.536414
H 4.080829 22.373319 0.973392
H 4.129019 14.961894 6.334463
H 9.153613 15.588489 6.929433
H 3.273031 20.118158 0.199244
H 11.365383 11.347503 0.435128

77
Masuda-$[Ni(II)]^{2+}$
Ni 21.374519 16.354159 10.875999
P 22.304656 17.988061 12.080301
P 21.429258 14.512385 9.620611
N 21.645608 17.571905 9.409506
N 21.244380 15.103824 12.333491
C 20.808907 17.625093 8.325499
C 21.148419 18.419607 7.197267
C 22.305639 19.189400 7.225370
C 23.121942 19.188509 8.377046
C 22.767192 18.371179 9.448998

C 23.558001 18.304515 10.728560
C 21.460832 19.596812 12.338158
C 20.164126 19.793116 11.813664
C 19.527451 21.038396 11.954834
C 20.180379 22.091626 12.618583
C 21.474785 21.902087 13.138137
C 22.117954 20.661257 12.999773
C 23.226716 17.591461 13.607538
C 22.694833 17.946771 14.870070
C 23.372951 17.587494 16.046180
C 24.578992 16.866641 15.977012
C 25.105251 16.499684 14.725373
C 24.432587 16.853225 13.543911
C 20.563001 15.375337 13.491386
C 20.633074 14.477102 14.590938
C 21.352525 13.294602 14.465571
C 21.993267 12.989266 13.245447
C 21.922549 13.911007 12.202480
C 22.557867 13.678046 10.857282
C 19.971732 13.408269 9.479653
C 18.758947 13.782518 10.099508
C 17.646616 12.924684 10.047718
C 17.739468 11.691987 9.378292
C 18.947825 11.312495 8.764032
C 20.064164 12.163380 8.812952
C 22.285843 14.527886 8.006910
C 23.681268 14.757853 7.945720
C 24.329240 14.829685 6.701657
C 23.594042 14.686492 5.511040
C 22.204502 14.474872 5.566213
C 21.548595 14.399702 6.805897
H 20.469806 18.434774 6.332473
H 22.568662 19.817325 6.360643
H 24.023587 19.812630 8.441619
H 24.156854 19.220557 10.901891
H 24.253886 17.437763 10.708535
H 19.649755 18.977500 11.282272
H 18.516124 21.182565 11.544312

H 19.679381 23.065709 12.732290
H 21.989892 22.726281 13.655731
H 23.130524 20.524633 13.410462
H 21.749115 18.505280 14.936315
H 22.954046 17.874541 17.023163
H 25.109074 16.588061 16.901150
H 26.047441 15.933434 14.663948
H 24.860601 16.550027 12.576456
H 20.088255 14.721824 15.513936
H 21.400268 12.588420 15.308345
H 22.542717 12.048436 13.105664
H 22.710614 12.601339 10.644671
H 23.547184 14.182041 10.803121
H 18.682351 14.741581 10.635616
H 16.704565 13.224114 10.532420
H 16.866505 11.022076 9.334568
H 19.023874 10.345873 8.242136
H 21.004333 11.854909 8.329120
H 24.275652 14.880781 8.863851
H 25.416464 14.999271 6.665576
H 24.105214 14.742996 4.537314
H 21.623092 14.366649 4.637459
H 20.460343 14.240214 6.836871
N 19.857687 16.541305 13.574030
H 19.479846 16.955083 12.719181
H 19.284294 16.689500 14.407127
N 19.674522 16.865492 8.346993
H 19.244188 16.652911 9.249949
H 19.012416 16.980690 7.576678

36

1-ligand

P 0.430464 -0.358907 0.058577
N 0.628416 -0.584129 -3.298538
C -0.006182 -0.453512 -4.477558
C -0.421422 0.780420 -5.009849
C -0.158308 1.941476 -4.264543
C 0.503138 1.819162 -3.032304

C 0.886255 0.535193 -2.581430
C 1.594338 0.346906 -1.260748
C -0.661183 1.116807 0.382113
C -2.014118 1.031760 -0.017950
C -2.889233 2.117477 0.175159
C -2.421706 3.298540 0.777577
C -1.076442 3.392172 1.184940
C -0.201980 2.309668 0.988632
C 1.517784 -0.345154 1.565153
C 0.897431 -0.615914 2.811214
C 1.654457 -0.703054 3.990289
C 3.052836 -0.539057 3.947397
C 3.681691 -0.283844 2.717071
C 2.922241 -0.184917 1.534793
H -0.195679 -1.388185 -5.037692
H -0.935201 0.825401 -5.982355
H -0.462242 2.932264 -4.638146
H 0.729066 2.708333 -2.423608
H 2.050635 1.294008 -0.906984
H 2.392873 -0.413742 -1.389478
H -2.386090 0.103542 -0.482739
H -3.941286 2.036641 -0.141977
H -3.105973 4.147618 0.934405
H -0.706943 4.315306 1.659919
H 0.847438 2.391307 1.316131
H -0.195520 -0.758395 2.858777
H 1.150356 -0.906518 4.948764
H 3.648734 -0.613077 4.870870
H 4.775048 -0.154480 2.671373
H 3.443684 0.020396 0.587669

37

diss-[Ni(II)]$^{2+}$

Ni 22.257069 16.293743 11.103301
P 21.571105 14.669997 12.290199
N 22.084024 15.183185 9.668745
C 22.720387 15.586878 8.536741
C 22.641593 14.841481 7.358603

C 21.900113 13.647456 7.358250
C 21.252174 13.238493 8.533812
C 21.347192 14.027101 9.689466
C 20.661683 13.710063 10.989832
C 22.916712 13.658453 12.981127
C 24.204623 13.722360 12.401321
C 25.226494 12.891144 12.887070
C 24.969131 12.002668 13.946686
C 23.687615 11.941842 14.525661
C 22.656663 12.767998 14.051534
C 20.492015 15.240155 13.618695
C 19.088145 15.347917 13.452365
C 18.304099 15.863903 14.493377
C 18.908723 16.297496 15.689249
C 20.304702 16.213410 15.849651
C 21.100276 15.691505 14.820638
H 23.295868 16.525181 8.599003
H 23.165720 15.198235 6.460801
H 21.828703 13.035675 6.446705
H 20.662918 12.311050 8.566466
H 20.643598 12.625959 11.218639
H 19.609898 14.067977 10.959507
H 24.408738 14.422781 11.577426
H 26.229947 12.943844 12.437797
H 25.773855 11.354965 14.327901
H 23.486037 11.246339 15.354887
H 21.656898 12.713112 14.508697
H 18.597614 15.020950 12.523822
H 17.212606 15.929213 14.369019
H 18.286913 16.707221 16.500211
H 20.777848 16.556828 16.782049
H 22.191567 15.630142 14.952541

37

diss-$[Ni(I)]^+$

Ni 22.261290 16.411848 10.963510
P 21.281057 14.990922 12.218257
N 22.548693 15.102725 9.616289

C 23.418013 15.308816 8.589079
C 23.735955 14.311395 7.663091
C 23.139936 13.045484 7.802039
C 22.236032 12.834332 8.856323
C 21.942135 13.880361 9.746986
C 20.927474 13.738370 10.859120
C 22.312634 14.096758 13.455933
C 23.469152 14.752426 13.934576
C 24.312868 14.120188 14.864757
C 24.013066 12.822426 15.316298
C 22.866720 12.159090 14.839018
C 22.017894 12.791319 13.914068
C 19.635089 15.248938 13.010487
C 18.639750 15.892053 12.235032
C 17.373717 16.158037 12.780015
C 17.092067 15.808315 14.114928
C 18.083798 15.193040 14.898556
C 19.350397 14.913555 14.352300
H 23.868514 16.312731 8.528564
H 24.446649 14.527583 6.851959
H 23.373755 12.232730 7.097790
H 21.744283 11.859240 8.990586
H 20.874477 12.695023 11.230364
H 19.916206 13.992110 10.470879
H 23.705295 15.763938 13.564399
H 25.210792 14.641384 15.232452
H 24.676087 12.323213 16.040539
H 22.631119 11.141476 15.188665
H 21.122167 12.259785 13.554270
H 18.862962 16.196441 11.198168
H 16.605931 16.650388 12.162388
H 16.101060 16.022871 14.545027
H 17.873066 14.923822 15.945782
H 20.116271 14.428988 14.977027

37
diss-[Ni(0)]^0
Ni 22.164729 16.586511 11.586405

P 20.972659 15.110352 12.326097
N 23.118327 15.647157 10.313316
C 24.346053 15.966596 9.791025
C 25.196308 15.023371 9.199725
C 24.766234 13.690061 9.085966
C 23.484840 13.357972 9.583241
C 22.684691 14.332634 10.193076
C 21.312507 14.034824 10.761056
C 21.409226 13.971332 13.721221
C 22.467699 13.034869 13.605235
C 22.901019 12.293108 14.719816
C 22.287216 12.467003 15.973639
C 21.236116 13.398519 16.104109
C 20.810286 14.146846 14.995171
C 19.109860 15.095117 12.358172
C 18.314080 13.968827 12.674347
C 16.910423 14.060028 12.653610
C 16.282555 15.271916 12.305873
C 17.065161 16.396779 11.982051
C 18.467868 16.306378 12.015042
H 24.634118 17.027353 9.865793
H 26.181996 15.341848 8.825575
H 25.403146 12.923455 8.618967
H 23.103121 12.328102 9.501599
H 21.155972 12.944179 10.906552
H 20.533004 14.387470 10.048363
H 22.964074 12.881647 12.633319
H 23.723424 11.568156 14.603874
H 22.624872 11.883577 16.844936
H 20.745850 13.544027 17.080700
H 19.995786 14.880435 15.117734
H 18.793748 13.013065 12.941770
H 16.300667 13.176953 12.905746
H 15.182895 15.339569 12.290418
H 16.580618 17.349088 11.711834
H 19.099935 17.182699 11.783516

39

2-ligand
P -0.518546 -0.693084 -0.180054
N -0.664259 -1.221170 3.136992
C -1.755873 -0.251660 1.185915
C -1.602413 -0.680534 -1.689185
C 0.386319 0.925786 -0.366011
C -1.069829 -0.088208 2.522676
C -0.037768 -1.141009 4.332241
H -2.329408 0.656975 0.910294
H -2.451871 -1.114675 1.245709
C -3.015354 -0.723232 -1.654321
C -0.958014 -0.738497 -2.951040
C 1.741867 0.968788 0.031834
C -0.216039 2.104047 -0.866573
C -0.855773 1.190770 3.084031
C 0.209647 0.099543 4.961924
C 0.381017 -2.448664 4.964538
H -3.554452 -0.686656 -0.695570
C -3.760703 -0.811949 -2.846306
C -1.702617 -0.815783 -4.138703
H 0.143745 -0.720082 -3.004162
H 2.225205 0.054414 0.414327
C 2.479258 2.164545 -0.059683
C 0.521041 3.296791 -0.961807
H -1.269523 2.088006 -1.190732
H -1.198362 2.094342 2.556925
C -0.206715 1.278765 4.324734
H 0.720289 0.134625 5.936792
H 1.040278 -3.017995 4.276078
H -0.506474 -3.088554 5.155830
H 0.915729 -2.294569 5.921433
H -4.861099 -0.842360 -2.796537
C -3.109874 -0.854852 -4.090863
H -1.180805 -0.851647 -5.108576
H 3.535172 2.182729 0.254420
C 1.870148 3.329615 -0.557633
H 0.040662 4.207360 -1.354777
H -0.027678 2.259966 4.792738

H -3.695121 -0.921056 -5.021716
H 2.446674 4.265224 -0.635169

40
2-*diss*-$[Ni(II)]^{2+}$
Ni 5.434868 13.962462 4.677403
P 6.848341 12.837355 3.560062
N 6.495273 13.636634 6.141045
C 7.470368 11.762849 4.930122
C 6.000359 11.880606 2.279598
C 8.159853 13.856229 2.842514
C 7.389997 12.598068 6.179185
C 6.322928 14.476483 7.207614
H 6.767192 10.902994 4.988153
H 8.477949 11.342767 4.739562
C 4.812874 11.193944 2.642346
C 6.491959 11.809047 0.953115
C 9.517684 13.708784 3.210637
C 7.777297 14.860544 1.916136
C 8.153551 12.362796 7.328839
C 7.064123 14.254871 8.379824
C 5.350128 15.612142 7.049921
H 4.415246 11.266722 3.666957
C 4.138715 10.423268 1.685472
C 5.805968 11.033177 0.006684
H 7.411599 12.340646 0.667345
H 9.833314 12.942167 3.933375
C 10.485224 14.547513 2.634349
C 8.754129 15.691062 1.349132
H 6.719025 14.996569 1.642368
H 8.870849 11.530171 7.340266
C 7.981379 13.196042 8.444403
H 6.916471 14.931677 9.233722
H 4.313532 15.246043 6.886731
H 5.632525 16.259412 6.192207
H 5.336658 16.241787 7.958505
H 3.219089 9.887854 1.966204
C 4.633664 10.343174 0.369494

H 6.192786 10.965002 -1.021519
H 11.542836 14.425087 2.913457
C 10.107005 15.534820 1.706250
H 8.456145 16.467544 0.628245
H 8.568669 13.025319 9.358952
H 4.099183 9.739847 -0.380604
H 10.871306 16.189925 1.260277

40
2-*diss*-[Ni(I)]$^{+}$
Ni 6.659854 15.017182 5.085597
P 7.797792 13.817614 3.738577
N 6.273219 13.511397 6.193465
C 8.132549 12.449959 4.986141
C 6.899034 13.005637 2.350437
C 9.467356 14.259555 3.093192
C 6.987495 12.363232 5.971166
C 5.262005 13.526117 7.115778
H 8.340060 11.466269 4.518516
H 9.059963 12.755733 5.519324
C 5.734929 13.643803 1.866136
C 7.298176 11.771992 1.784927
C 10.349333 14.914453 3.986921
C 9.870630 14.054834 1.755414
C 6.680741 11.176897 6.655905
C 4.934661 12.365480 7.836138
C 4.532524 14.830159 7.295466
H 5.416318 14.597311 2.319324
C 4.986481 13.065504 0.825872
C 6.544743 11.193617 0.748742
H 8.200913 11.254823 2.148002
H 10.029145 15.118649 5.023048
C 11.623196 15.321032 3.559456
C 11.143699 14.475458 1.327842
H 9.192168 13.564788 1.040469
H 7.262934 10.266569 6.449749
C 5.644301 11.176627 7.602294
H 4.117391 12.401115 8.571637

H 4.041104 15.139445 6.347479
H 5.237734 15.639124 7.584423
H 3.753518 14.755348 8.077138
H 4.081184 13.571821 0.455447
C 5.390133 11.839395 0.267497
H 6.861409 10.231690 0.315466
H 12.302497 15.822066 4.267152
C 12.023902 15.102257 2.227117
H 11.447253 14.309003 0.282039
H 5.392184 10.257914 8.153525
H 4.801610 11.381986 -0.543532
H 13.020042 15.428776 1.889136

40
2-*diss*-[Ni(0)]^{0}
Ni 6.795499 15.206945 4.782103
P 8.018401 13.941546 3.751907
N 5.999026 14.011555 5.955566
C 7.886592 12.642168 5.169378
C 7.533480 12.973543 2.246958
C 9.869511 14.063273 3.575367
C 6.553771 12.743518 5.882888
C 4.808139 14.179714 6.627309
H 8.108238 11.597499 4.860688
H 8.699064 12.956673 5.862867
C 6.574161 11.931906 2.313993
C 7.983414 13.385487 0.966264
C 10.696559 13.060535 3.016370
C 10.470664 15.245408 4.063996
C 5.915095 11.636096 6.455566
C 4.131583 13.090577 7.203175
C 4.282457 15.589977 6.673880
H 6.193386 11.594798 3.291653
C 6.092274 11.317814 1.142949
C 7.510446 12.764869 -0.200752
H 8.717290 14.204947 0.884523
H 10.249314 12.129264 2.631959
C 12.089460 13.244107 2.944216

C 11.863791 15.425318 4.005246
H 9.814432 16.029135 4.483678
H 6.387922 10.644444 6.381223
C 4.681925 11.799891 7.122200
H 3.171715 13.262644 7.715308
H 3.801552 15.871828 5.710776
H 5.128847 16.296287 6.821636
H 3.535580 15.731285 7.480538
H 5.348590 10.507714 1.220524
C 6.558906 11.726712 -0.119498
H 7.884928 13.094437 -1.183805
H 12.723020 12.457448 2.502617
C 12.676984 14.424116 3.440231
H 12.316197 16.352834 4.392233
H 4.166725 10.936996 7.571310
H 6.183542 11.243300 -1.035460
H 13.768392 14.564134 3.382936

38
3-ligand
P -0.072902 -0.089198 -0.112275
N -0.092481 -0.418894 3.344499
C 0.822749 -1.121094 1.189319
C 1.260500 0.209824 -1.370496
C -0.060522 -1.378362 2.389646
C -0.874126 -0.621202 4.421529
H 1.184332 -2.074171 0.752355
H 1.688292 -0.505276 1.510189
C 2.548420 -0.373266 -1.343311
C 0.960766 1.136370 -2.399847
C -0.822772 -2.566260 2.487860
C -1.661952 -1.770448 4.612061
H 2.819320 -1.095139 -0.557780
C 3.506224 -0.041104 -2.319967
C 1.911429 1.454670 -3.384510
H -0.030397 1.620920 -2.426175
H -0.767196 -3.305493 1.671340
C -1.632548 -2.762673 3.617365

H -2.279552 -1.880535 5.516772
H 4.506164 -0.502440 -2.279910
C 3.190733 0.868644 -3.344599
H 1.656272 2.173680 -4.179472
H -2.232859 -3.680914 3.720857
H 3.941837 1.125307 -4.108487
C -1.134912 -1.411790 -0.988041
H -1.703470 -0.856115 -1.761437
H -1.859115 -1.748570 -0.217028
C -0.418145 -2.592018 -1.585888
C -0.040989 -2.602179 -2.950847
N -0.138152 -3.634674 -0.765321
C 0.630135 -3.718240 -3.470447
H -0.284366 -1.742725 -3.594281
C 0.510002 -4.699864 -1.274669
C 0.914775 -4.797717 -2.616336
H 0.924079 -3.747246 -4.531762
H 1.435715 -5.697037 -2.978734
H 0.715501 -5.527706 -0.571556
H -0.873199 0.179896 5.184256

39
3-*diss*-$[Ni(II)]^{2+}$
Ni 7.178240 15.744236 5.526419
P 6.630652 17.414708 4.344555
N 6.795466 16.777194 6.986178
C 5.799116 18.507835 5.572005
C 5.540960 16.915767 2.993351
C 6.186078 18.005943 6.933757
C 7.160126 16.235697 8.181055
H 6.136973 19.551231 5.366193
H 4.698264 18.478504 5.433792
C 4.137332 16.842927 3.182863
C 6.110062 16.485213 1.766613
C 5.945604 18.719270 8.118342
C 6.929211 16.904301 9.384385
H 3.676466 17.143612 4.135664
C 3.316951 16.387569 2.141550

C 5.278780 16.026474 0.735085
H 7.198663 16.502827 1.609644
H 5.463848 19.705560 8.056143
C 6.315065 18.168211 9.353357
H 7.234828 16.430961 10.328131
H 2.226921 16.347223 2.287879
C 3.884114 15.980359 0.918710
H 5.724194 15.701571 -0.217371
H 6.126802 18.721451 10.285484
H 3.235023 15.619197 0.106082
C 8.117162 18.313168 3.694195
H 8.701487 17.604596 3.075598
H 8.720761 18.537360 4.598250
C 7.774021 19.587862 2.942226
C 8.045663 19.713335 1.563456
N 7.221958 20.585985 3.665309
C 7.744518 20.927812 0.925935
H 8.501685 18.883126 1.003437
C 6.932294 21.743884 3.045681
C 7.176249 21.967415 1.678743
H 7.954345 21.057713 -0.147086
H 6.922781 22.934887 1.219920
H 6.481993 22.535468 3.670178
H 7.643554 15.245955 8.157969

39
3-*diss*-[Ni(I)]$^{+}$
Ni 6.934068 15.580371 5.635578
P 6.260826 17.138500 4.357656
N 7.056135 16.777301 7.104673
C 5.898790 18.421777 5.671330
C 4.729799 17.010621 3.346576
C 6.612324 18.067055 6.959046
C 7.657316 16.406936 8.269358
H 6.181888 19.436985 5.317720
H 4.799882 18.417969 5.843499
C 4.362540 15.720942 2.899620
C 3.913183 18.116725 3.013820

C 6.795733 19.006854 7.988265
C 7.854448 17.294960 9.329847
H 4.988122 14.856714 3.179838
C 3.206980 15.538435 2.120409
C 2.751610 17.929094 2.243821
H 4.175759 19.129950 3.356343
H 6.438827 20.037497 7.842469
C 7.419201 18.624668 9.186883
H 8.346553 16.945202 10.249271
H 2.931378 14.528986 1.776442
C 2.399532 16.643137 1.793236
H 2.118199 18.794770 1.993220
H 7.563342 19.354858 9.997659
H 1.489106 16.501556 1.189520
C 7.527389 17.980899 3.237134
H 7.832795 17.210722 2.500998
H 8.391574 18.174887 3.906252
C 7.093099 19.252395 2.553696
C 6.755857 19.264790 1.180424
N 7.038348 20.373357 3.313653
C 6.366277 20.472930 0.582655
H 6.811296 18.337112 0.590233
C 6.657415 21.525280 2.730842
C 6.315233 21.634246 1.371463
H 6.107913 20.506961 -0.487560
H 6.018741 22.605399 0.946866
H 7.989306 15.358101 8.331178
H 6.626482 22.416566 3.383776

39

3-*diss*-[Ni(0)]^0

Ni 6.710241 15.582701 5.260677
P 5.606199 16.852055 4.118107
N 7.537390 16.797540 6.369604
C 5.746323 18.215028 5.454840
C 3.784402 16.867491 3.723953
C 7.087131 18.095801 6.148265
C 8.748335 16.641436 6.998557

H 5.568824 19.253089 5.100645
H 4.932544 17.964417 6.172888
C 2.867336 17.868302 4.123211
C 3.285548 15.736129 3.034763
C 7.851095 19.204922 6.529686
C 9.562470 17.718152 7.372685
H 3.218149 18.767238 4.653508
C 1.493897 17.736585 3.842157
C 1.919487 15.613811 2.731994
H 3.989533 14.933138 2.751743
H 7.455504 20.215504 6.341922
C 9.113998 19.030466 7.143506
H 10.533985 17.520622 7.852761
H 0.792962 18.522825 4.167331
C 1.015932 16.614182 3.141947
H 1.554357 14.728170 2.186897
H 9.721581 19.900386 7.435732
H -0.059014 16.514852 2.921528
C 6.342650 17.707728 2.555305
H 6.202821 16.964419 1.744781
H 7.426496 17.782242 2.780690
C 5.765794 19.027987 2.144762
C 6.284366 20.250381 2.647160
N 4.716523 19.005461 1.282207
C 5.699642 21.461436 2.253180
H 7.152091 20.241899 3.325732
C 4.163148 20.175616 0.916189
C 4.606267 21.431718 1.367523
H 6.096699 22.419406 2.625931
H 4.114572 22.355919 1.027157
H 9.049101 15.601081 7.200953
H 3.313646 20.113623 0.209761

77

3-singlet-$[Ni]^{2+}$@MeCN(M06-L)
P 10.431367 7.459071 5.837319
N 10.505479 7.577607 3.075554
N 12.850567 9.025992 4.562915

C 9.832727 6.049747 4.821191
C 10.359175 6.278392 3.442763
C 10.746839 5.243291 2.595470
C 11.312612 5.545756 1.360657
C 11.496017 6.880577 1.009459
C 11.079755 7.864859 1.894197
C 12.237931 7.239050 6.062865
C 13.016573 7.730877 4.875199
C 13.849747 6.886786 4.138412
C 14.545021 7.413505 3.050583
C 14.386598 8.760649 2.736980
C 13.524414 9.522956 3.524373
C 9.646205 7.372276 7.456864
C 10.374292 7.206043 8.643048
C 9.711816 7.214107 9.869528
C 8.329401 7.386619 9.921592
C 7.600460 7.548230 8.742076
C 8.252657 7.544678 7.512056
P 9.580213 10.542665 5.836784
N 9.506755 10.423318 3.075007
N 7.161557 8.975078 4.562567
C 10.179147 11.951658 4.820366
C 9.653015 11.722632 3.441880
C 9.265578 12.757501 2.594202
C 8.700091 12.454701 1.359340
C 8.516740 11.119785 1.008472
C 8.932767 10.135743 1.893590
C 7.773595 10.762798 6.061791
C 6.995283 10.270317 4.874184
C 6.162177 11.113935 4.136781
C 5.467258 10.586585 3.049030
C 5.625960 9.239312 2.736115
C 6.488057 8.477509 3.524092
C 10.364959 10.629967 7.456504
C 9.636535 10.796388 8.642455
C 10.298684 10.788737 9.869113
C 11.681110 10.616436 9.921589
C 12.410385 10.454619 8.742308

C 11.758516 10.457766 7.512111
Ni 10.005929 9.000641 4.376301
H 12.326370 10.304202 6.587623
H 13.493260 10.318929 8.779649
H 12.193208 10.606618 10.885977
H 9.725012 10.914535 10.789579
H 8.553448 10.934436 8.625294
H 7.499369 10.160201 6.942600
H 7.555505 11.813252 6.302856
H 6.061651 12.165880 4.412368
H 4.811975 11.224749 2.452570
H 5.102232 8.785675 1.893547
H 6.650726 7.416709 3.301121
H 11.281487 11.908440 4.839796
H 9.887066 12.929997 5.226710
H 9.396910 13.790896 2.915897
H 8.387716 13.254787 0.686336
H 8.048892 10.834318 0.066452
H 8.788649 9.077167 1.665590
H 11.223909 8.923375 1.665935
H 11.964088 7.165790 0.067472
H 11.625164 4.745488 0.687951
H 10.615454 4.209984 2.917426
H 10.124765 5.071546 5.227899
H 8.730379 6.092912 4.840346
H 13.361972 10.583672 3.300843
H 14.910606 9.213805 1.894328
H 15.200364 6.774957 2.454600
H 13.950062 5.834963 4.414542
H 12.455940 6.188713 6.304508
H 12.511937 7.842082 6.943445
H 11.457365 7.067841 8.626208
H 10.285223 7.088460 10.790179
H 7.817049 7.396758 10.885842
H 6.517593 7.684076 8.779096
H 7.685074 7.698059 6.587369

77

3-triplet-[Ni]$^{2+}$@MeCN(M06-L)
P 10.344436 7.182285 5.634706
N 10.279747 7.181801 2.734452
N 12.021277 8.989889 3.976489
C 9.916476 5.698359 4.639367
C 10.398439 5.931841 3.238780
C 11.006522 4.908745 2.507769
C 11.505850 5.173983 1.237585
C 11.400547 6.467016 0.732072
C 10.784713 7.432153 1.517101
C 12.174127 7.247697 5.703738
C 12.771220 8.038054 4.570663
C 14.097067 7.824402 4.185279
C 14.664125 8.624561 3.199484
C 13.894034 9.630826 2.621540
C 12.576496 9.769082 3.036060
C 9.715281 6.955032 7.310750
C 10.538553 6.918336 8.446055
C 9.973947 6.859197 9.719650
C 8.588938 6.833242 9.876046
C 7.762126 6.861969 8.751322
C 8.318553 6.928016 7.477455
P 9.541239 10.601822 5.644515
N 9.352278 10.540433 2.744769
N 7.728382 8.757136 4.181067
C 9.836013 12.073398 4.585459
C 9.264529 11.801835 3.225643
C 8.616281 12.808654 2.506322
C 8.045875 12.515065 1.273014
C 8.120278 11.210154 0.792947
C 8.777010 10.261891 1.565113
C 7.731788 10.499825 5.913827
C 7.029276 9.694967 4.854567
C 5.664410 9.879358 4.619673
C 5.008991 9.067351 3.700878
C 5.732149 8.079216 3.037015
C 7.089700 7.967714 3.304241
C 10.334310 10.884082 7.240883

C 9.630848 10.935629 8.453451
C 10.321074 11.041997 9.660350
C 11.713731 11.101817 9.672574
C 12.421448 11.059407 8.469787
C 11.739556 10.945487 7.262051
Ni 9.876801 8.880411 4.119685
H 12.307914 10.893026 6.327979
H 13.512218 11.106976 8.471504
H 12.249901 11.182601 10.620323
H 9.761228 11.080226 10.597169
H 8.539557 10.900242 8.471260
H 7.592889 9.972953 6.873731
H 7.261494 11.485682 6.043701
H 5.126296 10.655409 5.165878
H 3.943465 9.203517 3.507763
H 5.261878 7.413356 2.313565
H 7.703158 7.226192 2.783809
H 10.929637 12.212065 4.532344
H 9.424354 12.998101 5.012959
H 8.557499 13.813415 2.926728
H 7.534556 13.292172 0.702289
H 7.669456 10.921554 -0.156502
H 8.817829 9.220735 1.234095
H 10.720908 8.464608 1.163128
H 11.796414 6.733823 -0.247807
H 11.987046 4.384519 0.657686
H 11.092170 3.914462 2.948038
H 10.330009 4.768965 5.054473
H 8.817991 5.597833 4.668741
H 11.925288 10.523541 2.584768
H 14.295779 10.289546 1.851739
H 15.698450 8.465753 2.889357
H 14.675858 7.036136 4.668969
H 12.633872 6.251429 5.781165
H 12.430917 7.770717 6.640908
H 11.626381 6.927746 8.351443
H 10.625984 6.831289 10.595142
H 8.151455 6.789689 10.875508

H 6.676531 6.840499 8.865780
H 7.658451 6.967884 6.605076

89
4-singlet-$[Ni]^{2+}$@MeCN(M06-L)
P 10.614771 7.502470 5.761050
N 10.864513 7.733053 3.026857
N 13.430439 8.981567 4.742119
C 10.122056 6.111429 4.657489
C 10.720072 6.413963 3.320181
C 11.161891 5.422629 2.453264
C 11.798722 5.798387 1.273938
C 12.009104 7.146708 1.021034
C 11.535749 8.112076 1.915104
C 12.399321 7.287802 6.112636
C 13.302902 7.669344 4.975112
C 13.963605 6.700715 4.217693
C 14.786953 7.127608 3.177498
C 14.938759 8.490537 2.951213
C 14.247848 9.399695 3.765711
C 9.738742 7.350751 7.329205
C 10.401642 7.183923 8.552851
C 9.669961 7.158365 9.739267
C 8.283073 7.297147 9.713443
C 7.619882 7.460661 8.495665
C 8.341237 7.492214 7.305285
P 9.396557 10.498043 5.761753
N 9.147974 10.269260 3.027229
N 6.581665 9.019543 4.740546
C 9.889839 11.889735 4.659282
C 9.292300 11.588142 3.321556
C 8.850821 12.580115 2.455197
C 8.214471 12.205232 1.275335
C 8.004253 10.857096 1.021329
C 8.477255 9.891065 1.914876
C 7.611871 10.712555 6.112686
C 6.708823 10.331631 4.974529
C 6.048210 11.300655 4.217542

C 5.225353 10.874326 3.176724
C 5.073914 9.511528 2.949421
C 5.764703 8.601937 3.763541
C 10.271967 10.648802 7.330341
C 9.608624 10.814923 8.553839
C 10.339882 10.839820 9.740530
C 11.726782 10.701078 9.715124
C 12.390412 10.538258 8.497493
C 11.669481 10.507371 7.306841
Ni 10.005934 9.000701 4.319079
H 12.195671 10.335943 6.358694
H 13.476500 10.423849 8.473422
H 12.293612 10.715980 10.648326
H 9.817236 10.964123 10.691050
H 8.523097 10.923528 8.598182
H 7.392943 10.064957 6.975060
H 7.457129 11.754326 6.431483
H 6.180681 12.362570 4.436255
H 4.703970 11.602641 2.551484
H 4.429392 9.145402 2.147393
H 10.992189 11.898446 4.620993
H 9.578598 12.872150 5.043013
H 8.988495 13.628464 2.720319
H 7.856119 12.962366 0.575967
H 7.462287 10.531255 0.132429
H 12.551475 7.473200 0.132621
H 12.157343 5.041770 0.574147
H 11.024101 4.374086 2.717561
H 10.433266 5.128797 5.040692
H 9.019724 6.102623 4.618790
H 15.583658 8.857096 2.149686
H 15.308423 6.399625 2.551942
H 13.830806 5.638671 4.435586
H 12.553922 6.245851 6.430914
H 12.617866 7.934918 6.975465
H 11.487149 7.075277 8.597528
H 10.192267 7.033521 10.689903
H 7.715909 7.281734 10.646433

H 6.533805 7.575097 8.471273
H 7.815382 7.664173 6.357048
C 8.244342 8.443369 1.652565
H 9.001666 8.026780 0.970117
H 8.260337 7.850616 2.575239
H 7.273934 8.297906 1.161497
C 11.768840 9.559974 1.654079
H 11.011960 9.977112 0.971476
H 11.752240 10.151971 2.577237
H 12.739571 9.705815 1.163756
C 14.416282 10.874091 3.599534
H 13.493013 11.412396 3.847022
H 15.190712 11.248962 4.286077
H 14.729228 11.143066 2.583330
C 5.596581 7.127626 3.596287
H 6.519721 6.589270 3.844133
H 4.821665 6.752229 4.281994
H 5.284429 6.859256 2.579677

89
4-triplet-$[Ni]^{2+}$@MeCN(M06-L)
P 10.115484 7.110658 5.348687
N 10.885854 5.970170 2.452117
N 11.909144 8.707960 3.667407
C 10.148264 5.395959 4.676130
C 11.165034 5.297841 3.579148
C 12.354921 4.591120 3.760388
C 13.286443 4.588928 2.722639
C 12.991802 5.274482 1.552793
C 11.766108 5.951873 1.443589
C 11.855419 7.463910 5.788129
C 12.604244 8.042055 4.618026
C 13.990947 7.920434 4.544362
C 14.667257 8.508493 3.482353
C 13.942826 9.196408 2.515506
C 12.554506 9.281845 2.624876
C 9.130349 7.047753 6.858792
C 9.662225 7.256303 8.139180

C 8.817841 7.283270 9.248927
C 7.445113 7.095578 9.092866
C 6.910713 6.869513 7.822379
C 7.745690 6.848159 6.709434
P 9.747205 10.634819 5.358367
N 8.710546 11.734281 2.512955
N 7.796153 9.031927 3.890700
C 9.669947 12.332233 4.647018
C 8.552952 12.425911 3.652216
C 7.396539 13.152489 3.939875
C 6.366893 13.156580 2.999627
C 6.534856 12.451094 1.816754
C 7.733491 11.752012 1.597350
C 8.054357 10.312551 5.972579
C 7.194952 9.714709 4.890922
C 5.806934 9.830237 4.946373
C 5.036709 9.218377 3.964308
C 5.667980 8.511342 2.946281
C 7.060377 8.432708 2.925880
C 10.888465 10.727277 6.751272
C 10.505613 10.534405 8.085903
C 11.470452 10.528719 9.093221
C 12.815385 10.722000 8.780331
C 13.201506 10.931716 7.454525
C 12.246063 10.931689 6.443151
Ni 9.849379 8.867265 3.853345
H 12.560605 11.077717 5.404233
H 14.253126 11.086729 7.205068
H 13.566985 10.711871 9.572170
H 11.164259 10.371928 10.129755
H 9.456266 10.388746 8.352476
H 8.133516 9.589524 6.803291
H 7.582374 11.210014 6.400416
H 5.346765 10.395311 5.757953
H 3.948343 9.294555 3.991362
H 5.092949 8.021874 2.159485
H 10.642883 12.536538 4.174487
H 9.541621 13.041940 5.476636

H 7.306267 13.701435 4.879460
H 5.444099 13.707908 3.192385
H 5.751029 12.435238 1.056361
H 13.696828 5.291032 0.718824
H 14.232531 4.054166 2.830243
H 12.547032 4.057385 4.693534
H 10.368820 4.708796 5.505313
H 9.137966 5.164986 4.305092
H 14.440785 9.665238 1.666108
H 15.752941 8.427091 3.406674
H 14.523924 7.367958 5.319282
H 12.375374 6.581258 6.190417
H 11.845778 8.205713 6.605742
H 10.735819 7.397108 8.284069
H 9.240582 7.452496 10.241603
H 6.788123 7.122653 9.964395
H 5.836849 6.718201 7.695905
H 7.314485 6.687888 5.715555
C 11.728479 9.988389 1.610427
H 10.996281 9.301746 1.158414
H 11.150986 10.817244 2.050007
H 12.344018 10.394841 0.800935
C 11.405312 6.665485 0.181998
H 12.168773 7.406464 -0.096810
H 11.342174 5.961794 -0.660626
H 10.435923 7.167887 0.274523
C 7.951122 11.008526 0.320265
H 7.888880 11.685620 -0.543617
H 8.935281 10.527118 0.304577
H 7.176496 10.243404 0.162630
C 7.804123 7.704418 1.863349
H 8.469028 8.389312 1.313068
H 8.447624 6.914065 2.282832
H 7.127800 7.244052 1.135331

2

$H_{2(g)}$(M06-L)

H 0.000000 0.000000 0.022699

H 0.000000 0.000000 0.777301

77

3-singlet-$[Ni]^{2+}$(M06-L)

P 10.431919 7.458559 5.837305
N 10.506744 7.578147 3.075932
N 12.852731 9.025877 4.565371
C 9.833944 6.049352 4.820729
C 10.360680 6.278766 3.442527
C 10.748808 5.244195 2.594826
C 11.314694 5.547469 1.360275
C 11.497728 6.882492 1.009696
C 11.081090 7.866257 1.894829
C 12.238383 7.238209 6.063476
C 13.017178 7.730214 4.876027
C 13.848430 6.885738 4.137484
C 14.543304 7.412723 3.049531
C 14.386410 8.760436 2.737565
C 13.526115 9.523105 3.526692
C 9.646423 7.371893 7.456822
C 10.374290 7.205048 8.643047
C 9.711777 7.213871 9.869516
C 8.329517 7.387657 9.921521
C 7.600797 7.549773 8.741944
C 8.253047 7.545530 7.511941
P 9.579680 10.542928 5.836719
N 9.505578 10.422398 3.075284
N 7.159064 8.975466 4.564769
C 10.178156 11.951669 4.819761
C 9.651635 11.721882 3.441543
C 9.263658 12.756237 2.593512
C 8.697937 12.452652 1.358960
C 8.514950 11.117542 1.008700
C 8.931445 10.134000 1.894152
C 7.773178 10.763706 6.062272
C 6.994660 10.271286 4.874798
C 6.163621 11.115470 4.135691
C 5.468984 10.588030 3.047805

C 5.625864 9.240172 2.736472
C 6.485912 8.477799 3.526155
C 10.364754 10.630187 7.456405
C 9.636508 10.796677 8.642447
C 10.298722 10.788332 9.869079
C 11.681076 10.615407 9.921439
C 12.410181 10.453686 8.742044
C 11.758227 10.457439 7.511882
Ni 10.005949 9.000489 4.376629
H 12.325934 10.303733 6.587324
H 13.492984 10.317340 8.779209
H 12.193222 10.604908 10.885806
H 9.725094 10.913968 10.789603
H 8.553490 10.935211 8.625367
H 7.498741 10.161416 6.943192
H 7.555755 11.814352 6.303031
H 6.064731 12.167900 4.409996
H 4.815520 11.226665 2.449850
H 5.102562 8.786523 1.893644
H 6.647233 7.416543 3.304417
H 11.280507 11.908885 4.838674
H 9.885732 12.930006 5.225812
H 9.394686 13.789784 2.914787
H 8.385050 13.252330 0.685716
H 8.046921 10.831467 0.066964
H 8.787615 9.075275 1.666707
H 11.224967 8.924927 1.667159
H 11.965891 7.168325 0.067952
H 11.627657 4.747620 0.687270
H 10.617763 4.210728 2.916356
H 10.126526 5.071228 5.227186
H 8.731578 6.091862 4.839436
H 13.364778 10.584242 3.304407
H 14.909882 9.213755 1.894665
H 15.196957 6.773859 2.452026
H 13.947285 5.833430 4.412266
H 12.455967 6.187716 6.304766
H 12.512382 7.840943 6.944224

H 11.457229 7.065857 8.626227
H 10.285095 7.087941 10.790193
H 7.817144 7.398546 10.885764
H 6.518072 7.686812 8.778846
H 7.685686 7.699573 6.587223

77
3-triplet-$[Ni]^{2+}$(M06-L)
P 10.345076 7.181402 5.634583
N 10.279744 7.182221 2.735024
N 12.021814 8.989558 3.976799
C 9.914990 5.698236 4.639136
C 10.396953 5.931836 3.238597
C 11.003772 4.908478 2.506909
C 11.503292 5.173962 1.236864
C 11.399537 6.467427 0.732166
C 10.784948 7.432832 1.517847
C 12.174822 7.244994 5.701708
C 12.771537 8.036500 4.569260
C 14.097001 7.822514 4.182752
C 14.663948 8.623680 3.197724
C 13.894169 9.631257 2.621702
C 12.576941 9.769732 3.037159
C 9.717230 6.953996 7.311203
C 10.541390 6.917005 8.445863
C 9.977786 6.858247 9.719938
C 8.592874 6.832962 9.877497
C 7.765200 6.862002 8.753416
C 8.320644 6.927671 7.479092
P 9.540919 10.601982 5.644941
N 9.352786 10.539926 2.745379
N 7.728624 8.757080 4.181012
C 9.836485 12.073223 4.585700
C 9.265191 11.801448 3.225894
C 8.617177 12.808169 2.506223
C 8.046889 12.514288 1.272945
C 8.121154 11.209246 0.793248
C 8.777619 10.261083 1.565769

C 7.731294 10.500429 5.913061
C 7.029327 9.695361 4.853659
C 5.664703 9.880167 4.617687
C 5.009695 9.068059 3.698706
C 5.732995 8.079432 3.035768
C 7.090313 7.967566 3.304010
C 10.332579 10.885354 7.241927
C 9.628098 10.936848 8.453918
C 10.317283 11.043816 9.661377
C 11.709916 11.104345 9.674748
C 12.418604 11.062027 8.472528
C 11.737746 10.947495 7.264267
Ni 9.877054 8.880198 4.120316
H 12.306880 10.894641 6.330711
H 13.509362 11.109905 8.475037
H 12.245282 11.185455 10.622941
H 9.756602 11.081899 10.597718
H 8.536823 10.900864 8.470796
H 7.591529 9.973897 6.872997
H 7.261168 11.486437 6.042257
H 5.126569 10.656673 5.163201
H 3.944378 9.204579 3.504692
H 5.263148 7.413488 2.312129
H 7.703802 7.225735 2.784122
H 10.930127 12.211636 4.532716
H 9.424805 12.998024 5.012919
H 8.558448 13.813001 2.926431
H 7.535717 13.291305 0.701965
H 7.670380 10.920350 -0.156127
H 8.818262 9.219846 1.235044
H 10.722475 8.465595 1.164577
H 11.795732 6.734468 -0.247507
H 11.983548 4.384291 0.656466
H 11.088387 3.913883 2.946641
H 10.327877 4.768368 5.053783
H 8.816445 5.598719 4.668719
H 11.925988 10.525164 2.587180
H 14.295756 10.290811 1.852543

H 15.697959 8.464591 2.886693
H 14.675485 7.033156 4.665004
H 12.633654 6.248183 5.777206
H 12.433220 7.766492 6.639243
H 11.629128 6.925925 8.350321
H 10.630564 6.830126 10.594887
H 8.156169 6.789855 10.877332
H 6.679670 6.841308 8.868645
H 7.659816 6.968072 6.607299

77
3-doublet-$[Ni(I)]^{+}$(M06-L)
P 10.588294 7.380560 5.864835
N 10.562035 7.556469 3.028141
N 13.098398 8.874458 4.728053
C 9.981289 5.996373 4.788773
C 10.499964 6.256444 3.408048
C 10.958235 5.234760 2.576382
C 11.515157 5.553203 1.340716
C 11.618226 6.893499 0.976060
C 11.128749 7.857053 1.848956
C 12.384887 7.012310 6.092762
C 13.160484 7.546816 4.922950
C 13.869226 6.703996 4.061124
C 14.536299 7.258171 2.970664
C 14.478217 8.636222 2.775547
C 13.744938 9.396490 3.685258
C 9.791384 7.160910 7.471774
C 10.451538 6.738506 8.635231
C 9.777094 6.715019 9.855910
C 8.440191 7.109377 9.930444
C 7.776696 7.533150 8.776998
C 8.446400 7.567798 7.556130
P 9.423505 10.620986 5.864653
N 9.450004 10.444940 3.027977
N 6.913812 9.126913 4.727655
C 10.030687 12.005083 4.788577
C 9.512001 11.744975 3.407864

C 9.053614 12.766614 2.576210
C 8.496648 12.448111 1.340577
C 8.393660 11.107805 0.975942
C 8.883259 10.144297 1.848826
C 7.626887 10.989265 6.092310
C 6.851426 10.454551 4.922500
C 6.142551 11.297181 4.060601
C 5.475611 10.742810 2.970159
C 5.533982 9.364761 2.775112
C 6.267416 8.604693 3.684865
C 10.220238 10.840648 7.471676
C 9.559981 11.263136 8.635044
C 10.234279 11.286586 9.855804
C 11.571128 10.892077 9.930516
C 12.234724 10.468221 8.777159
C 11.565173 10.433633 7.556206
Ni 10.005900 9.000704 4.412344
H 12.077833 10.069708 6.655627
H 13.279086 10.150257 8.830920
H 12.094391 10.910891 10.889187
H 9.710372 11.618518 10.755176
H 8.514276 11.579323 8.596578
H 7.322416 10.451789 7.005236
H 7.440053 12.061211 6.255522
H 6.122051 12.374274 4.243167
H 4.922040 11.382225 2.278604
H 5.029694 8.887151 1.933397
H 6.342438 7.517347 3.562255
H 11.132859 11.946728 4.811979
H 9.751733 13.008216 5.143629
H 9.125008 13.803038 2.910449
H 8.129773 13.236226 0.680204
H 7.934526 10.805461 0.033983
H 8.809156 9.079617 1.608354
H 11.202930 8.921725 1.608475
H 12.077332 7.195799 0.034073
H 11.881932 4.765052 0.680330
H 10.886788 4.198347 2.910645

H 10.260325 4.993279 5.143878
H 8.879108 6.054609 4.812142
H 13.670151 10.483844 3.562588
H 14.982612 9.113683 1.933811
H 15.089751 6.618605 2.279154
H 13.889517 5.626911 4.243759
H 12.571666 5.940381 6.256155
H 12.689263 7.549913 7.005643
H 11.497279 6.422424 8.596897
H 10.300921 6.383157 10.755353
H 7.916808 7.090519 10.889049
H 6.732292 7.851000 8.830623
H 7.933819 7.931628 6.655468

77
3-quartet-$[Ni(I)]^{+}$(M06-L)
P 10.024694 7.200090 5.695074
N 10.215678 7.353985 2.794711
N 12.163226 8.669412 4.297913
C 9.421988 5.881094 4.566416
C 10.131550 6.074504 3.261276
C 10.725096 5.006440 2.594613
C 11.430703 5.227943 1.409101
C 11.541826 6.543176 0.947549
C 10.932626 7.558948 1.665490
C 11.816336 6.895053 5.956130
C 12.646721 7.542435 4.881404
C 13.893683 7.026750 4.536181
C 14.685714 7.689767 3.595322
C 14.190597 8.860763 3.017137
C 12.931972 9.306952 3.391381
C 9.166756 7.075310 7.273117
C 9.739500 7.608569 8.442452
C 9.006557 7.668370 9.624929
C 7.690154 7.206270 9.663385
C 7.111262 6.678939 8.507933
C 7.837984 6.614805 7.321682
P 9.984782 10.690121 5.836153

N 9.845417 10.696750 2.895505
N 7.904524 9.260209 4.296444
C 10.406730 12.142396 4.782200
C 9.786569 11.950431 3.433725
C 9.145443 13.001055 2.783400
C 8.546352 12.794391 1.537858
C 8.585939 11.504505 0.998777
C 9.226765 10.501689 1.706876
C 8.190471 10.768147 6.200564
C 7.379440 10.272092 5.038215
C 6.119941 10.797170 4.763978
C 5.357184 10.269540 3.717951
C 5.895458 9.218599 2.970604
C 7.161780 8.752593 3.289469
C 10.859018 10.891989 7.402176
C 10.203847 11.117319 8.622986
C 10.930612 11.171731 9.811419
C 12.315817 11.009364 9.798432
C 12.977695 10.793766 8.587210
C 12.256674 10.727811 7.398616
Ni 10.043248 8.995665 4.247907
H 12.781302 10.532987 6.457796
H 14.062039 10.665029 8.569539
H 12.881209 11.050018 10.731838
H 10.407244 11.344027 10.754637
H 9.119999 11.250284 8.656845
H 8.046854 10.077528 7.053434
H 7.851175 11.759227 6.536064
H 5.736979 11.613101 5.380211
H 4.366701 10.669703 3.495122
H 5.344210 8.765781 2.145089
H 7.622635 7.940356 2.718390
H 11.509211 12.139456 4.717510
H 10.114230 13.101275 5.232795
H 9.122601 13.983315 3.259748
H 8.047691 13.611800 1.014614
H 8.114576 11.270990 0.042771
H 9.234823 9.480655 1.317823

H 11.036966 8.597015 1.339060
H 12.103035 6.784549 0.043466
H 11.898153 4.399666 0.874332
H 10.631119 4.001949 3.012013
H 9.560983 4.865164 4.962145
H 8.337602 6.044841 4.448852
H 12.499301 10.210609 2.949965
H 14.767468 9.420434 2.279546
H 15.667530 7.299212 3.322475
H 14.245137 6.112526 5.018824
H 12.054237 5.826119 6.060227
H 12.070189 7.357437 6.925512
H 10.762092 7.996859 8.437199
H 9.470086 8.086260 10.521656
H 7.116539 7.257680 10.591071
H 6.082423 6.312994 8.527635
H 7.359713 6.206679 6.427775

77
3-singlet-$[Ni(0)]^0$(M06-L)
P 10.664894 7.247646 5.742314
N 9.569281 5.496869 3.142409
N 11.433229 8.606680 3.348352
C 10.357943 5.400811 5.427818
C 10.527791 5.073349 3.986281
C 11.674813 4.406382 3.527536
C 11.832629 4.175768 2.165495
C 10.840792 4.621740 1.291918
C 9.733406 5.268731 1.837087
C 12.434177 7.364561 5.173401
C 12.462362 7.804184 3.742577
C 13.482566 7.440541 2.866902
C 13.475567 7.909235 1.552368
C 12.432283 8.747897 1.157395
C 11.446746 9.069548 2.080155
C 10.790092 7.179569 7.567192
C 11.988114 7.026907 8.278810
C 11.986182 7.008417 9.675073

C 10.787688 7.124467 10.378541
C 9.587153 7.268400 9.680181
C 9.592348 7.305770 8.288838
P 9.250685 10.549355 5.767538
N 10.090354 12.229019 3.036779
N 8.260607 9.127496 3.494128
C 9.515424 12.388534 5.380628
C 9.211974 12.675586 3.952777
C 8.024562 13.326954 3.583225
C 7.740059 13.516503 2.235483
C 8.649116 13.046903 1.287304
C 9.805391 12.418881 1.746156
C 7.435037 10.413587 5.375023
C 7.271355 9.938944 3.964477
C 6.171119 10.279832 3.181195
C 6.055414 9.779883 1.883342
C 7.059297 8.932698 1.411746
C 8.128053 8.633067 2.244827
C 9.302764 10.662664 7.593711
C 8.181453 10.849927 8.413856
C 8.319988 10.906589 9.802256
C 9.580360 10.795025 10.388634
C 10.705394 10.616382 9.581160
C 10.563849 10.540914 8.198546
Ni 9.904452 8.883846 4.626260
H 11.441694 10.360677 7.567119
H 11.695273 10.515477 10.033441
H 9.685734 10.840164 11.475399
H 7.435203 11.045116 10.428890
H 7.185760 10.956713 7.974398
H 7.056607 9.628724 6.055649
H 6.836827 11.319696 5.569108
H 5.408441 10.943549 3.595307
H 5.200794 10.044549 1.257677
H 7.019340 8.505464 0.407887
H 8.930896 7.967380 1.915318
H 10.571774 12.597645 5.607860
H 8.888925 12.992202 6.054958

H 7.335139 13.679201 4.355136
H 6.821219 14.021533 1.927926
H 8.471631 13.169893 0.217300
H 10.548566 12.053035 1.025772
H 8.927584 5.617415 1.178155
H 10.918148 4.466678 0.214200
H 12.718000 3.657375 1.789532
H 12.432340 4.073777 4.242204
H 11.040863 4.810459 6.057685
H 9.325933 5.204602 5.756028
H 10.616483 9.729077 1.811724
H 12.377282 9.150902 0.144274
H 14.267106 7.627068 0.855414
H 14.280530 6.784634 3.222851
H 13.044884 6.459710 5.331144
H 12.879718 8.163155 5.794813
H 12.937513 6.917860 7.747219
H 12.929475 6.896572 10.215788
H 10.789190 7.109675 11.471227
H 8.644831 7.372100 10.224149
H 8.654812 7.458651 7.741668

77
3-triplet-[Ni(0)]^0(M06-L)
P 10.341551 7.232639 5.936916
N 10.117657 7.033924 2.994208
N 12.017626 8.752658 4.051092
C 10.189877 5.639907 4.987101
C 10.573722 5.868338 3.559038
C 11.338287 4.956209 2.845254
C 11.654429 5.193885 1.495558
C 11.173358 6.383020 0.915931
C 10.432746 7.257272 1.688157
C 12.170136 7.445788 6.090865
C 12.750869 7.850657 4.771149
C 13.974366 7.369456 4.322644
C 14.502427 7.819212 3.101615
C 13.758236 8.764765 2.377761

C 12.540627 9.194281 2.878313
C 9.786493 6.812213 7.607768
C 10.552562 7.063897 8.763038
C 10.023590 6.842395 10.033566
C 8.724010 6.355598 10.187755
C 7.951939 6.098977 9.050003
C 8.469317 6.334236 7.780766
P 9.612975 10.635536 5.394652
N 9.740458 11.450063 2.279151
N 7.878172 9.009750 3.783596
C 9.716412 12.292528 4.544459
C 9.087148 12.190843 3.192488
C 7.843882 12.775952 2.923273
C 7.261699 12.591775 1.671749
C 7.941187 11.825899 0.724768
C 9.175206 11.285466 1.080859
C 7.813301 10.451501 5.736409
C 7.134575 9.859980 4.538156
C 5.803288 10.140996 4.234242
C 5.201772 9.535144 3.133764
C 5.961620 8.645316 2.371931
C 7.281565 8.414041 2.730995
C 10.375320 10.939028 7.017360
C 9.662669 11.025972 8.222964
C 10.338709 11.164025 9.436149
C 11.731712 11.225620 9.463823
C 12.451481 11.147561 8.269327
C 11.779921 10.998558 7.058432
Ni 9.879663 8.697273 4.263509
H 12.350659 10.907843 6.127764
H 13.543014 11.192626 8.282425
H 12.257433 11.333879 10.415138
H 9.769175 11.227173 10.366513
H 8.570460 10.990293 8.226230
H 7.750691 9.725765 6.568864
H 7.312014 11.371575 6.075147
H 5.248930 10.839326 4.865024
H 4.162384 9.750040 2.878204

H 5.540919 8.138243 1.502075
H 7.907611 7.722161 2.162226
H 10.790683 12.516987 4.453474
H 9.251022 13.075689 5.161174
H 7.340090 13.364320 3.694817
H 6.291224 13.037049 1.440658
H 7.526969 11.652598 -0.270195
H 9.741365 10.686161 0.356588
H 10.050167 8.193051 1.263429
H 11.380171 6.629137 -0.128343
H 12.246498 4.479002 0.921014
H 11.679039 4.045751 3.346218
H 10.772439 4.823800 5.441013
H 9.124040 5.356490 5.060448
H 11.936049 9.939832 2.348185
H 14.122463 9.164396 1.428228
H 15.463565 7.452113 2.736223
H 14.520184 6.648395 4.937197
H 12.669689 6.556277 6.505338
H 12.311512 8.267415 6.817305
H 11.576091 7.437973 8.676891
H 10.639432 7.046293 10.913538
H 8.313666 6.180730 11.184650
H 6.931968 5.720334 9.154450
H 7.837094 6.155928 6.904440

78
3-[Ni-NH]$^{2+}$(M06-L)
Ni 0.548716 -0.016833 0.325372
P 0.336687 1.953032 1.320017
P -1.378960 -1.058576 0.861698
N -0.857078 2.012720 -1.613890
N 0.760508 -1.438764 -1.045181
C -0.885477 2.008144 -2.957222
C -1.197909 3.171278 -3.632936
C -1.463923 4.322998 -2.889601
C -1.420336 4.286490 -1.498182
C -1.113740 3.095760 -0.845374

C -1.090079 2.933185 0.633836
C -0.170946 1.737687 3.055430
C -0.999273 2.638422 3.738292
C -1.393787 2.373073 5.048012
C -0.963219 1.210778 5.689678
C -0.126094 0.315324 5.022756
C 0.264631 0.576121 3.710269
C 1.967702 -1.668859 -1.594461
C 2.207774 -2.734013 -2.449365
C 1.156440 -3.600850 -2.742726
C -0.093521 -3.362905 -2.179609
C -0.272514 -2.266237 -1.337151
C -1.605723 -1.926095 -0.747217
C -2.943198 -0.211390 1.222694
C -3.881603 0.126900 0.235529
C -5.011551 0.875840 0.562942
C -5.224093 1.294137 1.876147
C -4.301503 0.956643 2.867197
C -3.169374 0.213165 2.544258
H -0.651191 1.060801 -3.442401
H -1.226912 3.173126 -4.721271
H -1.706955 5.256876 -3.398240
H -1.635178 5.176047 -0.906081
H -1.998007 2.388521 0.951048
H -1.149757 3.925020 1.104215
H -1.345123 3.557727 3.257768
H -2.045544 3.077636 5.569084
H -1.280114 1.003163 6.713923
H 0.216591 -0.594162 5.521215
H 0.907614 -0.135648 3.181878
H 2.762393 -0.968305 -1.325398
H 3.203278 -2.881589 -2.868136
H 1.308329 -4.455612 -3.404117
H -0.939209 -4.018865 -2.390207
H -2.246723 -2.813127 -0.638194
H -2.123096 -1.234533 -1.432823
H -3.740892 -0.184944 -0.801710
H -5.731557 1.130077 -0.217643

H -6.109625 1.881204 2.128076
H -4.458153 1.276915 3.899769
H -2.451102 -0.033668 3.332630
H -0.547002 1.148598 -1.111114
C 1.722034 3.186175 1.355310
H 2.594268 2.626857 1.721463
H 1.920906 3.457359 0.307484
C -1.286505 -2.432911 2.103447
H -0.889103 -2.009795 3.036955
H -0.513205 -3.111929 1.711370
C 1.472307 4.381030 2.217973
C 0.913665 5.555217 1.694241
N 1.772811 4.250987 3.518628
C 0.641813 6.615641 2.554562
H 0.711951 5.639202 0.623337
C 1.513975 5.275237 4.331347
C 0.941538 6.474497 3.907250
H 0.206564 7.540931 2.171097
H 1.776712 5.136616 5.386800
H 0.746925 7.278161 4.619181
C -2.575711 -3.155455 2.340989
C -3.278014 -2.989627 3.542333
N -3.033351 -3.934943 1.349913
C -4.489602 -3.652373 3.713638
H -2.871555 -2.352905 4.332210
C -4.195727 -4.564666 1.524511
C -4.964851 -4.459096 2.682642
H -5.053947 -3.540698 4.641992
H -4.536815 -5.193767 0.694324
H -5.909339 -4.998217 2.771399

78

3-[Ni-H]$^{+}$(M06-L)
Ni 0.317380 0.023768 0.059486
P 0.584914 1.690758 1.332029
P -1.359086 -1.038221 1.002686
N -0.804741 1.480622 -1.296637
N 0.422681 -1.536444 -1.164838

C -1.634405 1.141301 -2.288671
C -2.579148 2.013076 -2.821450
C -2.661211 3.298499 -2.289301
C -1.779196 3.666906 -1.276740
C -0.847176 2.735340 -0.810383
C 0.188166 3.097865 0.213544
C -0.529234 1.867487 2.765460
C -1.659610 2.695261 2.746826
C -2.496934 2.766610 3.860035
C -2.217538 2.015631 5.000670
C -1.101717 1.178255 5.023893
C -0.267447 1.099759 3.911216
C 1.504782 -1.748008 -1.944157
C 1.579202 -2.781495 -2.864501
C 0.492690 -3.645544 -2.992927
C -0.622804 -3.435970 -2.192229
C -0.638155 -2.374289 -1.285867
C -1.830540 -2.116702 -0.417153
C -2.917534 -0.315393 1.590735
C -3.599977 0.569220 0.739046
C -4.771574 1.192994 1.155873
C -5.274167 0.949939 2.435769
C -4.598350 0.082283 3.291254
C -3.424756 -0.547343 2.875490
H -1.542098 0.117617 -2.671156
H -3.238858 1.685428 -3.625757
H -3.399096 4.010690 -2.663775
H -1.802669 4.672195 -0.851316
H -0.064663 4.011743 0.766748
H 1.136318 3.318230 -0.305602
H -1.902167 3.288896 1.861290
H -3.377728 3.412139 3.830373
H -2.876038 2.075112 5.870134
H -0.878161 0.583010 5.912006
H 0.608003 0.442702 3.945928
H 2.334979 -1.054635 -1.804948
H 2.481133 -2.904964 -3.464506
H 0.516813 -4.473483 -3.703783

H -1.491648 -4.093175 -2.253851
H -2.297872 -3.059801 -0.096092
H -2.602336 -1.572317 -0.988928
H -3.212154 0.773016 -0.262732
H -5.291257 1.874778 0.478451
H -6.191363 1.441839 2.767000
H -4.981424 -0.105555 4.296893
H -2.909404 -1.217840 3.566997
H 1.677729 0.566954 -0.309686
C 2.240218 2.100402 2.039130
H 2.620249 1.199145 2.540341
H 2.890167 2.270450 1.167387
C -0.851724 -2.238976 2.306659
H -0.626377 -1.665329 3.218438
H -1.688907 -2.920251 2.519056
C 2.235672 3.283011 2.957984
C 2.417095 3.130875 4.338426
N 2.007740 4.479014 2.395855
C 2.352777 4.255708 5.155857
H 2.604286 2.141803 4.762681
C 1.950969 5.550193 3.187531
C 2.112262 5.496979 4.571775
H 2.488968 4.162865 6.235577
H 1.765423 6.511090 2.693485
H 2.051768 6.406122 5.172230
C 0.342877 -3.007448 1.829431
C 1.637027 -2.490119 1.982032
N 0.099014 -4.150868 1.178117
C 2.708935 -3.189602 1.438199
H 1.793802 -1.557150 2.527926
C 1.136093 -4.813870 0.658442
C 2.455966 -4.377591 0.753811
H 3.728162 -2.811214 1.543332
H 0.901440 -5.745224 0.129634
H 3.264361 -4.956896 0.304279

79

3-[Ni-H-NH]$^{2+}$(M06-L)

Ni 0.247994 0.056992 0.051839
P 0.040064 1.914030 1.098285
P -1.485773 -1.081385 0.862908
N -0.227828 2.454211 -2.021047
N 0.486653 -1.470482 -1.152132
C -0.015409 2.606083 -3.339620
C -0.419643 3.766906 -3.967813
C -1.025139 4.762356 -3.198573
C -1.222361 4.570337 -1.834802
C -0.820302 3.378559 -1.235203
C -1.078173 3.080761 0.203948
C -0.707406 1.823301 2.752565
C -1.692917 2.696998 3.225239
C -2.173599 2.563063 4.527834
C -1.673144 1.567369 5.365370
C -0.686355 0.696005 4.900382
C -0.207972 0.822742 3.599341
C 1.672741 -1.715676 -1.743752
C 1.889124 -2.824444 -2.547010
C 0.840179 -3.717953 -2.752553
C -0.385946 -3.466935 -2.146955
C -0.544369 -2.331448 -1.353092
C -1.841153 -1.995939 -0.695467
C -3.013235 -0.240898 1.350873
C -3.704546 0.524430 0.397607
C -4.829829 1.259012 0.766087
C -5.273918 1.243766 2.089128
C -4.585027 0.496033 3.043205
C -3.459145 -0.240091 2.679824
H 0.483823 1.779822 -3.844034
H -0.252865 3.890637 -5.036493
H -1.345279 5.695101 -3.664837
H -1.695596 5.337638 -1.222446
H -2.088977 2.640556 0.288085
H -1.129066 4.032626 0.753462
H -2.104274 3.486840 2.592606
H -2.950164 3.242135 4.886377
H -2.054916 1.467392 6.383611

H -0.289706 -0.086055 5.551717
H 0.570575 0.143457 3.235730
H 2.463952 -0.989903 -1.553656
H 2.868420 -2.979097 -2.999760
H 0.975478 -4.602273 -3.377590
H -1.230412 -4.143505 -2.282509
H -2.463636 -2.879769 -0.504975
H -2.420121 -1.320119 -1.346299
H -3.360900 0.559505 -0.641250
H -5.360576 1.846695 0.014278
H -6.155619 1.820288 2.376480
H -4.917945 0.489786 4.083437
H -2.920375 -0.800099 3.448022
H 1.343459 0.820555 -0.635835
H 0.142699 1.593841 -1.556010
C 1.596681 2.877901 1.361828
H 2.329290 2.157316 1.751612
H 1.945598 3.190519 0.365965
C -1.123323 -2.372593 2.117046
H -0.598039 -1.898411 2.957899
H -0.364517 -3.012288 1.633283
C 1.435911 4.022830 2.312450
C 1.057692 5.296454 1.868094
N 1.623675 3.744118 3.610663
C 0.844287 6.301744 2.808300
H 0.945336 5.501352 0.800327
C 1.421922 4.714957 4.500379
C 1.021098 6.006644 4.157318
H 0.547405 7.302799 2.488500
H 1.589048 4.453529 5.552091
H 0.863182 6.761604 4.929141
C -2.278707 -3.197385 2.614734
C -2.364402 -3.538553 3.970342
N -3.202362 -3.576420 1.724033
C -3.445657 -4.298604 4.405607
H -1.596025 -3.203650 4.671323
C -4.235832 -4.303639 2.150271
C -4.407825 -4.691145 3.477884

H -3.538002 -4.575361 5.457974
H -4.972589 -4.592580 1.392328
H -5.275477 -5.282591 3.774286
